# An open-source, citizen science and machine learning approach to analyse subsea movies

**DOI:** 10.3897/BDJ.9.e60548

**Published:** 2021-02-24

**Authors:** Victor Anton, Jannes Germishuys, Per Bergström, Mats Lindegarth, Matthias Obst

**Affiliations:** 1 Wildlife.ai, New Plymouth, New Zealand Wildlife.ai New Plymouth New Zealand; 2 Combine AB, Gothenburg, Sweden Combine AB Gothenburg Sweden; 3 Department of Marine Sciences, Göteborg University, Gothenburg, Sweden Department of Marine Sciences, Göteborg University Gothenburg Sweden; 4 SeAnalytics AB, Gothenburg, Sweden SeAnalytics AB Gothenburg Sweden

**Keywords:** marine biodiversity, autonomous underwater vehicles, remotely-operated vehicles, artificial intelligence, big data, image analysis, participatory science, Essential Biodiversity Variables, research infrastructure, biodiversity monitoring

## Abstract

**Background:**

The increasing access to autonomously-operated technologies offer vast opportunities to sample large volumes of biological data. However, these technologies also impose novel demands on ecologists who need to apply tools for data management and processing that are efficient, publicly available and easy to use. Such tools are starting to be developed for a wider community and here we present an approach to combine essential analytical functions for analysing large volumes of image data in marine ecological research.

**New information:**

This paper describes the Koster Seafloor Observatory, an open-source approach to analysing large amounts of subsea movie data for marine ecological research. The approach incorporates three distinct modules to: manage and archive the subsea movies, involve citizen scientists to accurately classify the footage and, finally, train and test machine learning algorithms for detection of biological objects. This modular approach is based on open-source code and allows researchers to customise and further develop the presented functionalities to various types of data and questions related to analysis of marine imagery. We tested our approach for monitoring cold water corals in a Marine Protected Area in Sweden using videos from remotely-operated vehicles (ROVs). Our study resulted in a machine learning model with an adequate performance, which was entirely trained with classifications provided by citizen scientists. We illustrate the application of machine learning models for automated inventories and monitoring of cold water corals. Our approach shows how citizen science can be used to effectively extract occurrence and abundance data for key ecological species and habitats from underwater footage. We conclude that the combination of open-source tools, citizen science systems, machine learning and high performance computational resources are key to successfully analyse large amounts of underwater imagery in the future.

## Introduction

Biological observation techniques in the marine environment need to improve radically to serve our understanding of marine ecosystems under the influence of multiple stressors including long-term global change (Benedetti-Cecchi et al. 2018). Over the last decade, biologists have gained an increased access to autonomously operated technologies for data collection, offering the opportunity to generate enormous volumes of data. This is especially the case for high-definition optical imagery recorded by ROV’s (remotely-operated vehicles), AUVs (autonomous underwater vehicles), drop-cameras, video plankton recorders and drones (Bean et al. 2017, Danovaro et al. 2016). Although such image-based observations may revolutionise the fields of marine biology and biodiversity monitoring, these methods also impose completely new demands for data management and processing on researchers.

In-situ monitoring systems need to be coupled to data services that allow for swift exploration, processing and long-term storage (Guidi et al. 2020). Some of these services already exist, for example, the Global Reef Record and CoralNet, which allow researchers to host and analyse images of coral reefs (Beijbom et al. 2015), EcoTaxa that offers analysis of large amounts of plankton imagery (Picheral et al. 2017) and FathomNet, which offers machine learning algorithms and training data to analyse deep-sea footage. Although these platforms have pioneered the daily use of image analysis tools in marine science, they may not be able to provide all the functionalities needed by the fast-growing community of users. Some of these sought-after functions include seamless connectivity with project-specific data archives, the involvement of non-scientific audiences in environmental research, modules that can be easily updated to include state-of-the-art analytical tools and versatile systems that researchers can easily adapt to fit to different types of data and purposes.

Here, we present the Koster Seafloor Observatory, an open-source modular approach for managing, processing, and analysing large amounts of subsea movie data for marine ecological research. The Koster Seafloor Observatory allows scientists to upload underwater footage to a customised citizen science website and then train machine learning algorithms with those classifications provided by citizen scientists. These algorithms can be accessed through an Application Programming Interface (API) allowing researchers to test the performance of the algorithms under different confidence and overlapping thresholds, share their models with a wider audience and extract species observations from new footage.

## Project description

### Title

Mapping cold water corals in Sweden's first marine national park

### Study area description

We piloted the Koster Seafloor Observatory to extract data on spatiotemporal distribution and relative abundance of habitat-building species from deep-water recordings in a Marine Protected Area, the Kosterhavets National Park in Sweden. The Park, established in 2009, contains a highly diverse and unique marine ecosystem. The seafloor in the deeper waters of the Park has oceanic connections and hence contains much of the bottom-dwelling fauna, which is otherwise only found in deep oceanic waters (Lavaleye et al. 2009). This fauna includes large habitat-building species (Costello et al. 2005), such as sponges (e.g. *Geodia baretti*, *Phakellia ventilabrum*) and cold water corals (e.g. *Desmophyllum pertusum*), as well as other large species which can be easily identified from camera footage (e.g. starfish *Porania pulvillus*, *Crossaster papposus*, *Echinus esculentus*).

### Design description

**Figure 1.**
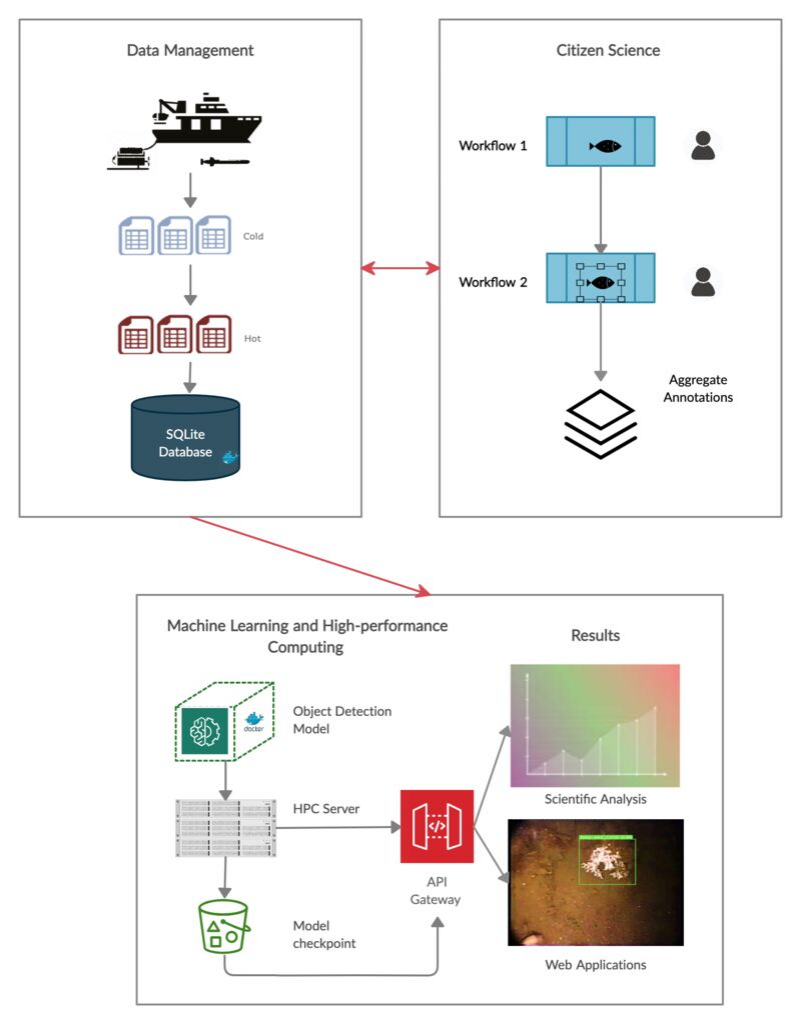
High-level overview of the three main modules and the components of the Koster Seafloor Observatory.

The Koster Seafloor Observatory is divided into three main modules: data management, citizen science and machine learning with high performance computing (Fig. 1).

**Module 1: Data management (Anton et al. 2019)**

In the data management module, researchers store and process the data in a way that maximises efficiency, convenience and opportunities for sharing and collaboration. To store and access the raw data, we use long-term and short-term storage servers. The long-term storage server, or cold storage, archives large amounts of files that need not be accessed frequently. In our case, these include recordings from Remotely-Operated Vehicles (ROVs) managed by the University of Gothenburg, Sweden. The movies (mp4 and mov formats) are on average 1-2 hours long and have been systematically collected from all expeditions since the late 1990s (Fig. 1). The metadata associated with these movies is regularly published in the Swedish National Data Archive.

The short-term storage server, or hot storage, stores a small proportion of files that are frequently used for analysis. Here, we transferred 60 movies from the cold storage to a project-specific short-term storage server (Suppl. material 2). The number of movies we selected was a compromise between selecting a representative sample and efficiently using the limited storage of our server. This "hot server" was Linux-based and hosted by Chalmers University of Technology, Gothenburg. The specifications of this High Performance Computing server consisted of a GTX2080Ti GPU with 2 x 8 core Intel(R) Core(TM) i9-9900 CPU @ 3.10GHz (total 16 cores) and 2GB DDR4 RAM.

**Figure 2.**
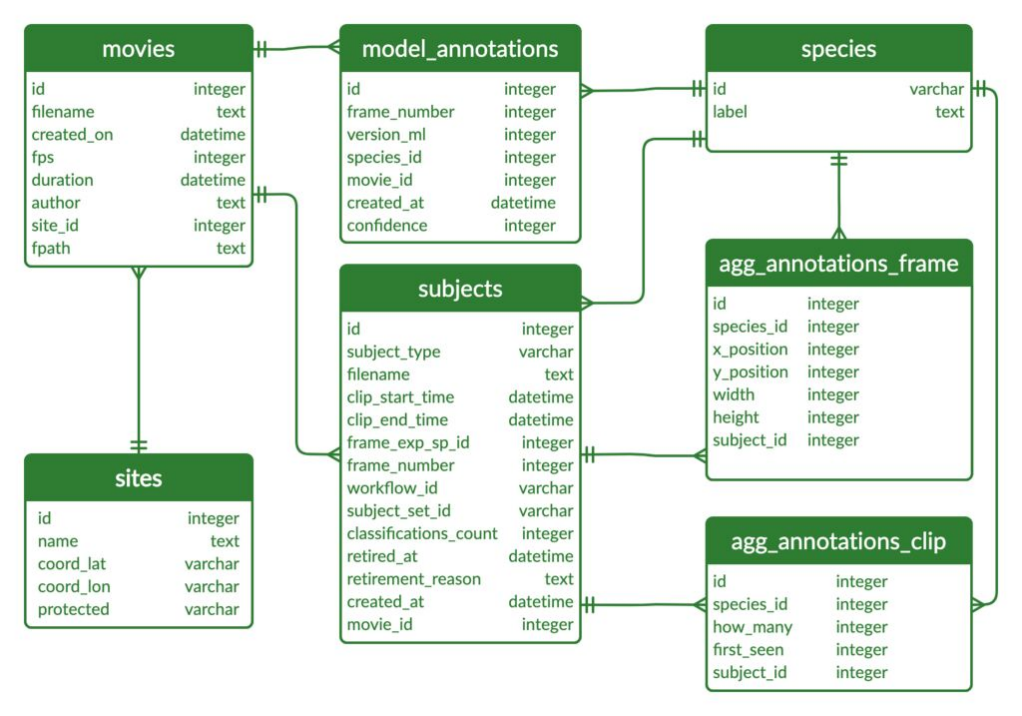
Entity relationship diagram of the SQLite database used by the Koster Seafloor Observatory.

We created a SQLite database to link all information related to the movies and the classifications provided by both citizen scientists and machine learning algorithms (Fig. 1). The database has seven interconnected tables (Fig. 2). The “movies”, “sites” and “species” tables have project-specific information from the underwater movie metadata, as well as the species choices available for citizen scientists to annotate the clips, retrieved from Zooniverse. The “agg_annotations_frame” and “agg_annotations_clip” tables contain information related to the annotations provided by citizen scientists. The “subjects” table has information related to the clips and frames uploaded to the Koster Seafloor Observatory. The "model_annotations" table holds information related to the annotations provided by the machine learning algorithms. The database followed the Darwin Core (DwC) standards to maximise the sharing, use and reuse of open-access biodiversity data.

**Module 2: Citizen science (Anton et al. 2019)**

In the citizen science module, researchers and citizen scientists work together to efficiently and accurately annotate raw data. To identify the species recorded in our footage, we created a citizen science website. The site is hosted in Zooniverse, the largest citizen science platform in the world. The website contains rich supporting material (e.g. background, tutorials, field guides) and features two workflows that help citizen scientists to classify biological objects in video (workflow 1) and locate these objects in still images (workflow 2).

**Figure 3.**
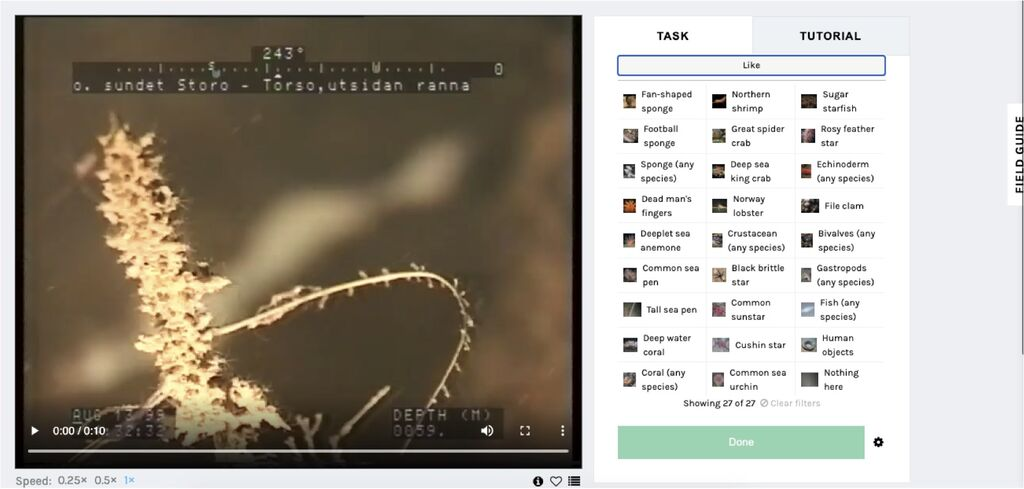
Screenshot of the Zooniverse annotation interface. On the left, display of the clips. On the right, species choices available.

Workflow 1 (species identification):

Citizen scientists are presented with 10-second clips of underwater footage and need to select at least one of the 27 available choices (Fig. 3). The choices include species of scientific importance, animals grouped at different taxonomic levels (e.g. “gastropods” or “fish”), as well as a few miscellaneous options (“Nothing here”, “Human objects”). If citizen scientists select a species or animal, they also need to specify the number of individuals of the taxon selected and the time (in seconds) when any of the individuals fully appears on the screen.

We compared the classifications provided by an expert to those provided by citizen scientists to estimate the accuracy of citizen scientists to identify cold water corals (Table 1). A total of 2,594 clips were classified both by an expert and by eight different citizen scientists. We aggregated the classifications provided by citizen scientists on a per-clip basis and retained the classifications of cold water corals and grouped the rest of classifications into "Other". For this case study, we chose cold water corals (*Desmophyllum pertusum*) because this species has a crucial ecological role in the study site (Costello et al. 2005). We used a confusion matrix to understand how agreement amongst citizen scientists correlates to the accuracy of their aggregated classifications (e.g. an agreement threshold of 80% corresponds to an agreement on the classifications of at least seven of the eight citizen scientists who annotated the clip). "Adequate" accuracy of citizen scientists with respect to experts depends on multiple parameters, including the type of data classified, the classification tool and the research questions (Aceves-Bueno et al. 2017). In our study, we decided that at least 80% of agreement amongst citizen scientists was an appropriate accuracy threshold as it minimised the number of false positives citizen scientists provide.

**Figure 4.**
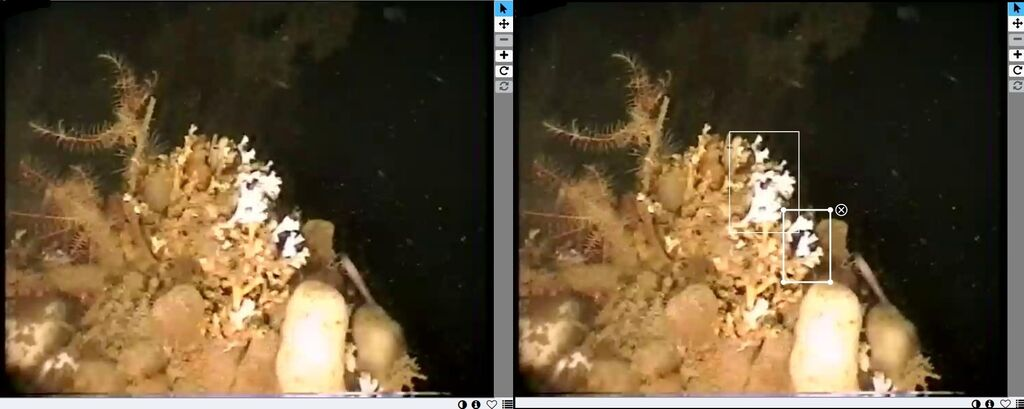
Example of a frame containing cold water coral displayed to the citizen scientists (left) and the same frame with annotated rectangles provided by a citizen scientist (right).

Workflow 2 (object location):

Citizen scientists are presented with a still image of the species of interest. To annotate the image, citizen scientists need to draw rectangles around the individuals of the species (Fig. 4). If citizen scientists are not able to identify any individual of the species of interest in the frame, they will not draw any rectangle. Each still image is annotated by at least five different citizen scientists before it is “retired” from the website.

**Figure 5.**
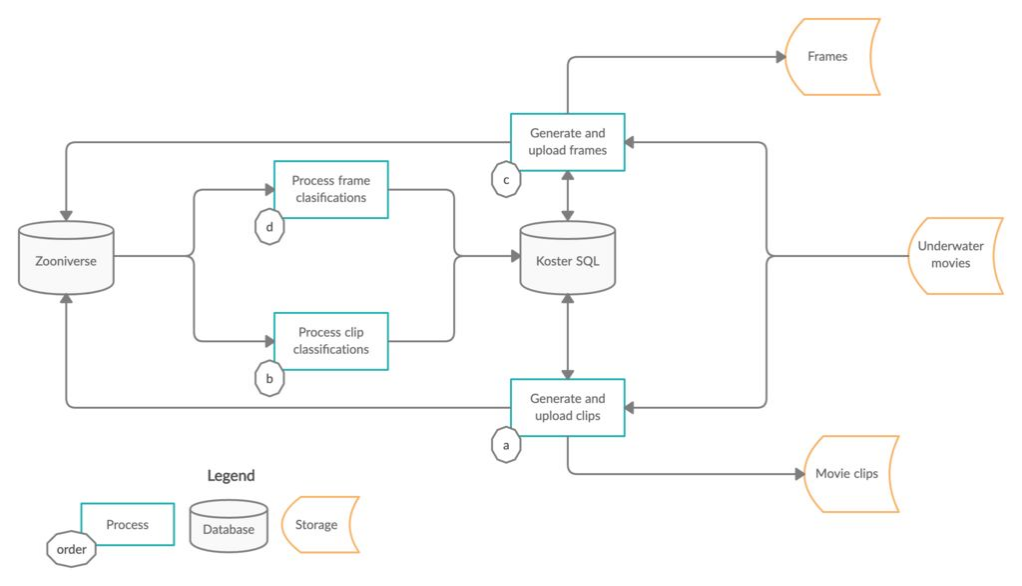
Four-stage video processing framework used to identify species of interest.

We used a four-stage video processing framework to upload clips and still images to the Koster Seafloor Observatory and download the annotations provided by citizen scientists (Fig. 5).

- **Stage 1**: Generate and upload clips (Fig. 5, circle a). In this stage, we split the +1 hour long movies into 10-second clips. After the clips were created, we randomly selected 5,702 clips from the original 60 movies and uploaded them to workflow 1 of the Koster Seafloor Observatory.
- **Stage 2**: Process clip annotations (Fig. 5, circle b). We retrieved the annotations provided by citizen scientists in workflow 1 and aggregated them on a per-clip basis. To aggregate workflow 1 annotations, we grouped the annotations each clip received and retained only those choices that were selected by at least 80% of the citizen scientists who annotated the clip. In our study, there were 194 clips for which cold-water coral was identified at least by 80% of the citizen scientists. We also averaged the answers from citizen scientists to the question "When is the first time the species appears fully in the video?".
- **Stage 3**: Generate and upload frames (Fig. 5, circle c). We extracted up to three frames per clip from the 194 clips containing cold water corals and extracted one frame per second after the first time the species fully appeared in the clip. After extracting 533 frames, we then uploaded them to workflow 2 of the Koster Seafloor Observatory. Five different citizen scientists per frame annotated the location of cold water corals in the still images.
- **Stage 4**: Process frame annotations (Fig. 5, circle d). We retrieved workflow 2 annotations provided by citizen scientists and aggregated them on a per-frame basis. To aggregate workflow 2 annotations, we retained the area of overlapping between those rectangles drawn by 80% of the citizen scientists who annotated the frame. A total of 409 of the 533 frames had matching rectangles drawn by 80% of the citizen scientists. We formatted the aggregated annotations appropriately to train YOLOv3 algorithms (Redmon and Farhadi 2018)

**Module 3: Machine learning and High Performance Computing (Germishuys et al. 2019)**

In the machine learning and High Performance Computing module, researchers train, test and expose state-of-the-art machine learning models. The aggregated citizen scientist annotations are used to train object-detection models that track and identify the species of interest. In our case study, we used 409 user-annotated ground-truth frames obtained from workflow 2 (Suppl. material 1) to train an algorithm to identify cold water corals. We augmented this data by using a frame tracker which filled subsequent movie frames with bounding boxes with the highest probability of containing the object of interest. This typically increased the amount of data by a factor of 10. The frames were then pre-processed to remove background distortion because colours often lose intensity underwater, mainly due to poor visibility. Three datasets were then created, one for training the model, another for validation (which is used to tune the model hyperparameters) and, finally, a testing set. Once the data were prepared, the model training was done until satisfactory metrics were achieved on evaluation measures (i.e. F1 = 0.970, Recall = 0.962, Precision = 0.979 and mAP@0.5 = 0.962).

We made the trained model available through an application programming interface (API), where it can be used by researchers to run predictions of the species of interest in new recordings (Fig. 1). To this end, we used FastAPI (Ramírez 2020) as it provides the speed, scalability and reliability required to have multiple users making use of the service at the same time. The API was also supplied with a user-friendly front-end, using the Streamlit (Teixeira 2020) framework, allowing a broader audience of scientific users (i.e. ecologists, ROV and AUV-pilots, students) to access the service through a web application. The interface allows researchers to browse through already-classified footage or to upload their own footage as either images or video. Once the media has been uploaded/selected, users are able to manipulate hyperparameter thresholds (IOU threshold, confidence threshold) and interactively see the impact on the model output. The API is described by Germishuys et al. (2019).

We compared manual observations of cold water corals provided by an expert to those provided by our machine learning model to estimate the accuracy of the model under different confident thresholds (Table 2). Both expert and model classified movies corresponding to 132 squares of a spatial grid within the National Park into "Coral" and "No coral" (i.e. presence/absence of cold water corals). To estimate the final classifications of the machine learning model, we aggregated the raw model output, containing coral observations for each frame under 0.5, 0.7 and 0.9 confidence thresholds, into periods in which the species was continuously observed with > 50% overlap between consecutive bounding boxes. These aggregated observation periods described the first and last frame in which coral was visible (Suppl. material 3). If aggregated observation periods were within the footage corresponding to one square, the square was classified as Coral. We used confusion matrices to estimate the accuracy of the machine-based classifications under the different thresholds. The best accuracy for our case study was achieved with a confidence threshold of 0.7.

**Figure 6.**
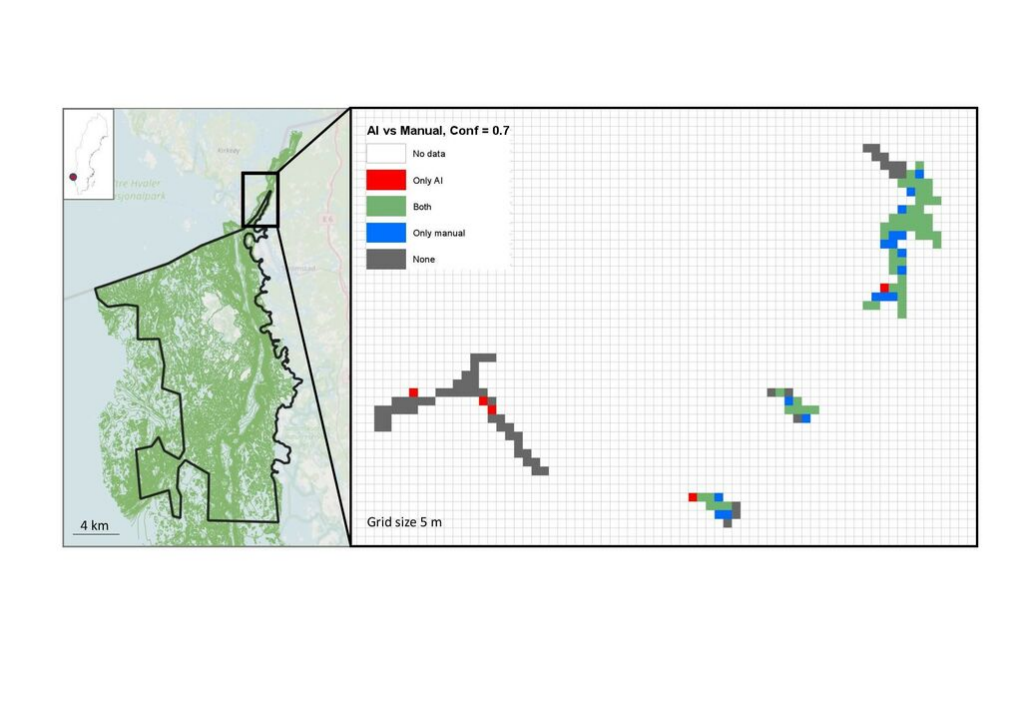
Comparison of manual and machine learning model-based spatial distribution of cold water coral in the reef area Säcken in Kosterhavets National Park, Sweden. Spatial distribution is based on coral observations in ROV movies corresponding to 132 squares of the spatial grid. Confidence threshold (Conf) for the model is set to 0.7. Grid size 5 m.

The last component of this module is a data visualisation toolkit that enables researchers to explore and visualise the ecological data extracted from the outputs of the machine learning model. In our case, we mapped the cold water coral annotations provided by the expert and the machine learning model with a 0.7 confidence threshold (Fig. 6). Our results highlight that machine learning models with a relatively high confidence threshold are well-suited for automated monitoring of cold water coral over large areas.

**Discussion**

The functionalities of the Koster Seafloor Observatory have been tested in the present case study, which illustrates the scientific potential of this open-source and modular approach. Our approach can be used to extract ecological data on abundance and distribution for many benthic species from underwater recordings. Underwater footage is today routinely collected by many research institutes, which may allow for a concerted analysis of such data over broad spatial and temporal scales in the future. Such analyses may calculate data products for biological state variables on regional or even global level, so-called Essential Biodiversity Variables or EBVs (Pereira et al. 2013, Hardisty et al. 2019). A recent study by Kissling et al. (2018) suggests that image-based sensor networks are promising candidates for EBVs, while many other studies highlight the potential of these methods for marine monitoring programmes (Mack et al. 2020, Lopez-Vazquez et al. 2020). Our case study provides empirical support that these methods are ready for implementation in national monitoring programmes and that useful data products can be derived from image-based sensors, especially in marine environments which are particularly difficult to access and survey.

In order to scale up analysis of underwater imagery in the future to extract ecological data for larger regions, longer time periods and more species, several technical bottlenecks have to be addressed. Data archiving functions can fall under organisational or governmental responsibilities and may not be fulfilled by a single global system. Consequently, most underwater recordings are currently locally archived and cannot be discovered. Here, further work is needed to promote the use of open interoperable archives and data portals (e.g. European Marine Data Archive, EMODnet portal) that enable researchers to adequately publish metadata associated with underwater recordings. Another important technical bottleneck is the disconnection between many essential data services that need to interact to successfully analyse image data. We suggest that seamless links should be developed especially between citizen science platforms (for training of machine learning models) and high-performance computation services (for extracting ecological data from large amounts of imagery). Regional, national and global research infrastructures should take a leading role in this development to overcome current technical challenges.

### Funding

The project was funded by Ocean Data Factory, an expert network supported by grants from Sweden’s Innovation Agency (grant agreement no. 2019-02256), the Swedish Agency for Marine and Water Management (grant agreement no. 956-19) and the Swedish Research Council (through Swedish LifeWatch grant agreement no. 829-2009-6278). The presented work was furthermore supported by the NeIC programme DeepDive and the Horizon 2020 project ENVRIplus (grant agreement no. 654182).

## Web location (URIs)

Homepage: https://www.zooniverse.org/projects/victorav/the-koster-seafloor-observatory/about/results

## Usage licence

### Usage licence

Creative Commons Public Domain Waiver (CC-Zero)

### IP rights notes

Our approach is open for use in research, as well as public and academic education for analysis of community composition in marine ecosystems.

## Supplementary Material

3CB3F7B3-5BA5-56EE-850E-573B2B14825A 10.3897/BDJ.9.e60548.suppl1 Supplementary material 1 Dataset of underwater images of *Desmophyllum pertusum* Data type images, zipped Brief description Instances of *Desmophyllum pertusum* used to train Koster YOLO machine learning model. File: oo_502920.zip https://binary.pensoft.net/file/502920 Victor Anton, Jannes Germishuys, Per Bergström, Mats Lindegarth, Matthias Obst

B6A7199B-7118-582C-B17E-7E778469B5EB 10.3897/BDJ.9.e60548.suppl2 Supplementary material 2 Metadata for movies used in the case study Data type table with occurrences Brief description This file contains metadata from the movies used to test the model and illustrate its application. To access the movie data files, contact the authors or search the filenames in the Swedish National Data Service: https://snd.gu.se/en/catalogue/study/snd1069. File: oo_508903.xlsx https://binary.pensoft.net/file/508903 Victor Anton, Jannes Germishuys, Per Bergström, Mats Lindegarth, Matthias Obst

8729D25C-94DE-50DA-A5A2-EA51252DDA2E 10.3897/BDJ.9.e60548.suppl3 Supplementary material 3 model results Data type table Brief description Model output from analysis of the selected movies in Supplementary material 2. Explanation of variables: FilenameInThisStudy (movieID), frame_no_start (frame number when the object was detected for the first time), frame_no_end (frame number when the object was detected for the last time), max_conf (highest confidence value achieved by the object throughout the consecutive frames), x (x-position of the upper-left corner of the bounding box with the highest confidence value), y (y-position of the upper-left corner of the bounding box with the highest confidence value), w (width of the bounding box with the highest confidence value), h (height of the bounding box with the highest confidence value). File: oo_502950.csv https://binary.pensoft.net/file/502950 Victor Anton , Jannes Germishuys , Per Bergström , Mats Lindegarth , Matthias Obst

## Figures and Tables

**Table 1.** Confusion matrices derived from applying different citizen scientists agreement thresholds (Cit.Sci. Agr.) when comparing expert classifications to citizen scientist classifications of 2,594 underwater videos. Each video was classified by an expert and eight different citizen scientists. Classifications of cold water coral were retained and all other classifications were grouped as "Other". Expert classifications were compared to citizen scientist classifications with at least 80%, 60% and 40% of agreement amongst their responses (i.e. an agreement threshold of 80% corresponds to an agreement on the classifications of at least seven of the eight citizen scientists who annotated the clip).

		**Cit. Sci. Agr. ≥ 80**%		**Cit. Sci. Agr. ≥ 60**%		**Cit. Sci. Agr. ≥ 40**%	
		**Coral**	**Other**	**Coral**	**Other**	**Coral**	**Other**
**Expert**	**Coral**	111	467	315	263	475	103
	**Other**	2	2014	22	1994	84	1932

**Table 2.** Confusion matrices derived from applying different confidence thresholds (ML confidence) when overlaying manual with machine-based observations in movies corresponding to 132 squares of a spatial grid within the Kosterhavets National Park, Sweden. Detailed metadata for these recordings are provided in Suppl. material 3.

		**ML confidence = 0.5**		**ML confidence = 0.7**		**ML confidence = 0.9**	
		**Coral**	**No coral**	**Coral**	**No coral**	**Coral**	**No cora**l
**Expert**	**Coral**	54	15	52	17	28	41
	**No coral**	13	50	5	58	1	62
