# Supplementary figures and images for: An open-source, citizen science and machine learning approach to analyse subsea movies (part 2 of 2)
Source: Biodivers Data J. 2021 Feb 24;9:e60548. doi: 10.3897/BDJ.9.e60548 (PMC7930014; doi:10.3897/BDJ.9.e60548)

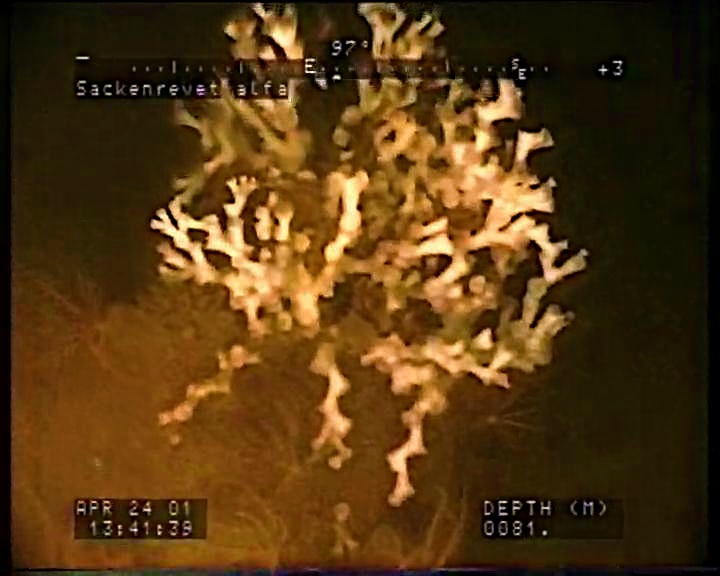

Supplement: Supplementary material 1 — Dataset of underwater images of Desmophyllum pertusum [file bdj-09-e60548-s001.zip › images_new/010424 Sa╠êckenrevet alfa Tape 74_frame_42025.jpg]

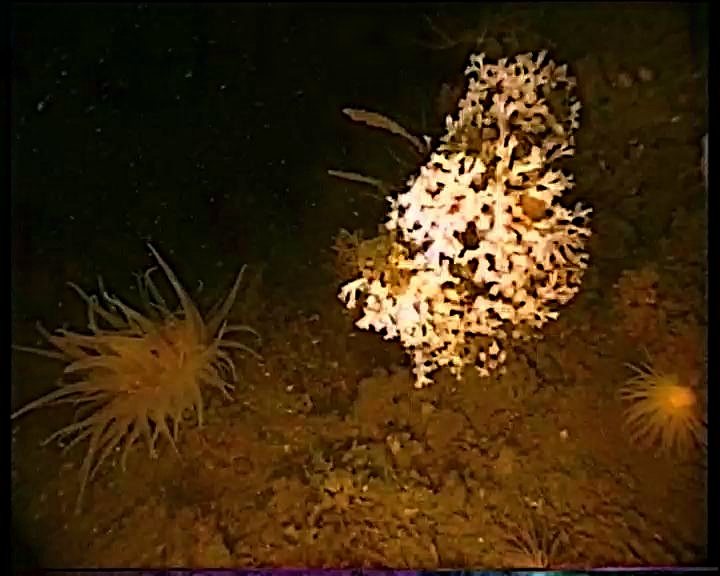

Supplement: Supplementary material 1 — Dataset of underwater images of Desmophyllum pertusum [file bdj-09-e60548-s001.zip › images_new/000114 TMBL-ROV 2000 Sa╠êckenrevet Tape 55_frame_188250.jpg]

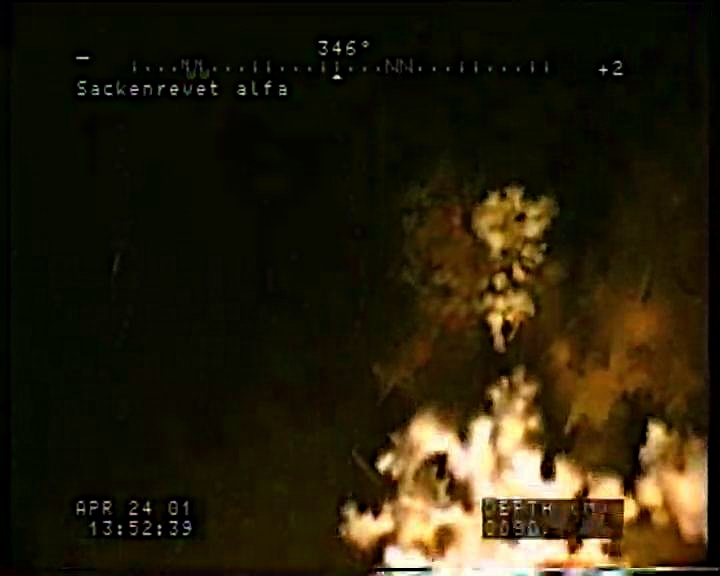

Supplement: Supplementary material 1 — Dataset of underwater images of Desmophyllum pertusum [file bdj-09-e60548-s001.zip › images_new/010424 Sa╠êckenrevet alfa Tape 74_frame_58525.jpg]

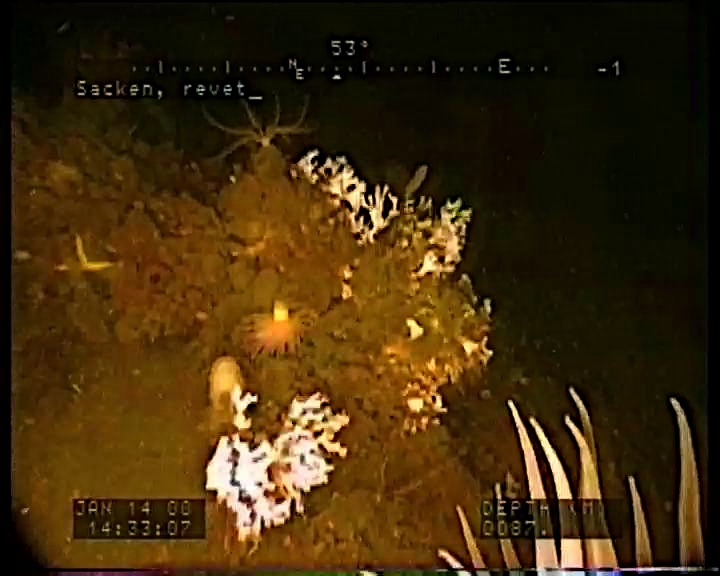

Supplement: Supplementary material 1 — Dataset of underwater images of Desmophyllum pertusum [file bdj-09-e60548-s001.zip › images_new/000114 TMBL-ROV 2000 Sa╠êckenrevet Tape 55_frame_174000.jpg]

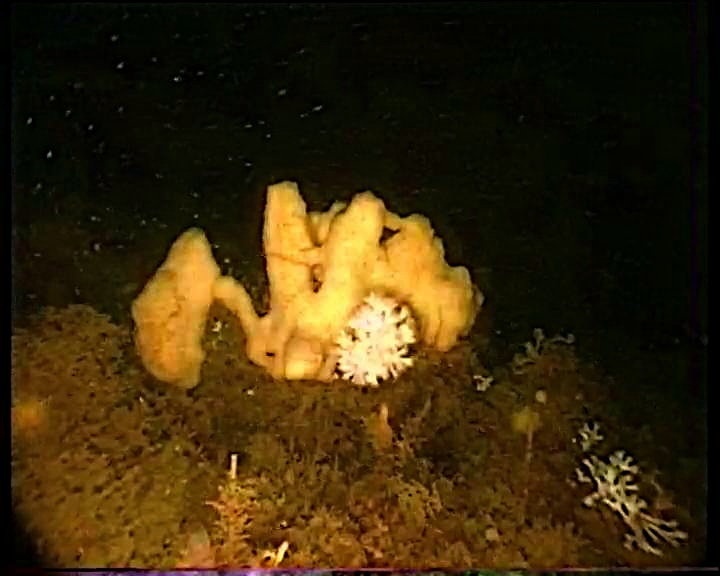

Supplement: Supplementary material 1 — Dataset of underwater images of Desmophyllum pertusum [file bdj-09-e60548-s001.zip › images_new/000114 TMBL-ROV 2000 Sa╠êckenrevet Tape 55_frame_131275.jpg]

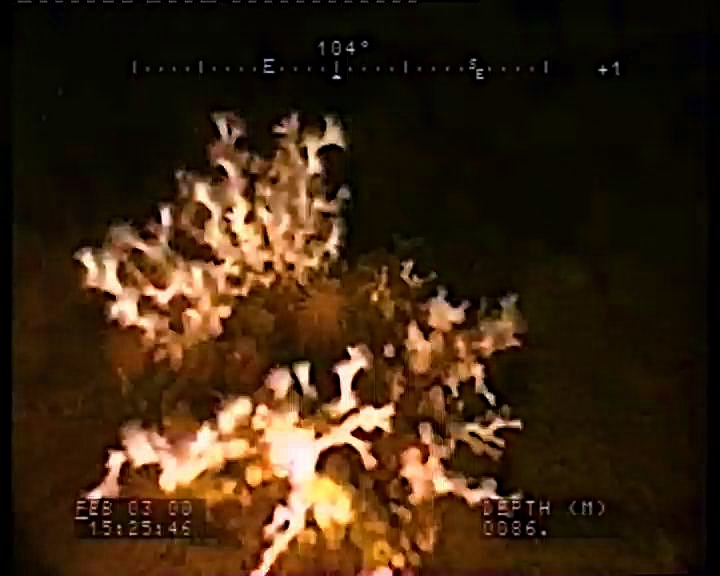

Supplement: Supplementary material 1 — Dataset of underwater images of Desmophyllum pertusum [file bdj-09-e60548-s001.zip › images_new/000203 TMBL-ROV 2000 Sa╠êcken revet EJ numrerade band_frame_750.jpg]

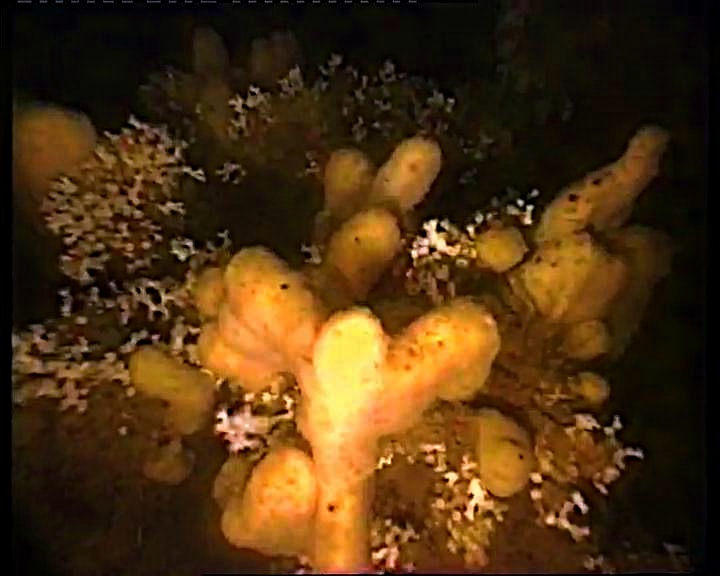

Supplement: Supplementary material 1 — Dataset of underwater images of Desmophyllum pertusum [file bdj-09-e60548-s001.zip › images_new/000203 TMBL-ROV 2000 Sa╠êcken revet EJ numrerade band_frame_31500.jpg]

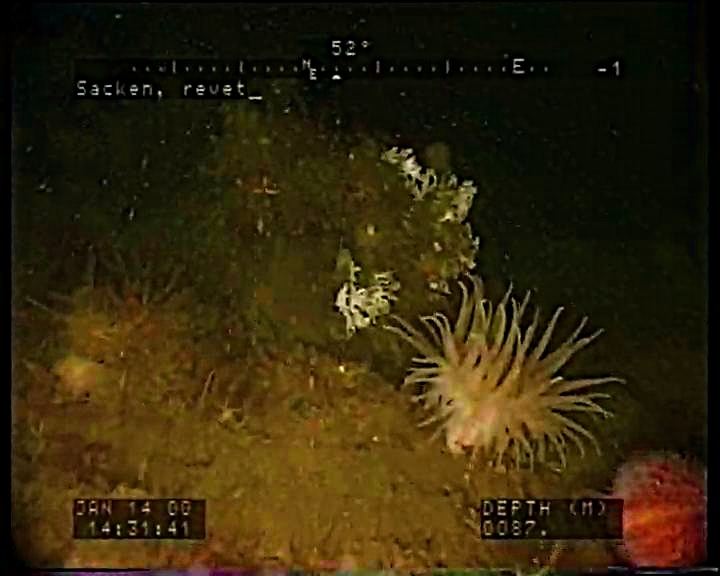

Supplement: Supplementary material 1 — Dataset of underwater images of Desmophyllum pertusum [file bdj-09-e60548-s001.zip › images_new/000114 TMBL-ROV 2000 Sa╠êckenrevet Tape 55_frame_171862.jpg]

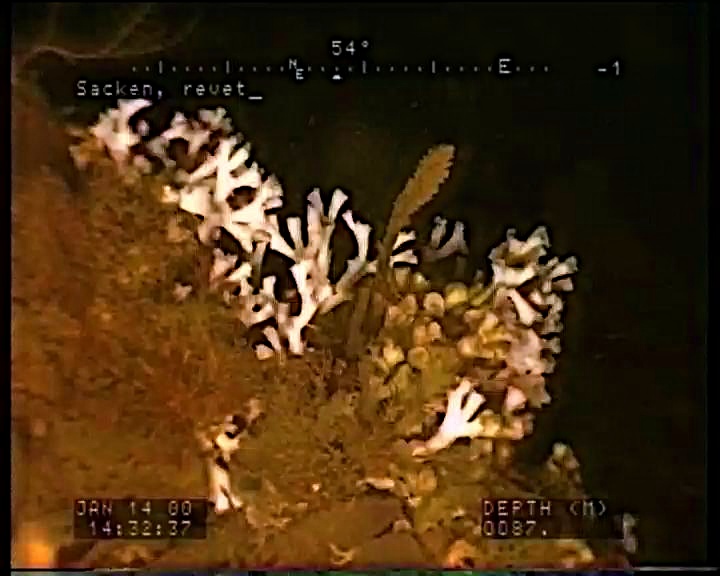

Supplement: Supplementary material 1 — Dataset of underwater images of Desmophyllum pertusum [file bdj-09-e60548-s001.zip › images_new/000114 TMBL-ROV 2000 Sa╠êckenrevet Tape 55_frame_173250.jpg]

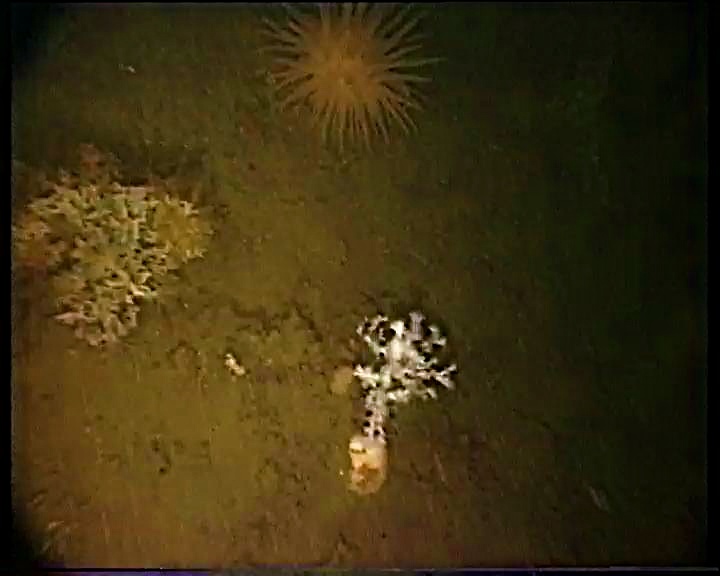

Supplement: Supplementary material 1 — Dataset of underwater images of Desmophyllum pertusum [file bdj-09-e60548-s001.zip › images_new/000203 TMBL-ROV 2000 Sa╠êckenrevet Tape 56_frame_80250.jpg]

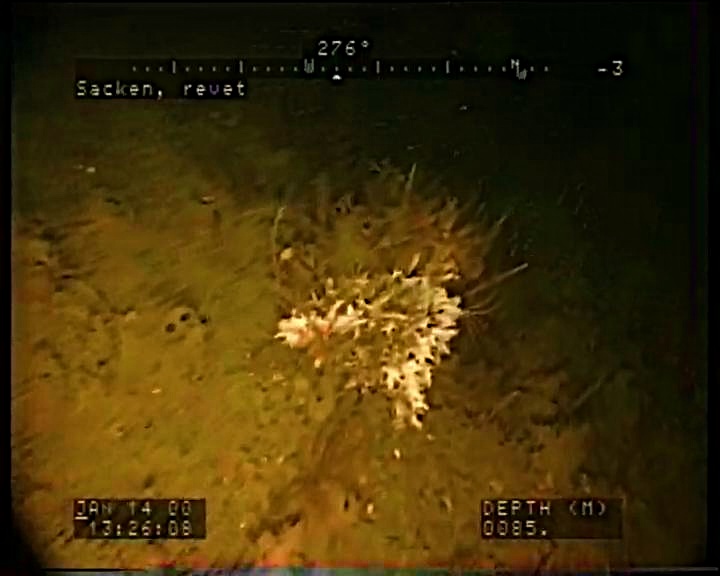

Supplement: Supplementary material 1 — Dataset of underwater images of Desmophyllum pertusum [file bdj-09-e60548-s001.zip › images_new/000114 TMBL-ROV 2000 Sa╠êckenrevet Tape 55_frame_73537.jpg]

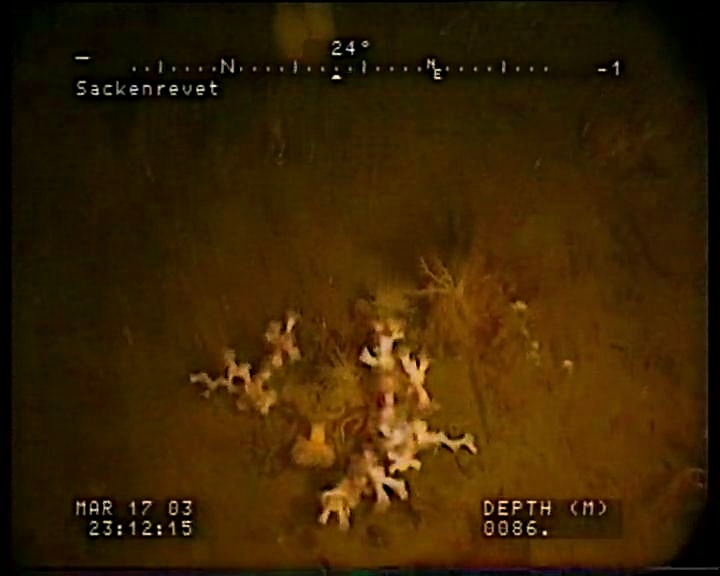

Supplement: Supplementary material 1 — Dataset of underwater images of Desmophyllum pertusum [file bdj-09-e60548-s001.zip › images_new/030317-18 TMBL-ROV 2003 Sa╠êckenrevet_frame_16525.jpg]

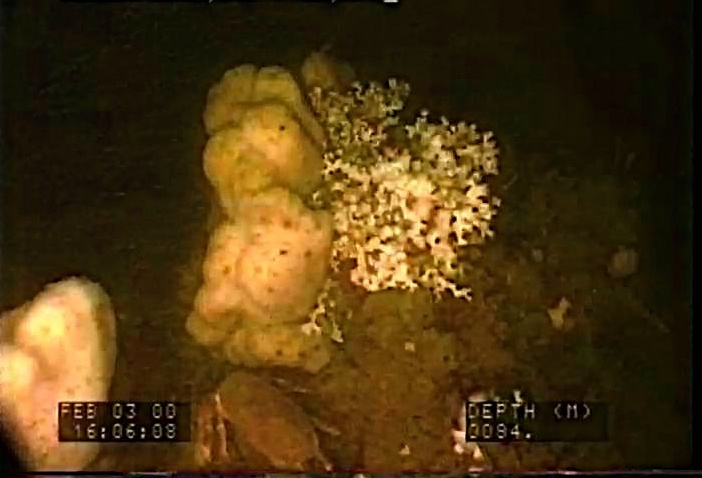

Supplement: Supplementary material 1 — Dataset of underwater images of Desmophyllum pertusum [file bdj-09-e60548-s001.zip › images_new/000203 TMBL-ROV 2000 Sa╠êckenrevet Tape 56_frame_84787.jpg]

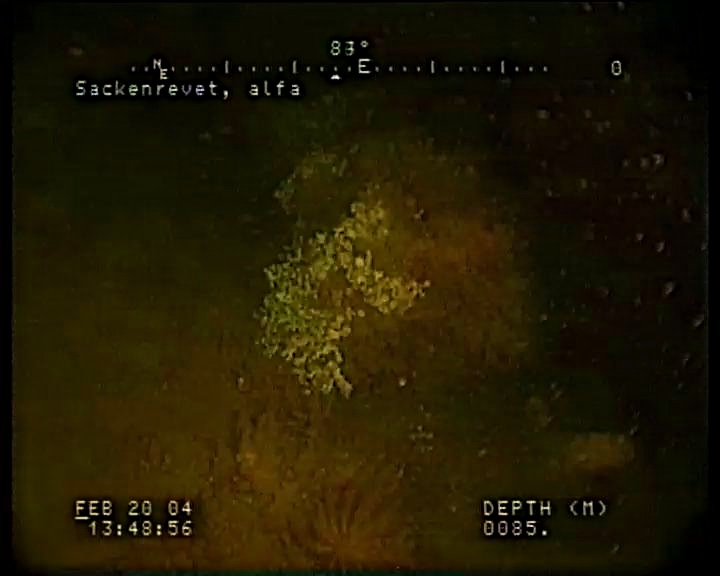

Supplement: Supplementary material 1 — Dataset of underwater images of Desmophyllum pertusum [file bdj-09-e60548-s001.zip › images_new/040220 TMBL-ROV 2004 Sa╠êckenrevet alfa_frame_18750.jpg]

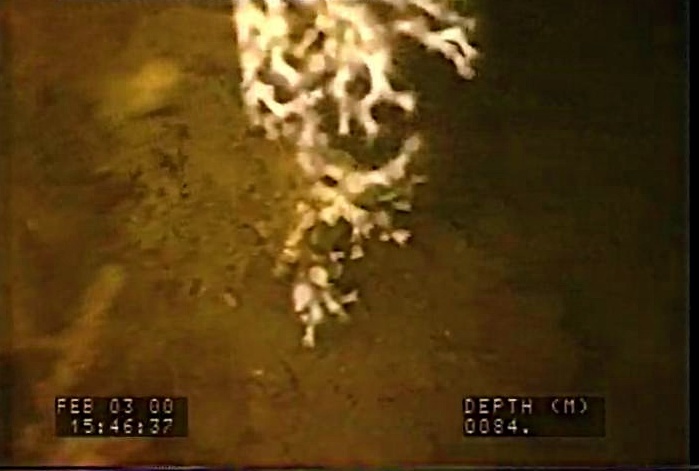

Supplement: Supplementary material 1 — Dataset of underwater images of Desmophyllum pertusum [file bdj-09-e60548-s001.zip › images_new/000203 TMBL-ROV 2000 Sa╠êckenrevet Tape 56_frame_55525.jpg]

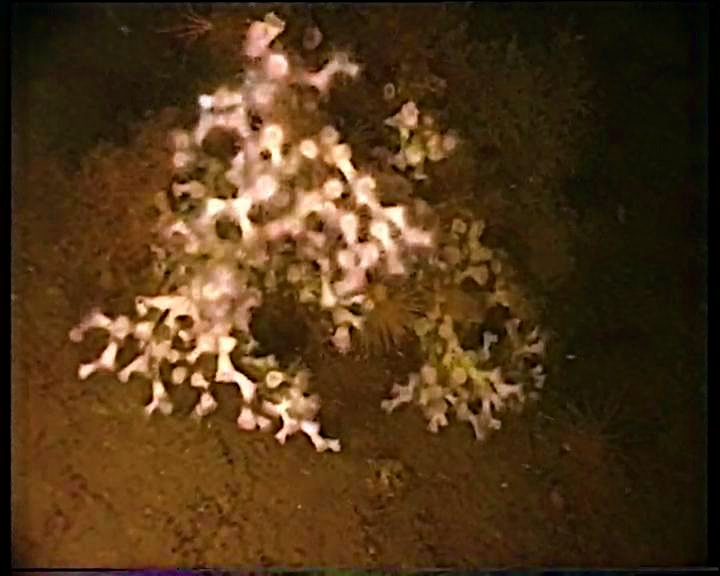

Supplement: Supplementary material 1 — Dataset of underwater images of Desmophyllum pertusum [file bdj-09-e60548-s001.zip › images_new/000203 TMBL-ROV 2000 Sa╠êckenrevet Tape 56_frame_51025.jpg]

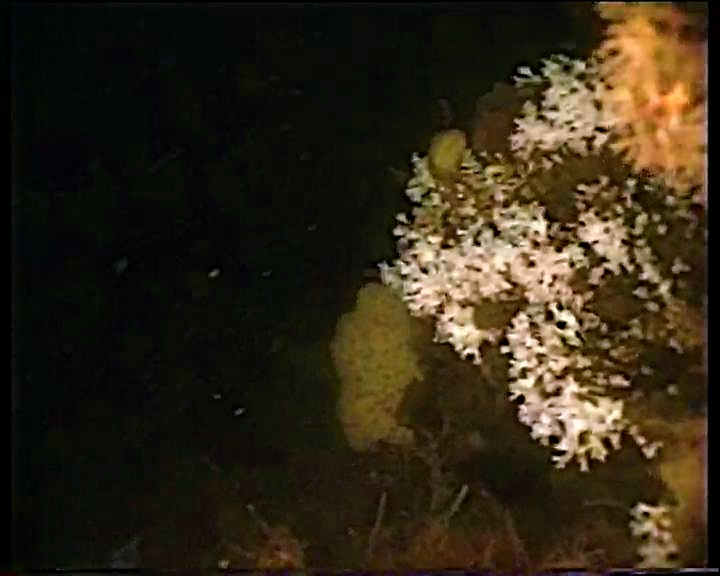

Supplement: Supplementary material 1 — Dataset of underwater images of Desmophyllum pertusum [file bdj-09-e60548-s001.zip › images_new/990506 TMBL-ROV 1999 Revet Sa╠êcken 2 Tape 42_frame_13612.jpg]

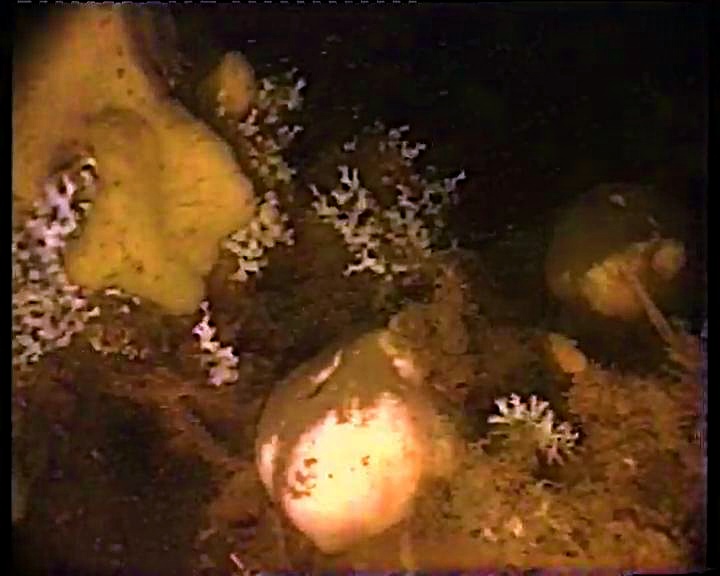

Supplement: Supplementary material 1 — Dataset of underwater images of Desmophyllum pertusum [file bdj-09-e60548-s001.zip › images_new/000203 TMBL-ROV 2000 Sa╠êcken revet EJ numrerade band_frame_27000.jpg]

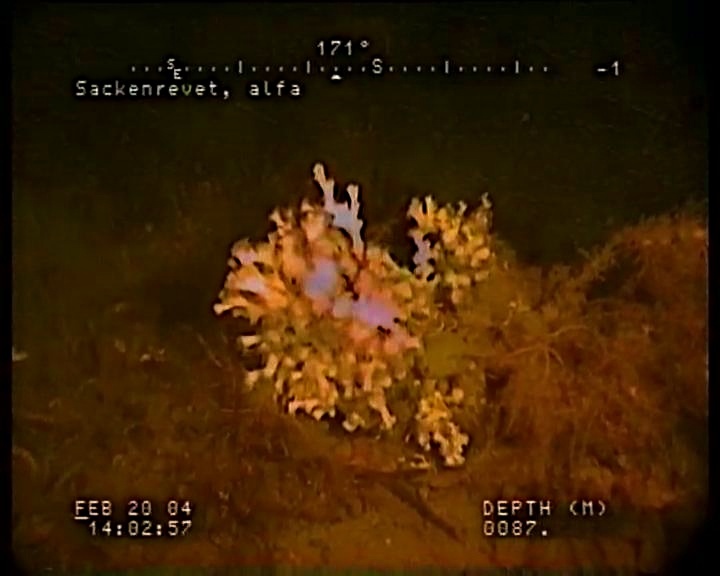

Supplement: Supplementary material 1 — Dataset of underwater images of Desmophyllum pertusum [file bdj-09-e60548-s001.zip › images_new/040220 TMBL-ROV 2004 Sa╠êckenrevet alfa_frame_39775.jpg]

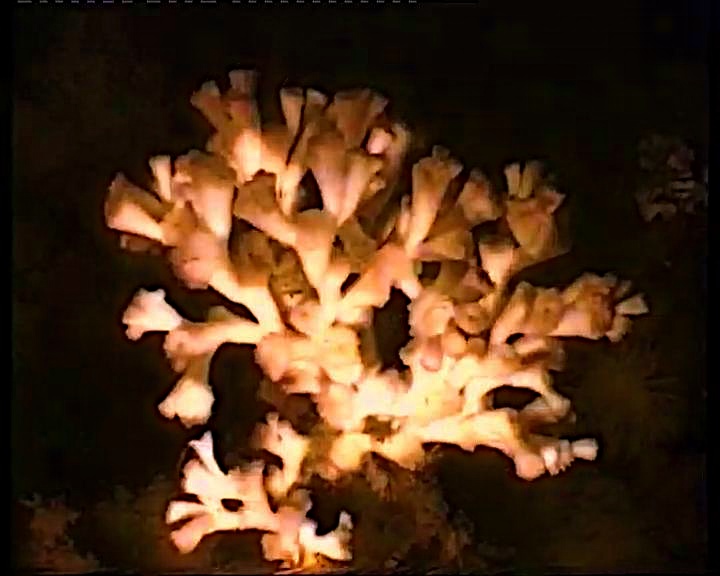

Supplement: Supplementary material 1 — Dataset of underwater images of Desmophyllum pertusum [file bdj-09-e60548-s001.zip › images_new/000203 TMBL-ROV 2000 Sa╠êcken revet EJ numrerade band_frame_40500.jpg]

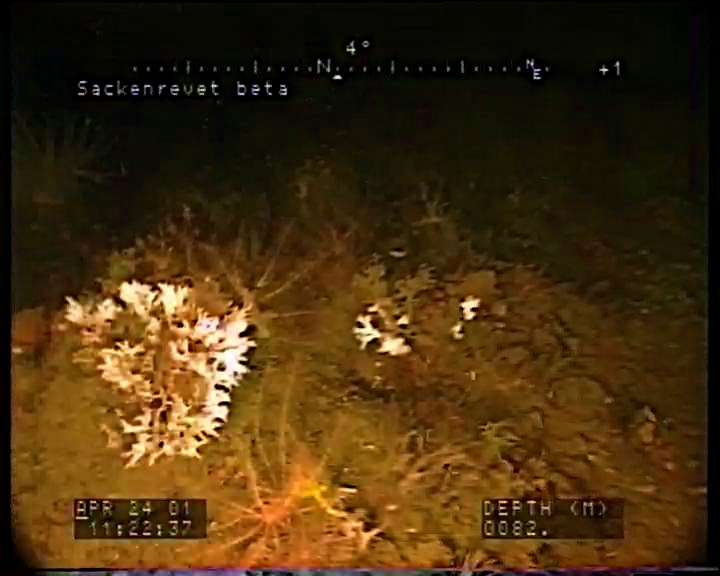

Supplement: Supplementary material 1 — Dataset of underwater images of Desmophyllum pertusum [file bdj-09-e60548-s001.zip › images_new/010424 Sa╠êckenrevet beta Tape 74_frame_32250.jpg]

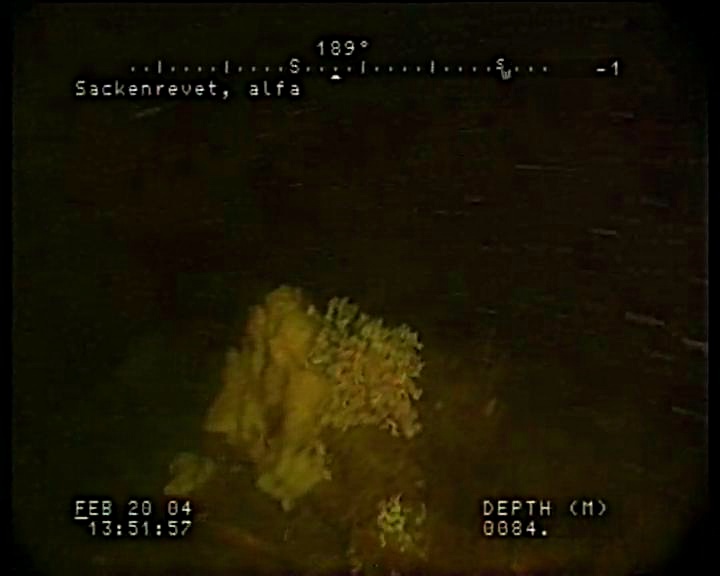

Supplement: Supplementary material 1 — Dataset of underwater images of Desmophyllum pertusum [file bdj-09-e60548-s001.zip › images_new/040220 TMBL-ROV 2004 Sa╠êckenrevet alfa_frame_23275.jpg]

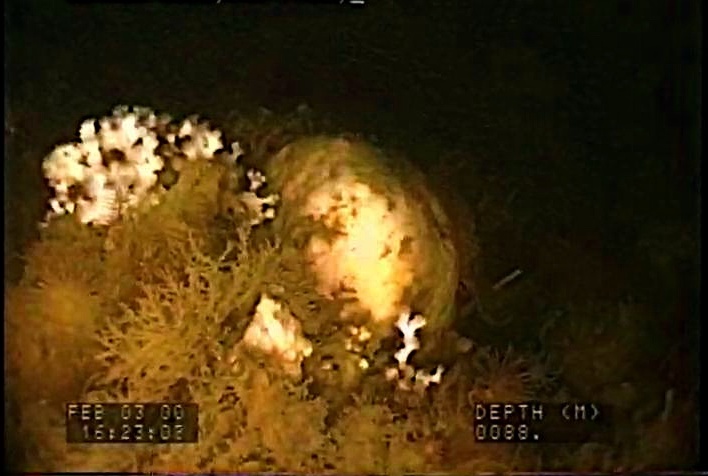

Supplement: Supplementary material 1 — Dataset of underwater images of Desmophyllum pertusum [file bdj-09-e60548-s001.zip › images_new/000203 TMBL-ROV 2000 Sa╠êckenrevet Tape 56_frame_110275.jpg]

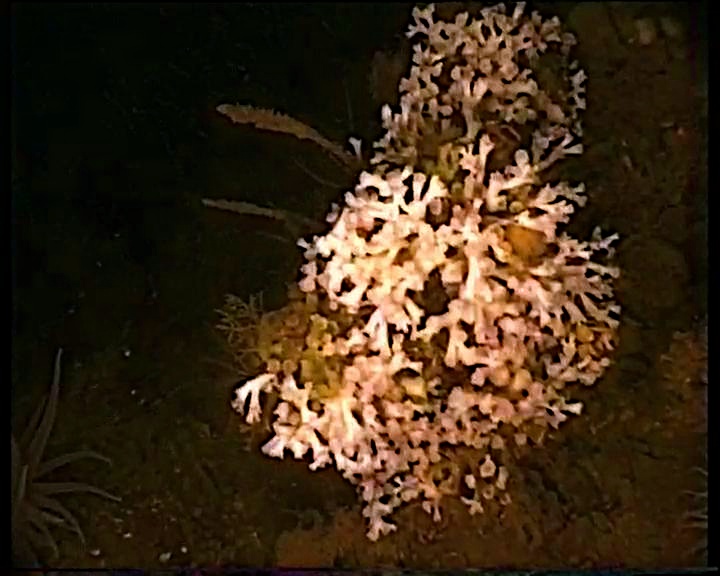

Supplement: Supplementary material 1 — Dataset of underwater images of Desmophyllum pertusum [file bdj-09-e60548-s001.zip › images_new/000114 TMBL-ROV 2000 Sa╠êckenrevet Tape 55_frame_190525.jpg]

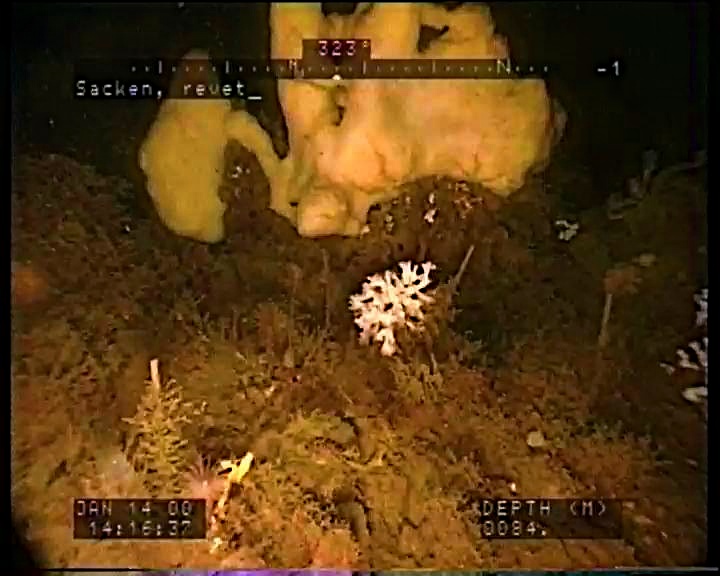

Supplement: Supplementary material 1 — Dataset of underwater images of Desmophyllum pertusum [file bdj-09-e60548-s001.zip › images_new/000114 TMBL-ROV 2000 Sa╠êckenrevet Tape 55_frame_149250.jpg]

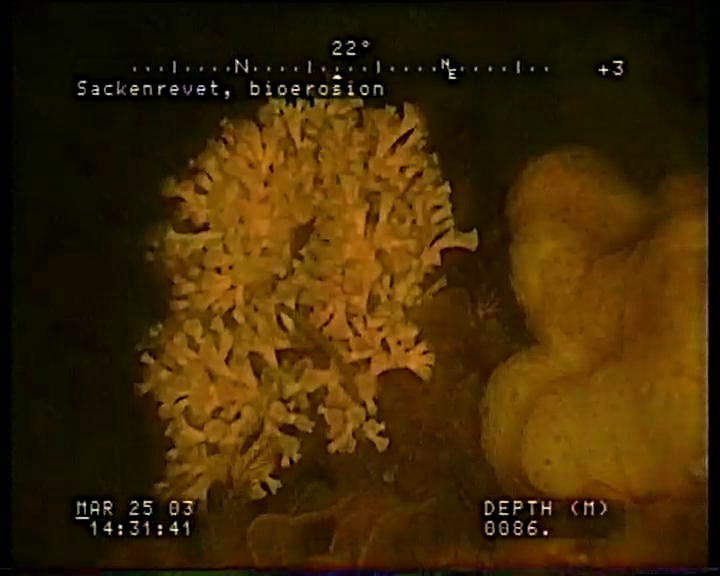

Supplement: Supplementary material 1 — Dataset of underwater images of Desmophyllum pertusum [file bdj-09-e60548-s001.zip › images_new/030325 TMBL-ROV 2003 Sa╠êckenrevet bioerosion_frame_145525.jpg]

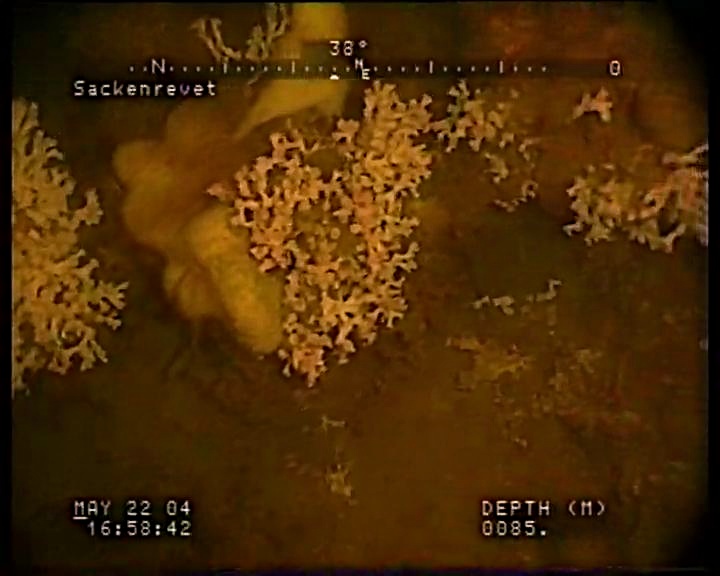

Supplement: Supplementary material 1 — Dataset of underwater images of Desmophyllum pertusum [file bdj-09-e60548-s001.zip › images_new/040522 TMBL-ROV 2004 Sa╠êckenrevet_frame_61525.jpg]

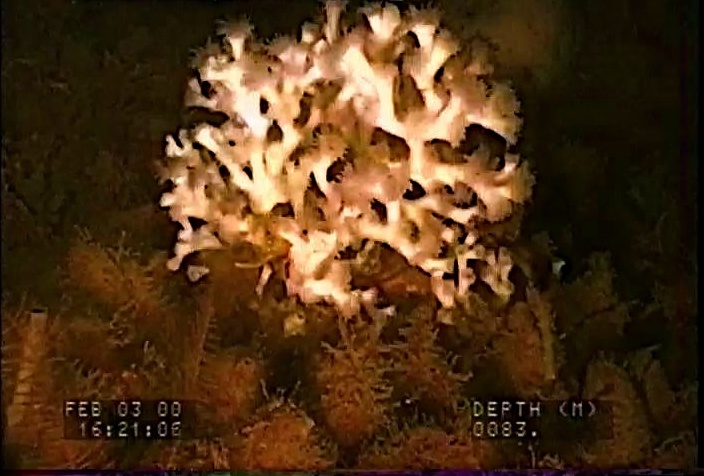

Supplement: Supplementary material 1 — Dataset of underwater images of Desmophyllum pertusum [file bdj-09-e60548-s001.zip › images_new/000203 TMBL-ROV 2000 Sa╠êckenrevet Tape 56_frame_107250.jpg]

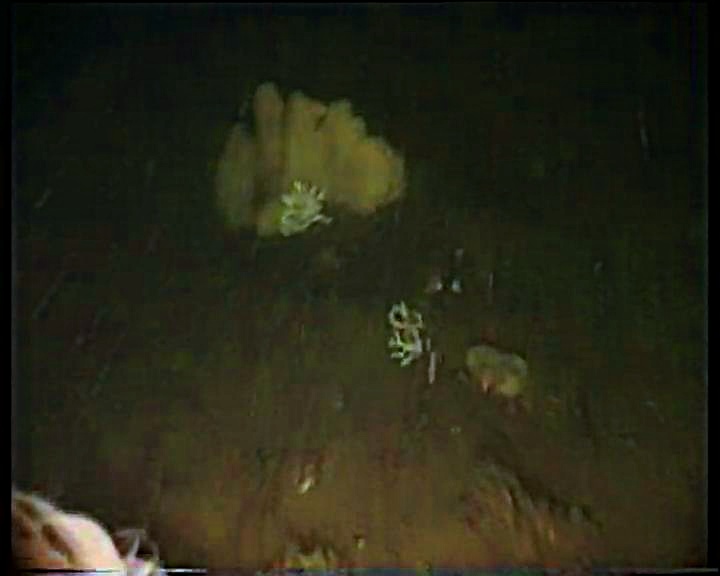

Supplement: Supplementary material 1 — Dataset of underwater images of Desmophyllum pertusum [file bdj-09-e60548-s001.zip › images_new/990506 TMBL-ROV 1999 Revet Sa╠êcken 2 Tape 42_frame_21925.jpg]

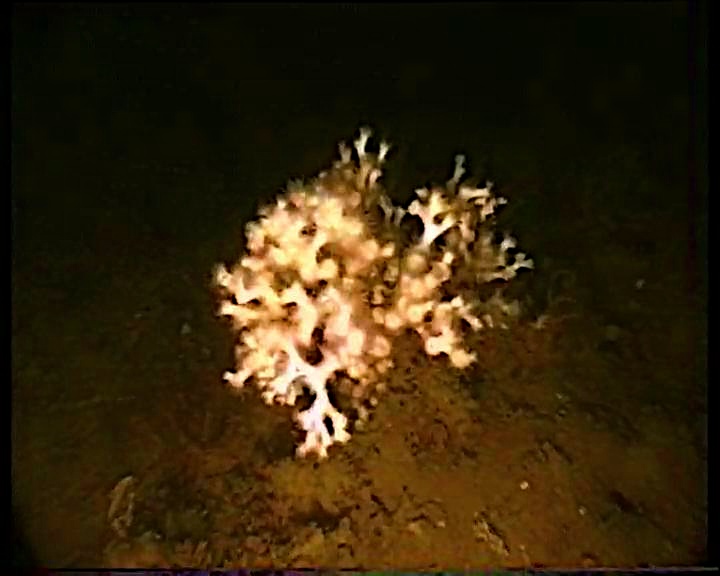

Supplement: Supplementary material 1 — Dataset of underwater images of Desmophyllum pertusum [file bdj-09-e60548-s001.zip › images_new/000203 TMBL-ROV 2000 Sa╠êckenrevet Tape 56_frame_75025.jpg]

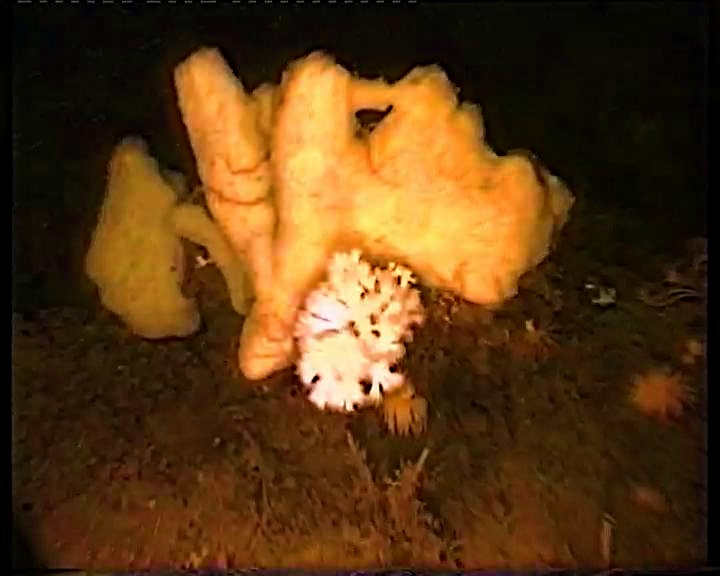

Supplement: Supplementary material 1 — Dataset of underwater images of Desmophyllum pertusum [file bdj-09-e60548-s001.zip › images_new/000114 TMBL-ROV 2000 Sa╠êckenrevet EJ numrerade band_frame_17250.jpg]

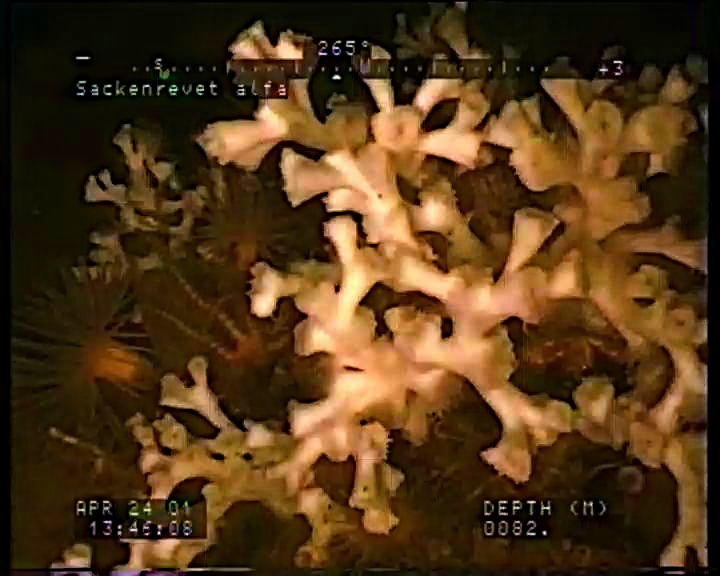

Supplement: Supplementary material 1 — Dataset of underwater images of Desmophyllum pertusum [file bdj-09-e60548-s001.zip › images_new/010424 Sa╠êckenrevet alfa Tape 74_frame_48750.jpg]

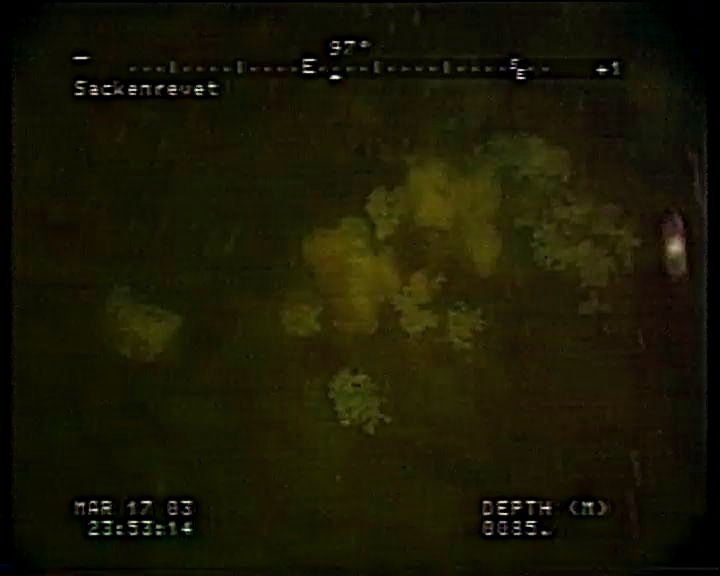

Supplement: Supplementary material 1 — Dataset of underwater images of Desmophyllum pertusum [file bdj-09-e60548-s001.zip › images_new/030317-18 TMBL-ROV 2003 Sa╠êckenrevet_frame_78000.jpg]

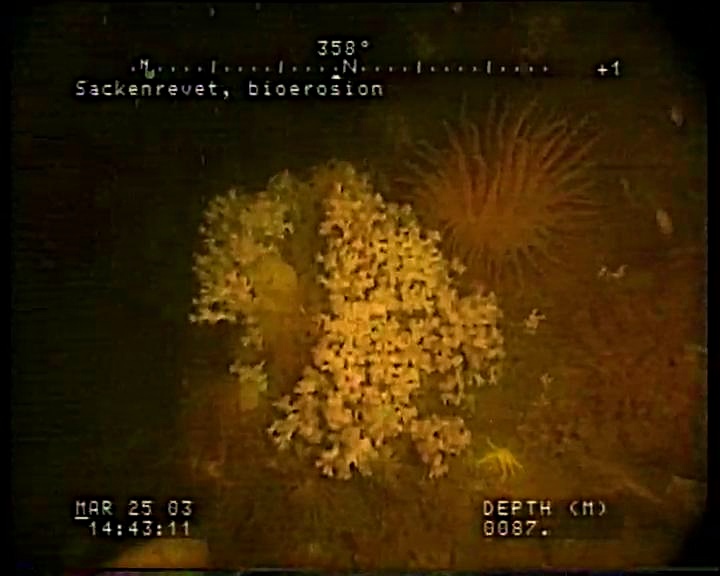

Supplement: Supplementary material 1 — Dataset of underwater images of Desmophyllum pertusum [file bdj-09-e60548-s001.zip › images_new/030325 TMBL-ROV 2003 Sa╠êckenrevet bioerosion_frame_162775.jpg]

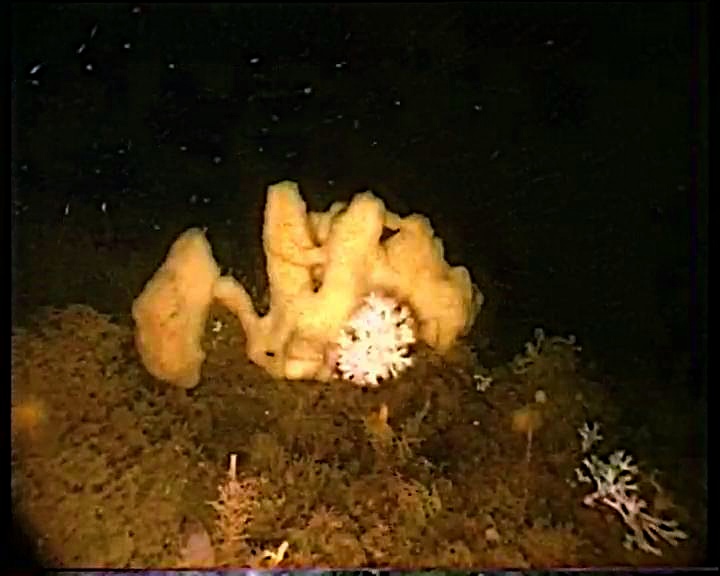

Supplement: Supplementary material 1 — Dataset of underwater images of Desmophyllum pertusum [file bdj-09-e60548-s001.zip › images_new/000114 TMBL-ROV 2000 Sa╠êckenrevet Tape 55_frame_132025.jpg]

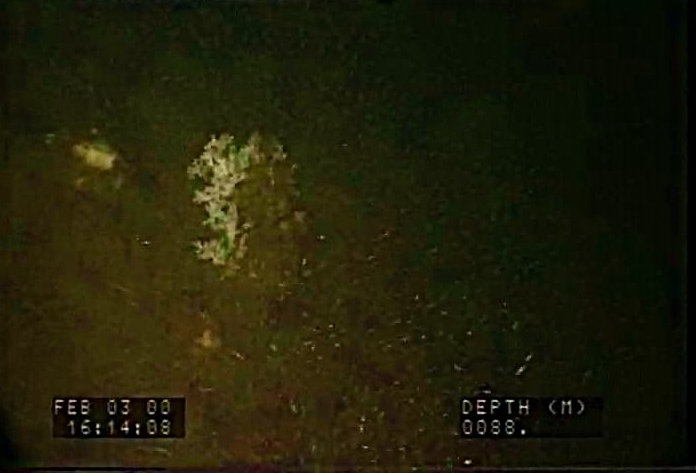

Supplement: Supplementary material 1 — Dataset of underwater images of Desmophyllum pertusum [file bdj-09-e60548-s001.zip › images_new/000203 TMBL-ROV 2000 Sa╠êckenrevet Tape 56_frame_96800.jpg]

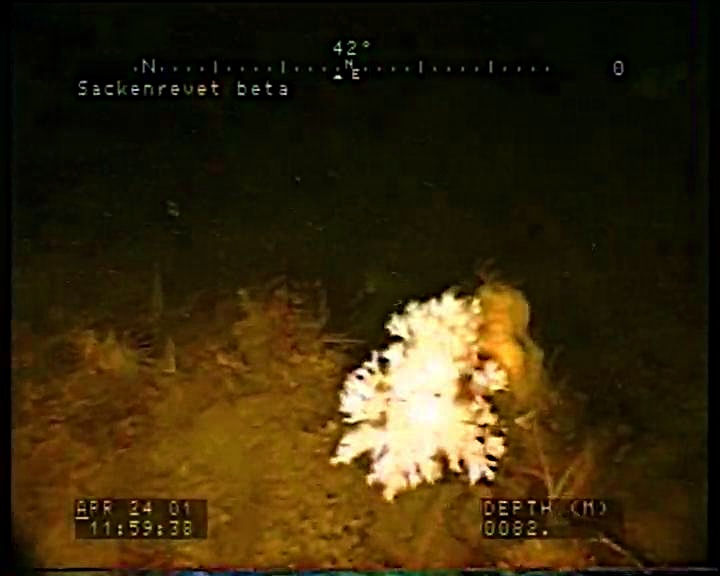

Supplement: Supplementary material 1 — Dataset of underwater images of Desmophyllum pertusum [file bdj-09-e60548-s001.zip › images_new/010424 Sa╠êckenrevet beta Tape 74_frame_87775.jpg]

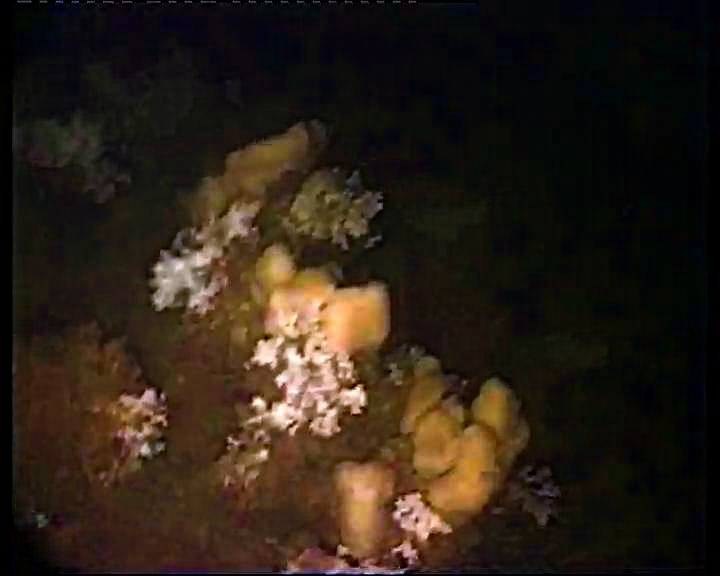

Supplement: Supplementary material 1 — Dataset of underwater images of Desmophyllum pertusum [file bdj-09-e60548-s001.zip › images_new/000203 TMBL-ROV 2000 Sa╠êcken revet EJ numrerade band_frame_51750.jpg]

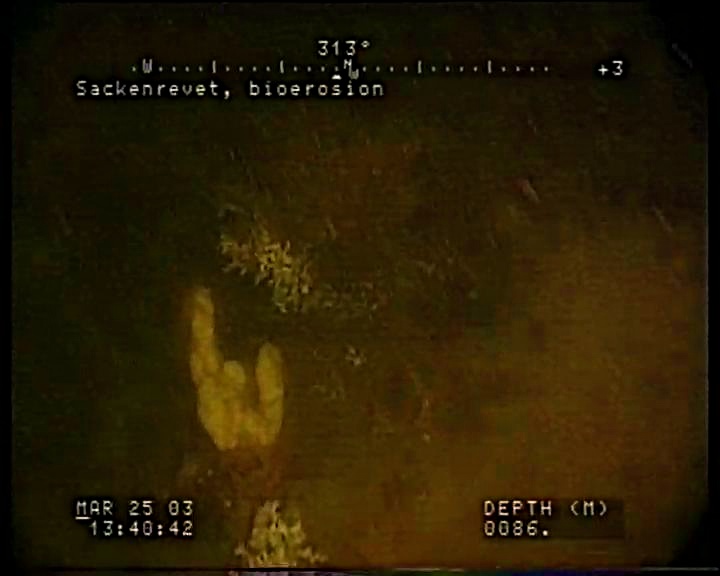

Supplement: Supplementary material 1 — Dataset of underwater images of Desmophyllum pertusum [file bdj-09-e60548-s001.zip › images_new/030325 TMBL-ROV 2003 Sa╠êckenrevet bioerosion_frame_69037.jpg]

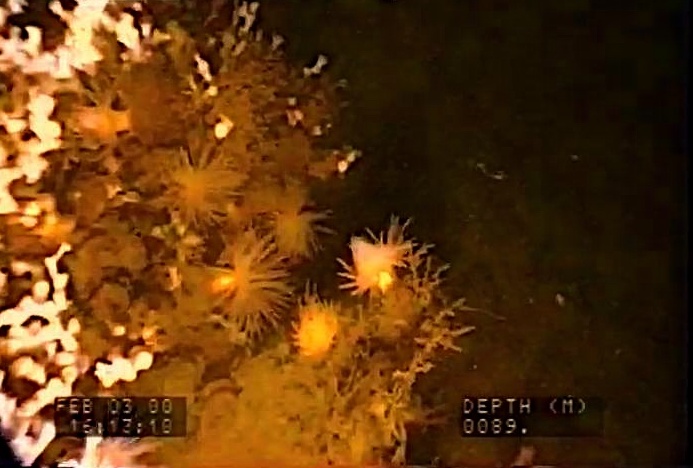

Supplement: Supplementary material 1 — Dataset of underwater images of Desmophyllum pertusum [file bdj-09-e60548-s001.zip › images_new/000203 TMBL-ROV 2000 Sa╠êcken revet EJ numrerade band_frame_59250.jpg]

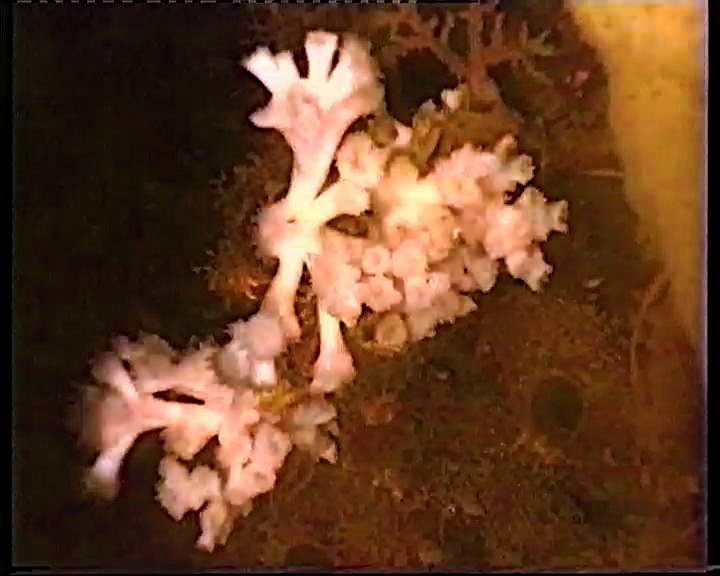

Supplement: Supplementary material 1 — Dataset of underwater images of Desmophyllum pertusum [file bdj-09-e60548-s001.zip › images_new/000114 TMBL-ROV 2000 Sa╠êckenrevet EJ numrerade band_frame_49500.jpg]

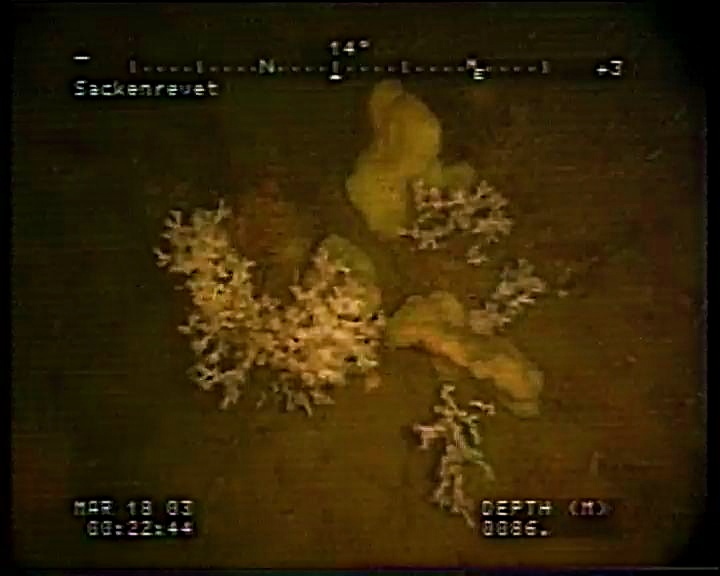

Supplement: Supplementary material 1 — Dataset of underwater images of Desmophyllum pertusum [file bdj-09-e60548-s001.zip › images_new/030317-18 TMBL-ROV 2003 Sa╠êckenrevet_frame_122250.jpg]

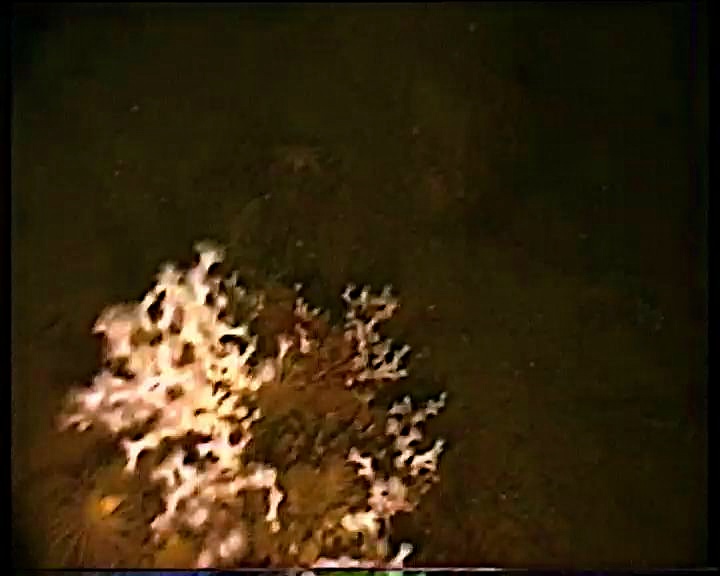

Supplement: Supplementary material 1 — Dataset of underwater images of Desmophyllum pertusum [file bdj-09-e60548-s001.zip › images_new/000203 TMBL-ROV 2000 Sa╠êckenrevet Tape 56_frame_38250.jpg]

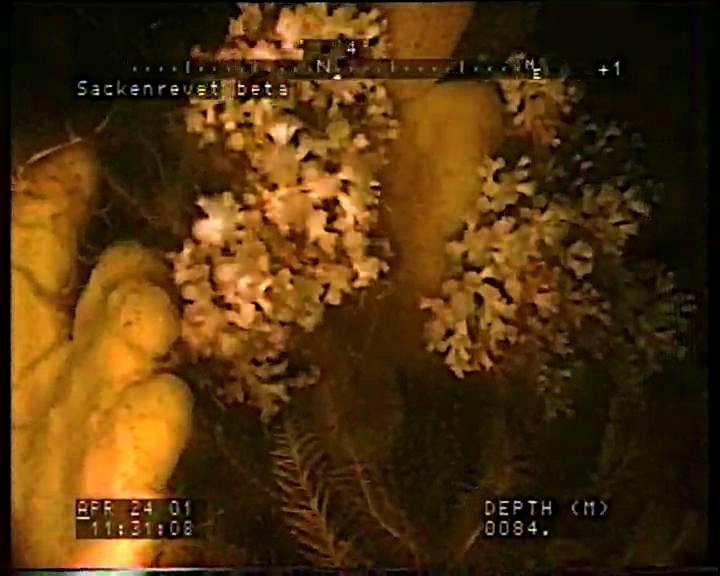

Supplement: Supplementary material 1 — Dataset of underwater images of Desmophyllum pertusum [file bdj-09-e60548-s001.zip › images_new/010424 Sa╠êckenrevet beta Tape 74_frame_45025.jpg]

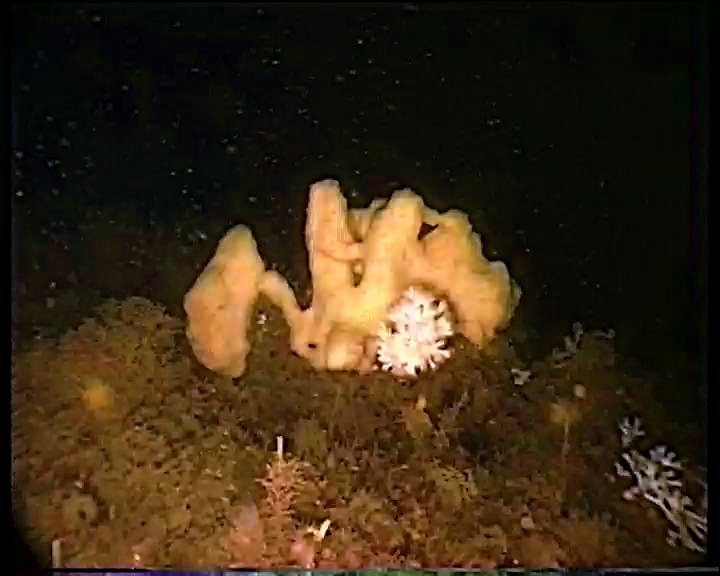

Supplement: Supplementary material 1 — Dataset of underwater images of Desmophyllum pertusum [file bdj-09-e60548-s001.zip › images_new/000114 TMBL-ROV 2000 Sa╠êckenrevet Tape 55_frame_133500.jpg]

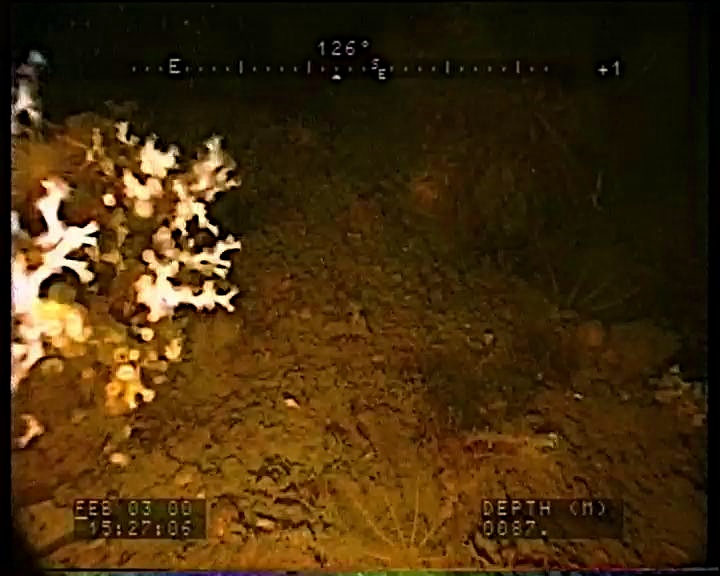

Supplement: Supplementary material 1 — Dataset of underwater images of Desmophyllum pertusum [file bdj-09-e60548-s001.zip › images_new/000203 TMBL-ROV 2000 Sa╠êckenrevet Tape 56_frame_26250.jpg]

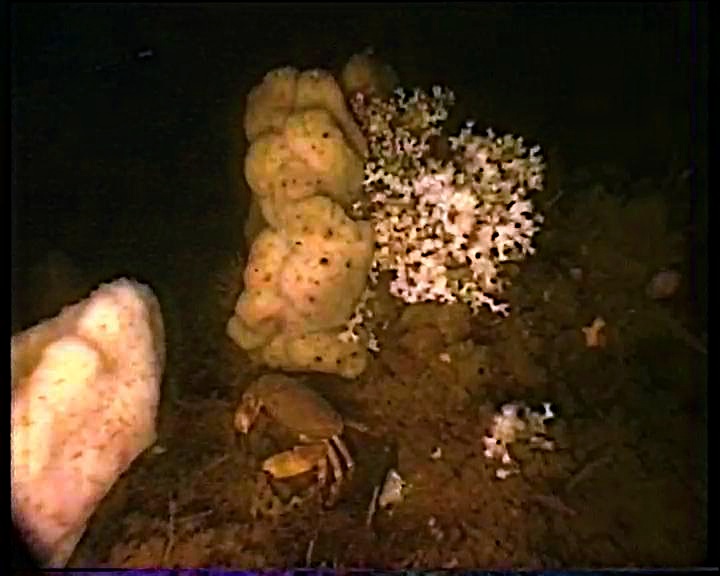

Supplement: Supplementary material 1 — Dataset of underwater images of Desmophyllum pertusum [file bdj-09-e60548-s001.zip › images_new/000203 TMBL-ROV 2000 Sa╠êckenrevet Tape 56_frame_82525.jpg]

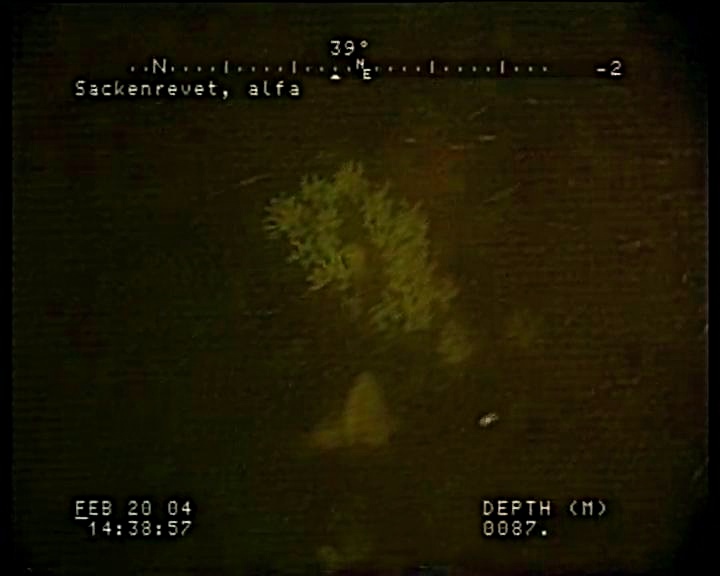

Supplement: Supplementary material 1 — Dataset of underwater images of Desmophyllum pertusum [file bdj-09-e60548-s001.zip › images_new/040220 TMBL-ROV 2004 Sa╠êckenrevet alfa_frame_93775.jpg]

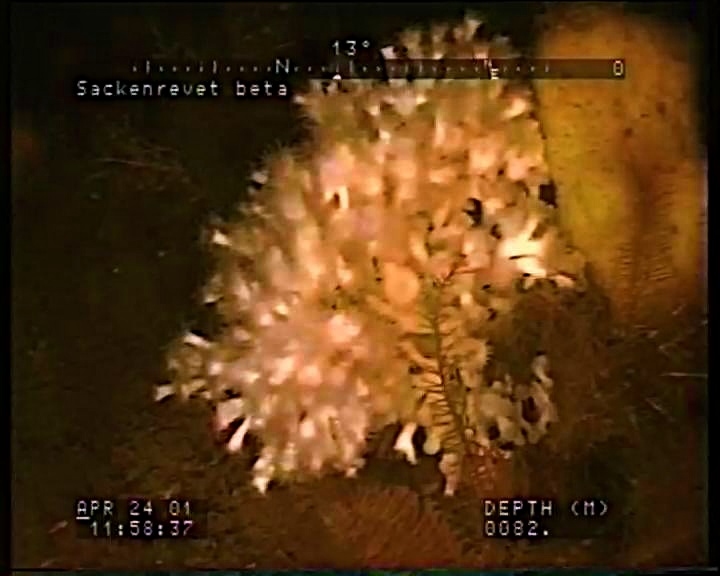

Supplement: Supplementary material 1 — Dataset of underwater images of Desmophyllum pertusum [file bdj-09-e60548-s001.zip › images_new/010424 Sa╠êckenrevet beta Tape 74_frame_86250.jpg]

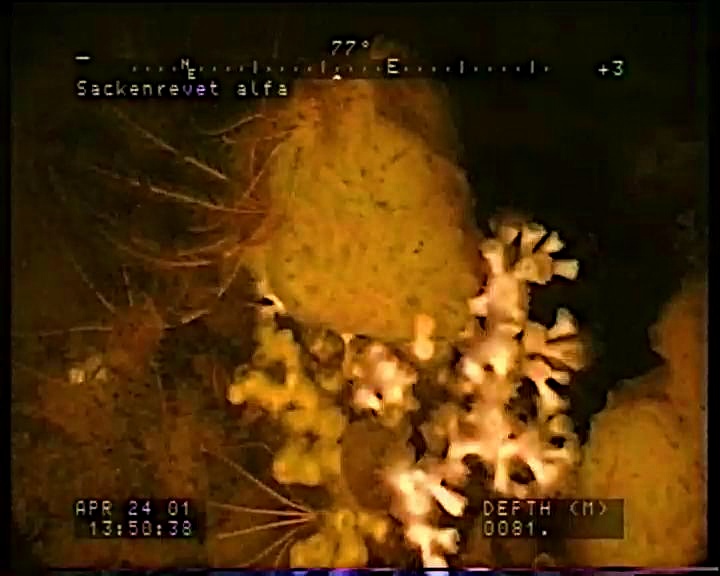

Supplement: Supplementary material 1 — Dataset of underwater images of Desmophyllum pertusum [file bdj-09-e60548-s001.zip › images_new/010424 Sa╠êckenrevet alfa Tape 74_frame_55500.jpg]

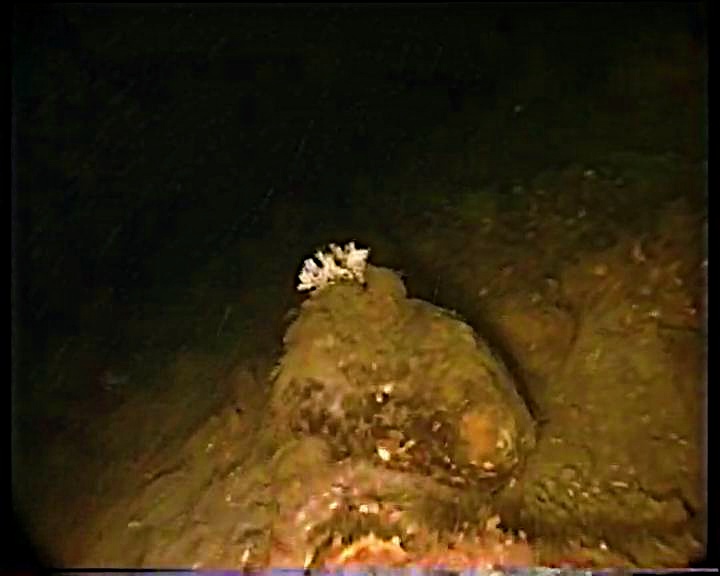

Supplement: Supplementary material 1 — Dataset of underwater images of Desmophyllum pertusum [file bdj-09-e60548-s001.zip › images_new/990506 TMBL-ROV 1999 Revet Sa╠êcken Tape 42_SELECTWS_frame_76725.jpg]

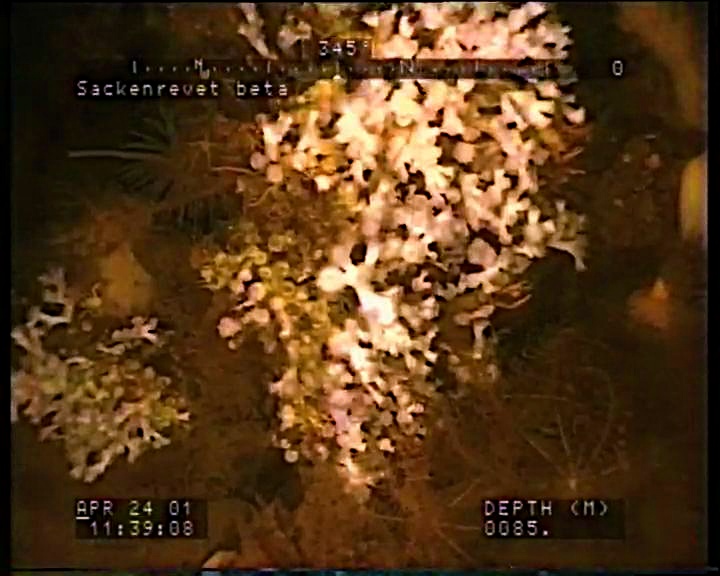

Supplement: Supplementary material 1 — Dataset of underwater images of Desmophyllum pertusum [file bdj-09-e60548-s001.zip › images_new/010424 Sa╠êckenrevet beta Tape 74_frame_57025.jpg]

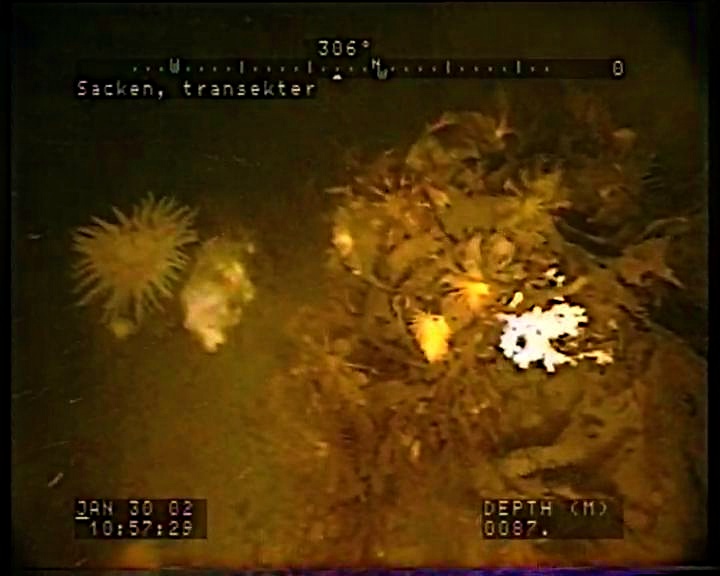

Supplement: Supplementary material 1 — Dataset of underwater images of Desmophyllum pertusum [file bdj-09-e60548-s001.zip › images_new/020130 TMBL-ROV 2002 Sa╠êcken transekt 1_frame_13525.jpg]

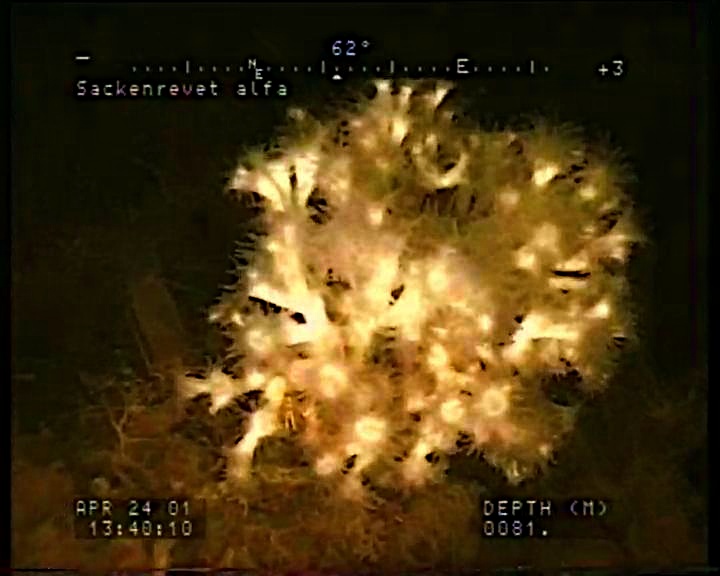

Supplement: Supplementary material 1 — Dataset of underwater images of Desmophyllum pertusum [file bdj-09-e60548-s001.zip › images_new/010424 Sa╠êckenrevet alfa Tape 74_frame_39800.jpg]

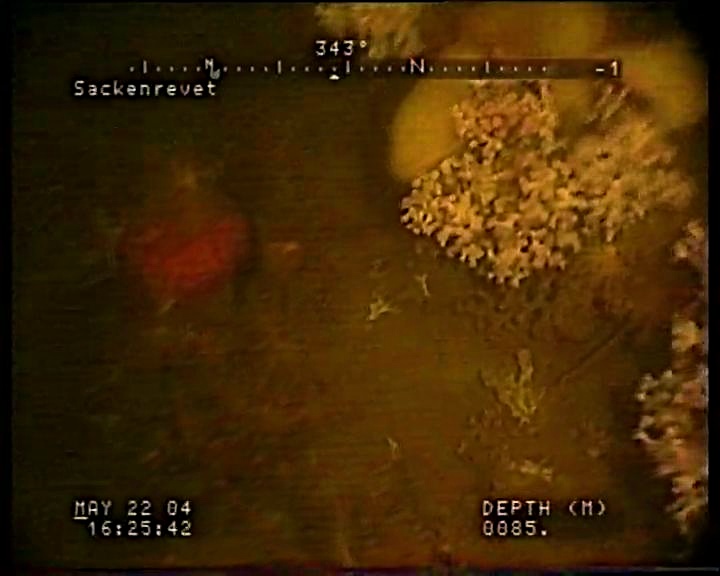

Supplement: Supplementary material 1 — Dataset of underwater images of Desmophyllum pertusum [file bdj-09-e60548-s001.zip › images_new/040522 TMBL-ROV 2004 Sa╠êckenrevet_frame_17275.jpg]

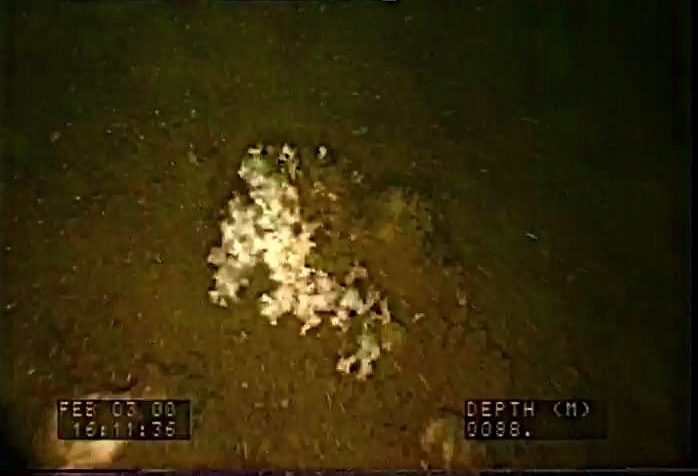

Supplement: Supplementary material 1 — Dataset of underwater images of Desmophyllum pertusum [file bdj-09-e60548-s001.zip › images_new/000203 TMBL-ROV 2000 Sa╠êckenrevet Tape 56_frame_93000.jpg]

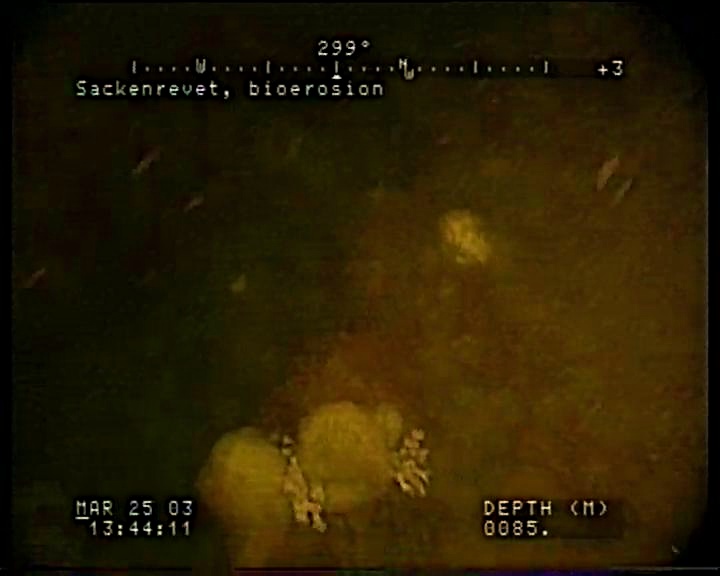

Supplement: Supplementary material 1 — Dataset of underwater images of Desmophyllum pertusum [file bdj-09-e60548-s001.zip › images_new/030325 TMBL-ROV 2003 Sa╠êckenrevet bioerosion_frame_74275.jpg]

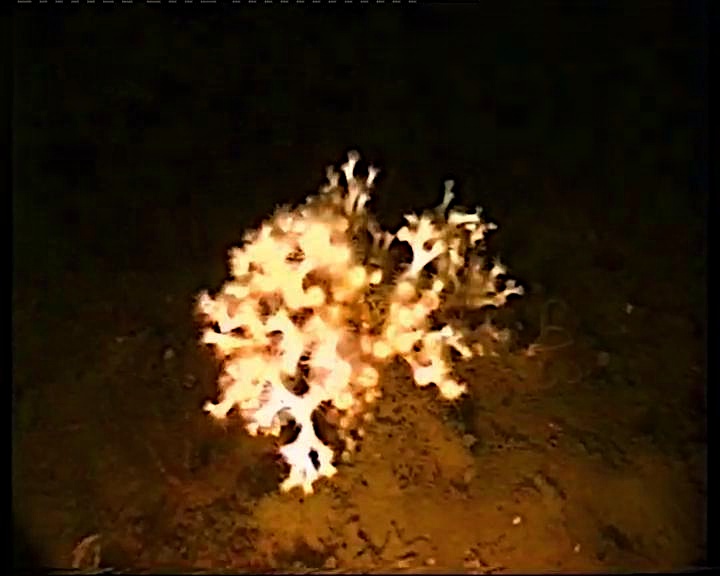

Supplement: Supplementary material 1 — Dataset of underwater images of Desmophyllum pertusum [file bdj-09-e60548-s001.zip › images_new/000203 TMBL-ROV 2000 Sa╠êcken revet EJ numrerade band_frame_38250.jpg]

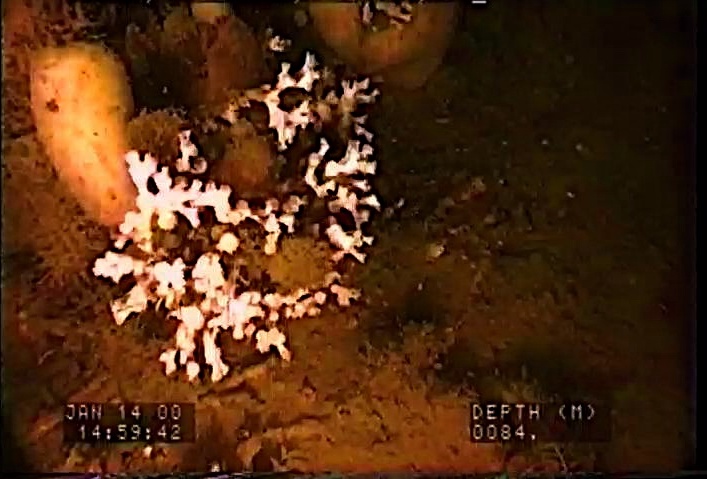

Supplement: Supplementary material 1 — Dataset of underwater images of Desmophyllum pertusum [file bdj-09-e60548-s001.zip › images_new/000114 TMBL-ROV 2000 Sa╠êckenrevet EJ numrerade band_frame_51000.jpg]

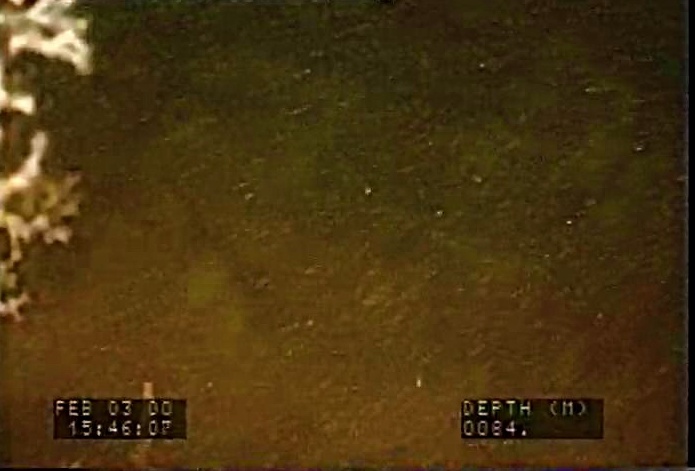

Supplement: Supplementary material 1 — Dataset of underwater images of Desmophyllum pertusum [file bdj-09-e60548-s001.zip › images_new/000203 TMBL-ROV 2000 Sa╠êckenrevet Tape 56_frame_54775.jpg]

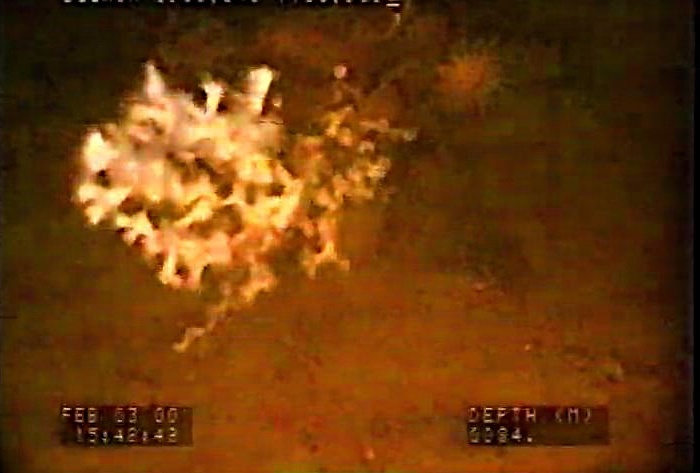

Supplement: Supplementary material 1 — Dataset of underwater images of Desmophyllum pertusum [file bdj-09-e60548-s001.zip › images_new/000203 TMBL-ROV 2000 Sa╠êcken revet EJ numrerade band_frame_26287.jpg]

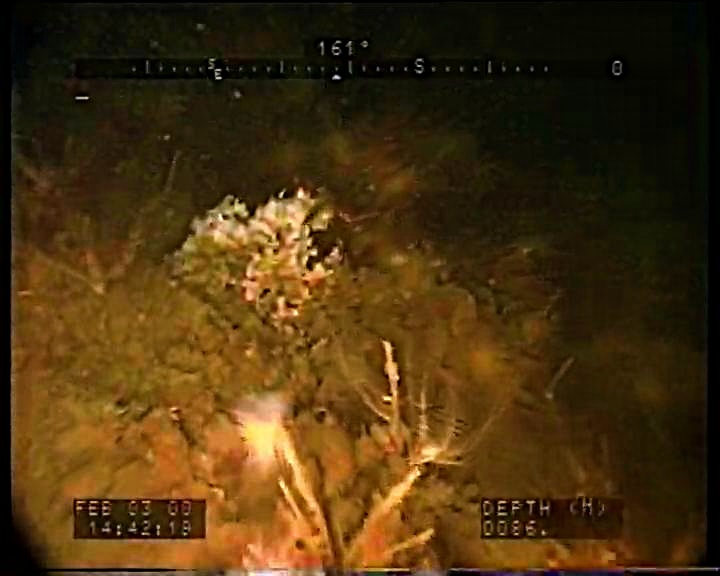

Supplement: Supplementary material 1 — Dataset of underwater images of Desmophyllum pertusum [file bdj-09-e60548-s001.zip › images_new/000203 TMBL-ROV 2000 sa╠êcken Tape 56_frame_48150.jpg]

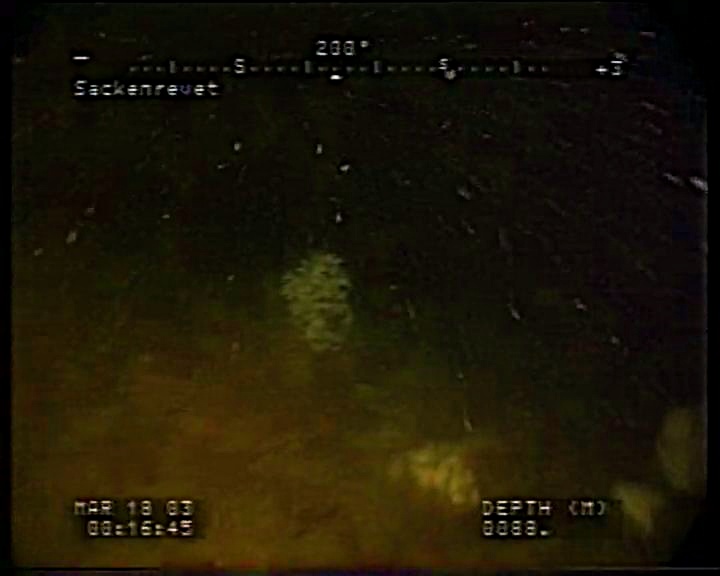

Supplement: Supplementary material 1 — Dataset of underwater images of Desmophyllum pertusum [file bdj-09-e60548-s001.zip › images_new/030317-18 TMBL-ROV 2003 Sa╠êckenrevet_frame_113275.jpg]

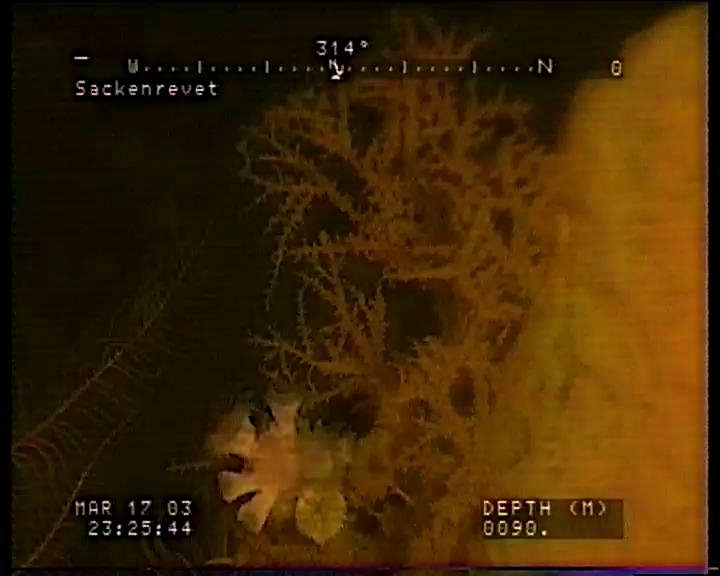

Supplement: Supplementary material 1 — Dataset of underwater images of Desmophyllum pertusum [file bdj-09-e60548-s001.zip › images_new/030317-18 TMBL-ROV 2003 Sa╠êckenrevet_frame_36750.jpg]

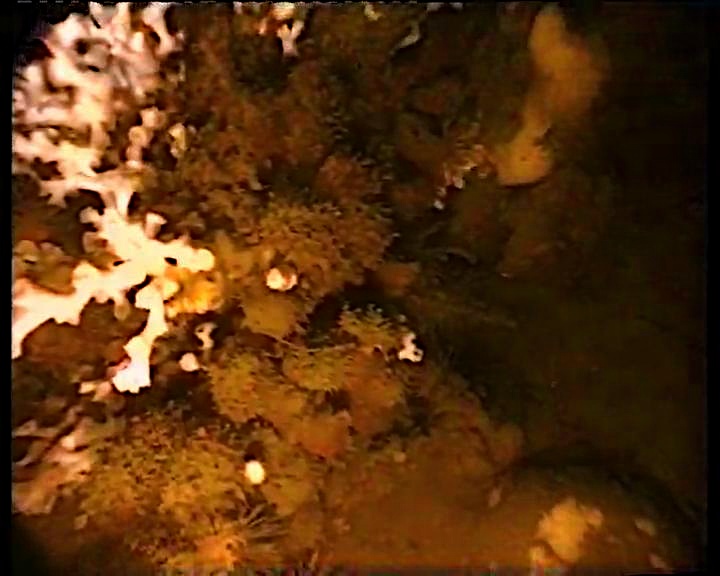

Supplement: Supplementary material 1 — Dataset of underwater images of Desmophyllum pertusum [file bdj-09-e60548-s001.zip › images_new/000203 TMBL-ROV 2000 Sa╠êcken revet EJ numrerade band_frame_5275.jpg]

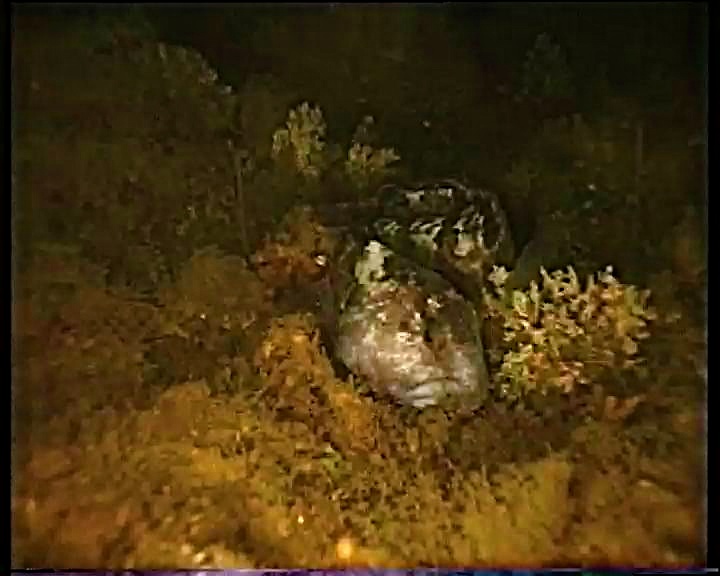

Supplement: Supplementary material 1 — Dataset of underwater images of Desmophyllum pertusum [file bdj-09-e60548-s001.zip › images_new/990506 TMBL-ROV 1999 Revet Sa╠êcken 2 Tape 42_frame_93000.jpg]

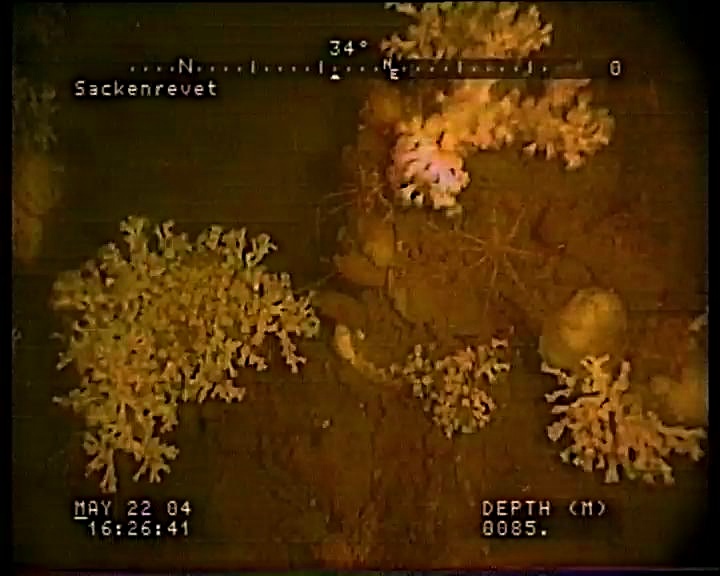

Supplement: Supplementary material 1 — Dataset of underwater images of Desmophyllum pertusum [file bdj-09-e60548-s001.zip › images_new/040522 TMBL-ROV 2004 Sa╠êckenrevet_frame_18750.jpg]

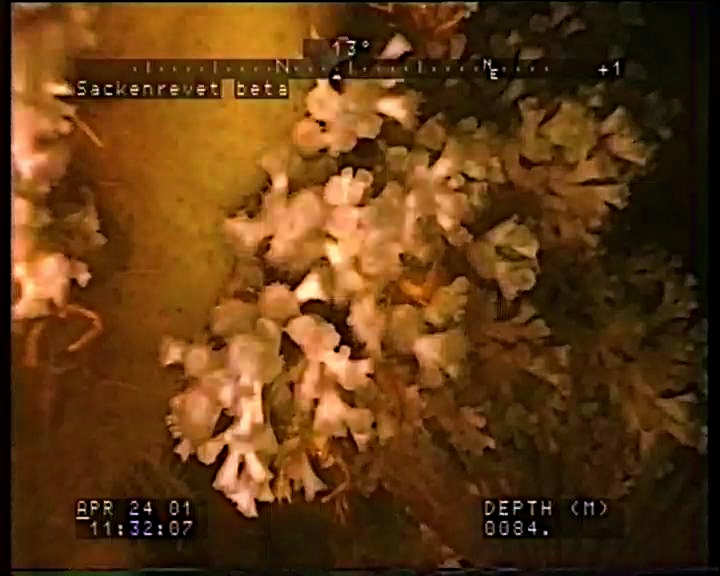

Supplement: Supplementary material 1 — Dataset of underwater images of Desmophyllum pertusum [file bdj-09-e60548-s001.zip › images_new/010424 Sa╠êckenrevet beta Tape 74_frame_46500.jpg]

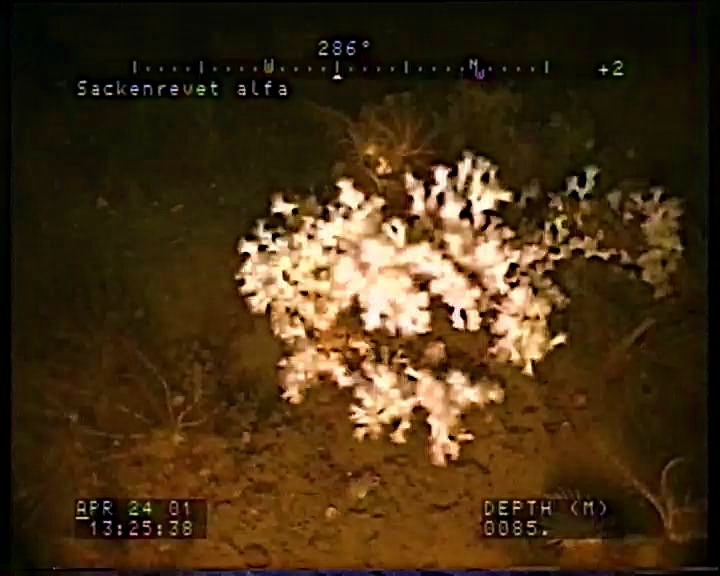

Supplement: Supplementary material 1 — Dataset of underwater images of Desmophyllum pertusum [file bdj-09-e60548-s001.zip › images_new/010424 Sa╠êckenrevet alfa Tape 74_frame_18000.jpg]

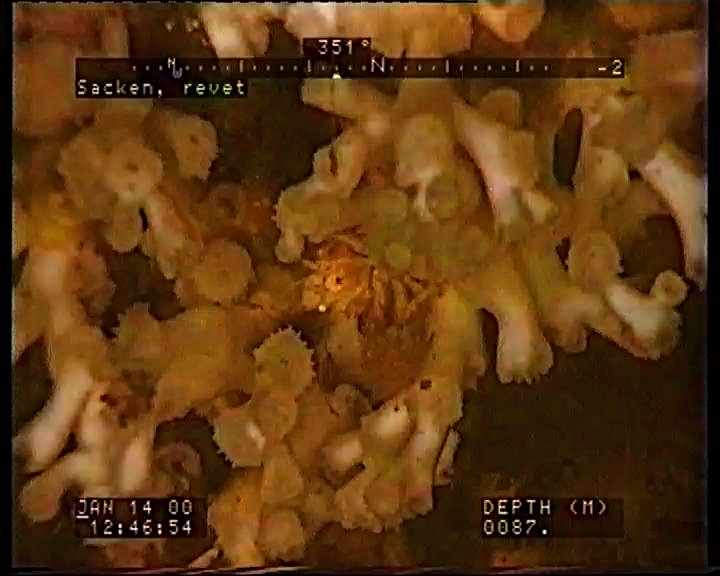

Supplement: Supplementary material 1 — Dataset of underwater images of Desmophyllum pertusum [file bdj-09-e60548-s001.zip › images_new/000114 TMBL-ROV 2000 Sa╠êckenrevet EJ numrerade band_frame_5250.jpg]

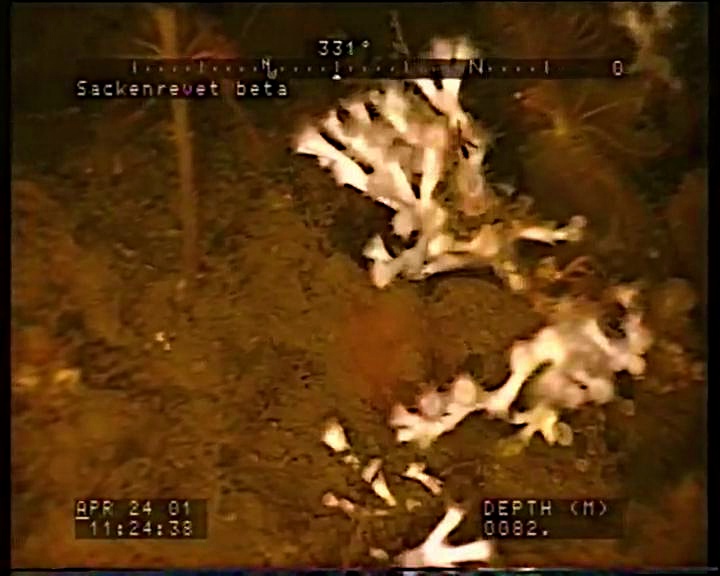

Supplement: Supplementary material 1 — Dataset of underwater images of Desmophyllum pertusum [file bdj-09-e60548-s001.zip › images_new/010424 Sa╠êckenrevet beta Tape 74_frame_35262.jpg]

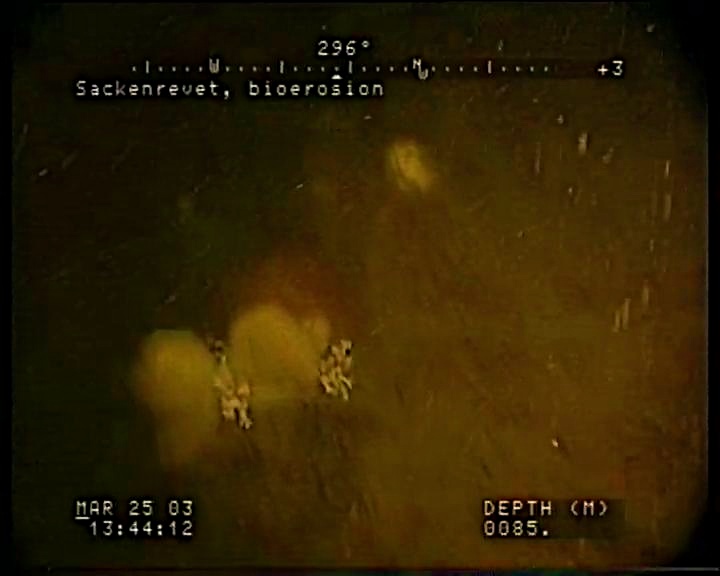

Supplement: Supplementary material 1 — Dataset of underwater images of Desmophyllum pertusum [file bdj-09-e60548-s001.zip › images_new/030325 TMBL-ROV 2003 Sa╠êckenrevet bioerosion_frame_74300.jpg]

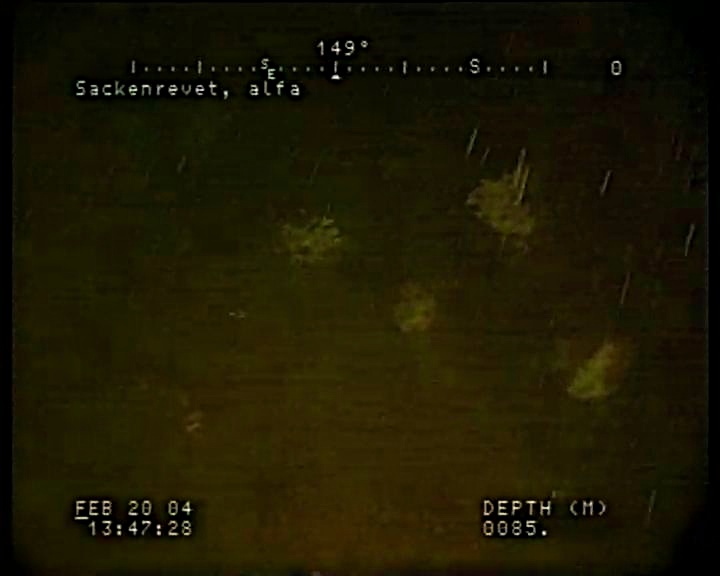

Supplement: Supplementary material 1 — Dataset of underwater images of Desmophyllum pertusum [file bdj-09-e60548-s001.zip › images_new/040220 TMBL-ROV 2004 Sa╠êckenrevet alfa_frame_16550.jpg]

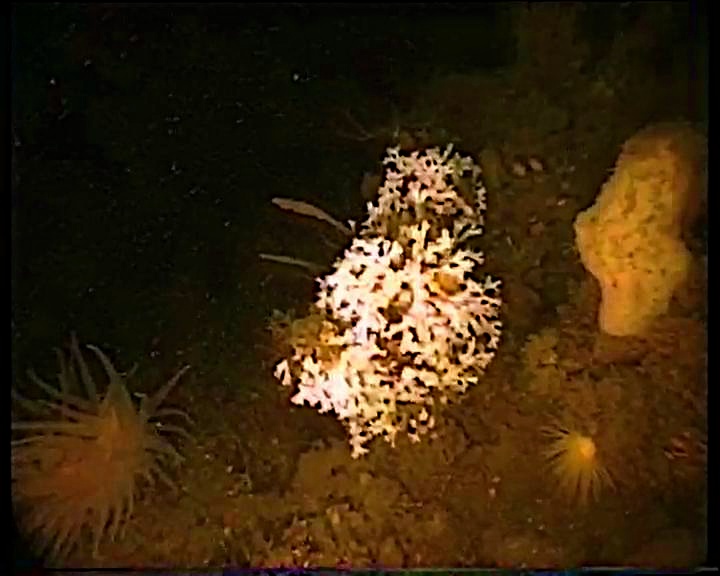

Supplement: Supplementary material 1 — Dataset of underwater images of Desmophyllum pertusum [file bdj-09-e60548-s001.zip › images_new/000114 TMBL-ROV 2000 Sa╠êckenrevet Tape 55_frame_183775.jpg]

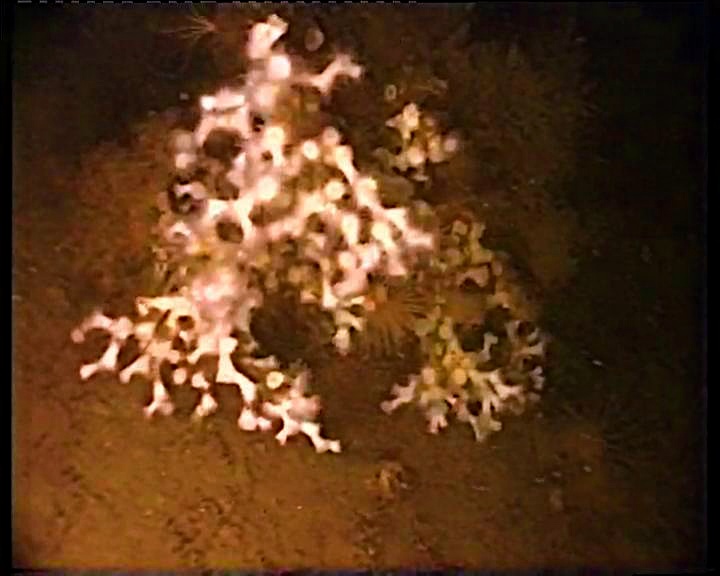

Supplement: Supplementary material 1 — Dataset of underwater images of Desmophyllum pertusum [file bdj-09-e60548-s001.zip › images_new/000203 TMBL-ROV 2000 Sa╠êcken revet EJ numrerade band_frame_23275.jpg]

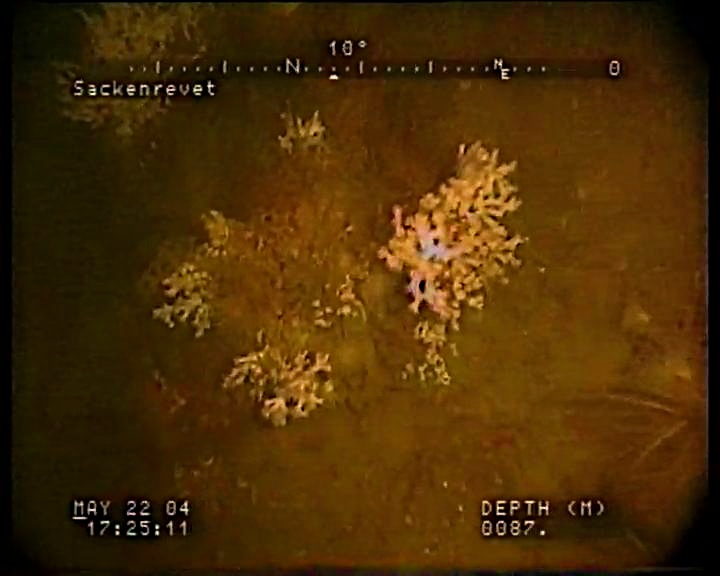

Supplement: Supplementary material 1 — Dataset of underwater images of Desmophyllum pertusum [file bdj-09-e60548-s001.zip › images_new/040522 TMBL-ROV 2004 Sa╠êckenrevet_frame_101250.jpg]

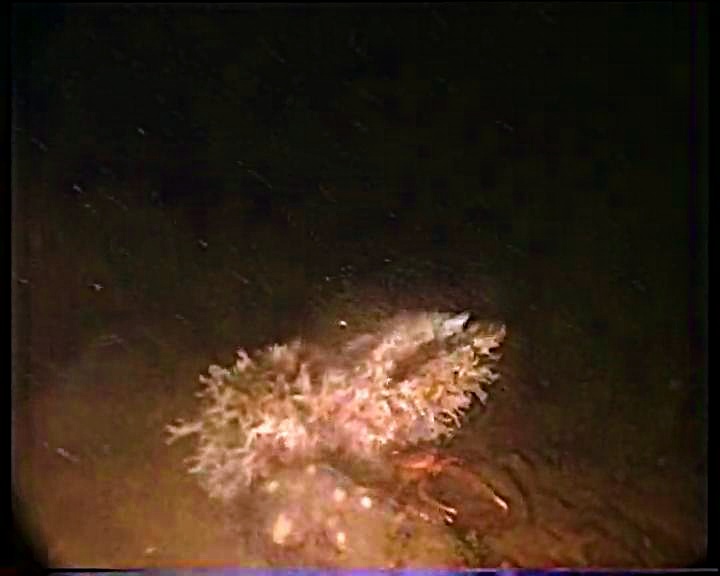

Supplement: Supplementary material 1 — Dataset of underwater images of Desmophyllum pertusum [file bdj-09-e60548-s001.zip › images_new/990506 TMBL-ROV 1999 Revet Sa╠êcken Tape 42_SELECTWS_frame_30775.jpg]

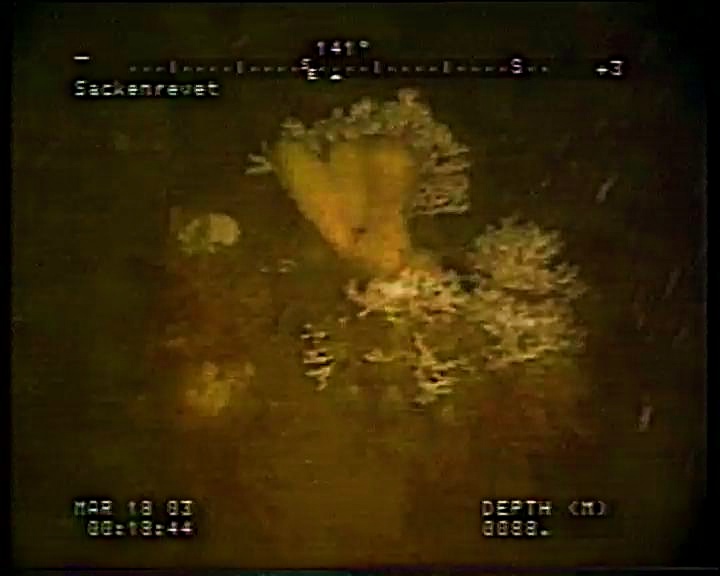

Supplement: Supplementary material 1 — Dataset of underwater images of Desmophyllum pertusum [file bdj-09-e60548-s001.zip › images_new/030317-18 TMBL-ROV 2003 Sa╠êckenrevet_frame_116250.jpg]

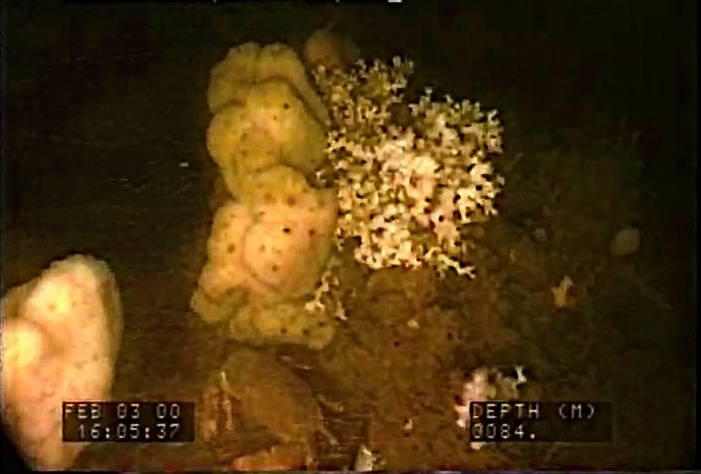

Supplement: Supplementary material 1 — Dataset of underwater images of Desmophyllum pertusum [file bdj-09-e60548-s001.zip › images_new/000203 TMBL-ROV 2000 Sa╠êckenrevet Tape 56_frame_84025.jpg]

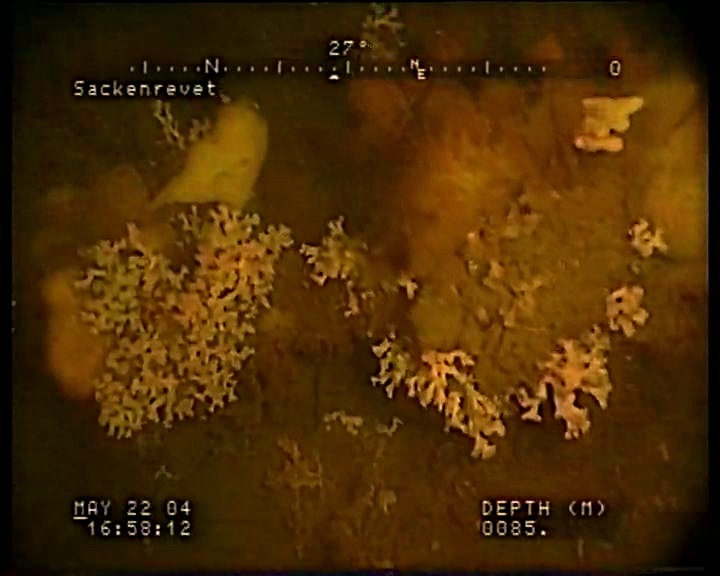

Supplement: Supplementary material 1 — Dataset of underwater images of Desmophyllum pertusum [file bdj-09-e60548-s001.zip › images_new/040522 TMBL-ROV 2004 Sa╠êckenrevet_frame_60775.jpg]

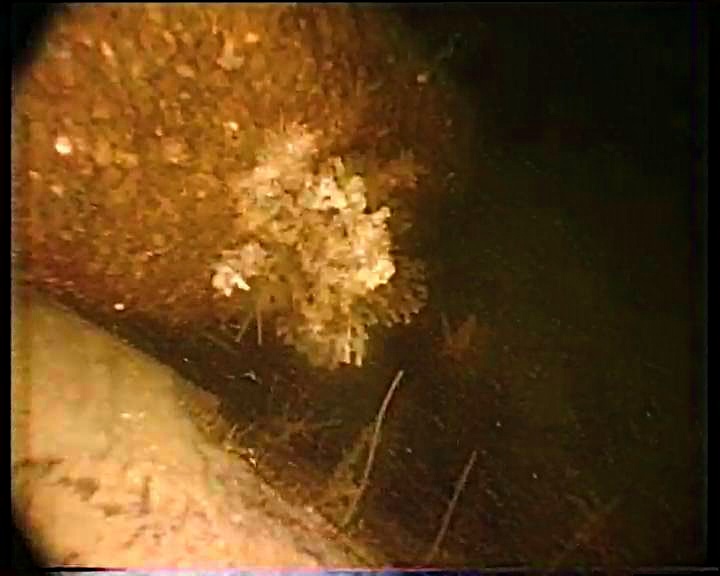

Supplement: Supplementary material 1 — Dataset of underwater images of Desmophyllum pertusum [file bdj-09-e60548-s001.zip › images_new/990813 TMBL-ROV 1999 Storo╠ê-Torso╠ê Utsidan ra╠ênnan tape 48_frame_76537.jpg]

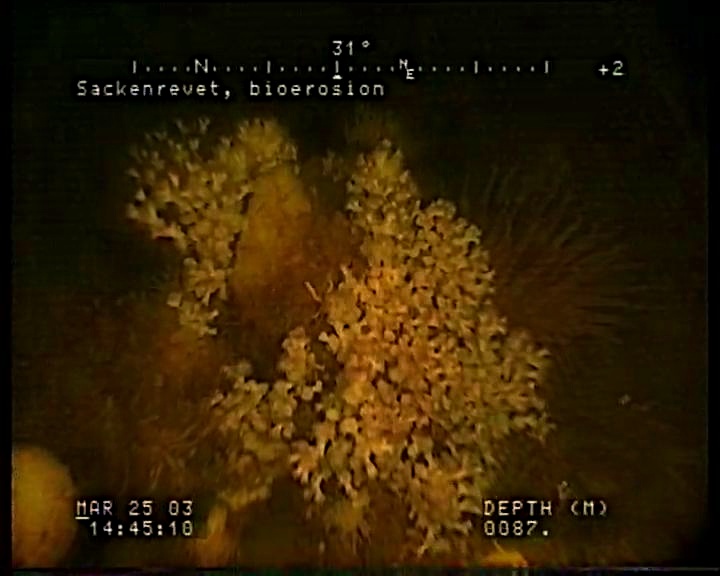

Supplement: Supplementary material 1 — Dataset of underwater images of Desmophyllum pertusum [file bdj-09-e60548-s001.zip › images_new/030325 TMBL-ROV 2003 Sa╠êckenrevet bioerosion_frame_165750.jpg]

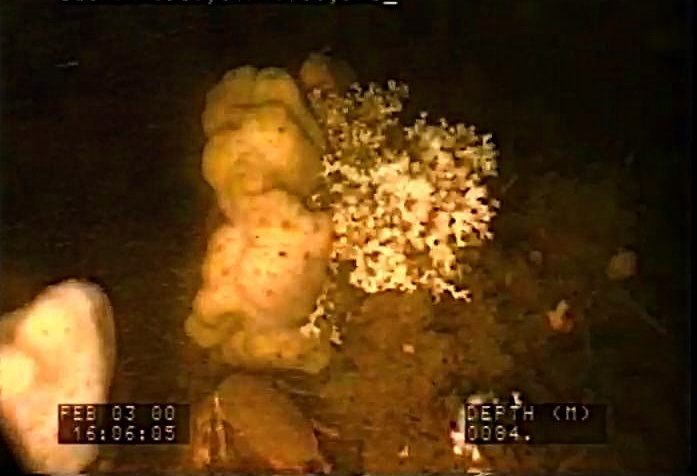

Supplement: Supplementary material 1 — Dataset of underwater images of Desmophyllum pertusum [file bdj-09-e60548-s001.zip › images_new/000203 TMBL-ROV 2000 Sa╠êcken revet EJ numrerade band_frame_48775.jpg]

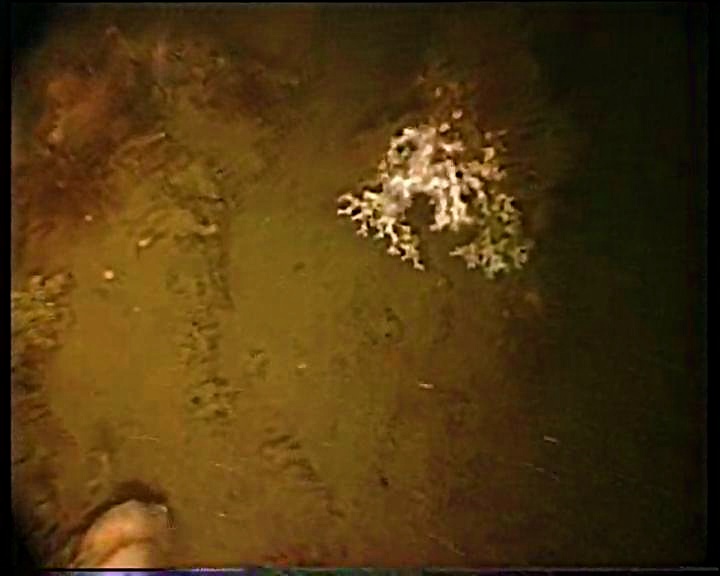

Supplement: Supplementary material 1 — Dataset of underwater images of Desmophyllum pertusum [file bdj-09-e60548-s001.zip › images_new/000203 TMBL-ROV 2000 Sa╠êckenrevet Tape 56_frame_49525.jpg]

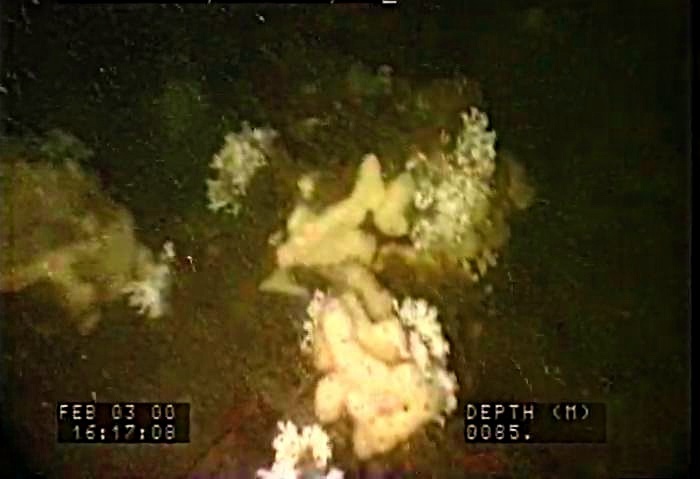

Supplement: Supplementary material 1 — Dataset of underwater images of Desmophyllum pertusum [file bdj-09-e60548-s001.zip › images_new/000203 TMBL-ROV 2000 Sa╠êcken EJ numrerade band_frame_3775.jpg]

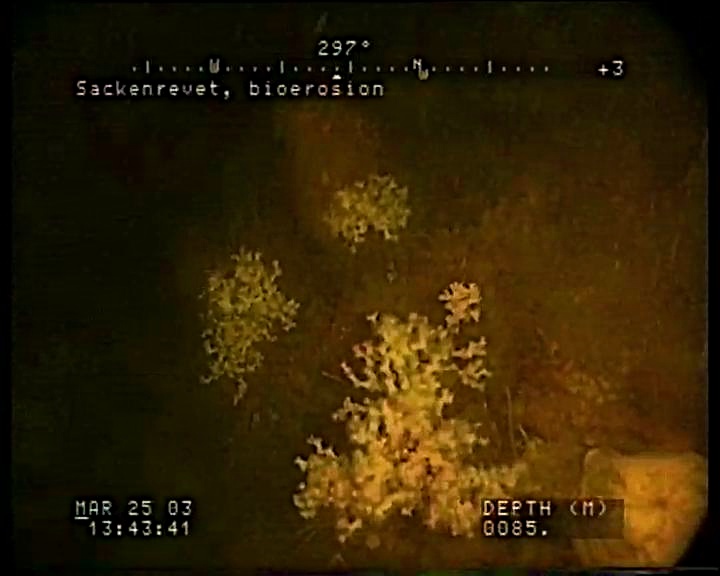

Supplement: Supplementary material 1 — Dataset of underwater images of Desmophyllum pertusum [file bdj-09-e60548-s001.zip › images_new/030325 TMBL-ROV 2003 Sa╠êckenrevet bioerosion_frame_73525.jpg]

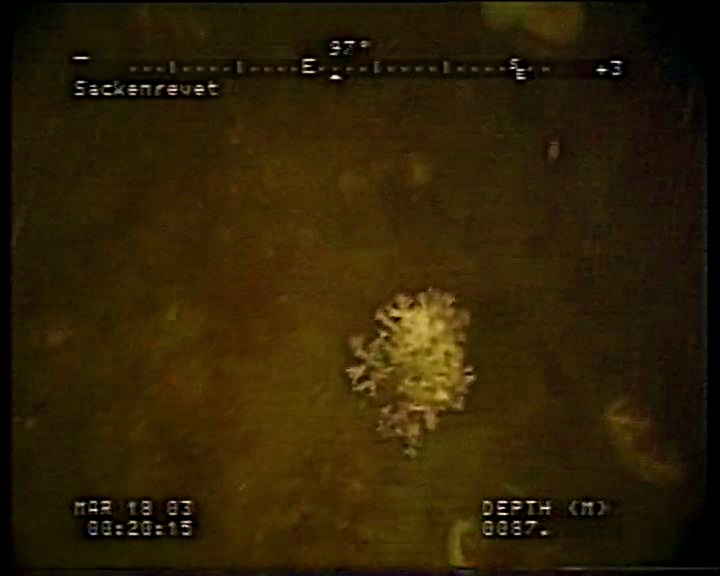

Supplement: Supplementary material 1 — Dataset of underwater images of Desmophyllum pertusum [file bdj-09-e60548-s001.zip › images_new/030317-18 TMBL-ROV 2003 Sa╠êckenrevet_frame_118525.jpg]

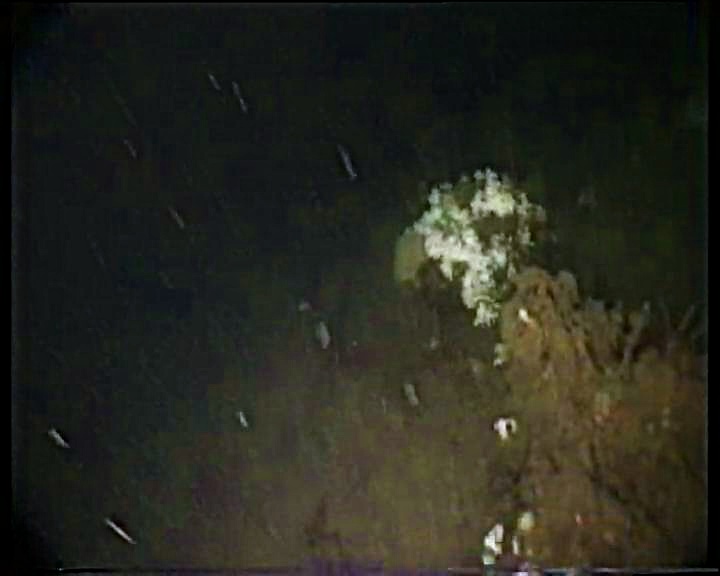

Supplement: Supplementary material 1 — Dataset of underwater images of Desmophyllum pertusum [file bdj-09-e60548-s001.zip › images_new/990506 TMBL-ROV 1999 Revet Sa╠êcken 2 Tape 42_frame_30025.jpg]

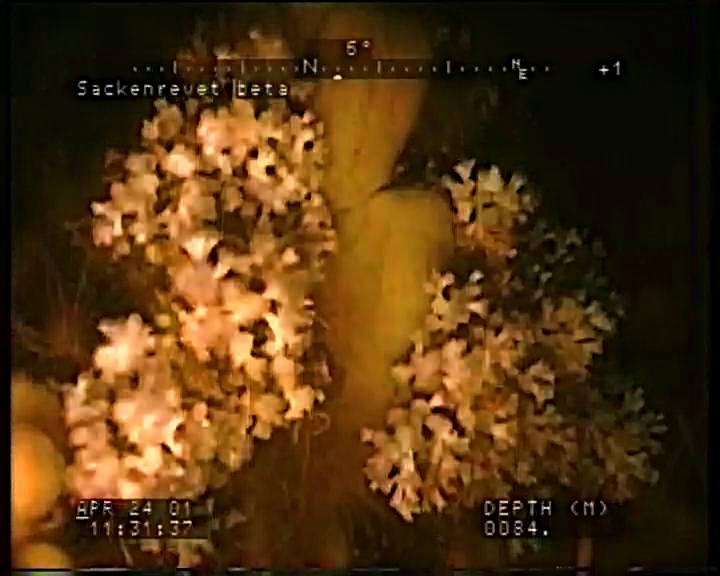

Supplement: Supplementary material 1 — Dataset of underwater images of Desmophyllum pertusum [file bdj-09-e60548-s001.zip › images_new/010424 Sa╠êckenrevet beta Tape 74_frame_45750.jpg]

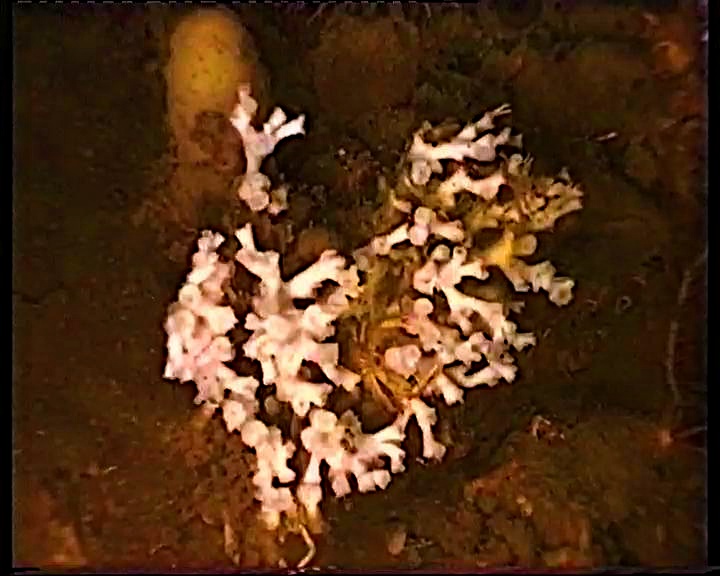

Supplement: Supplementary material 1 — Dataset of underwater images of Desmophyllum pertusum [file bdj-09-e60548-s001.zip › images_new/000114 TMBL-ROV 2000 Sa╠êckenrevet EJ numrerade band_frame_27750.jpg]

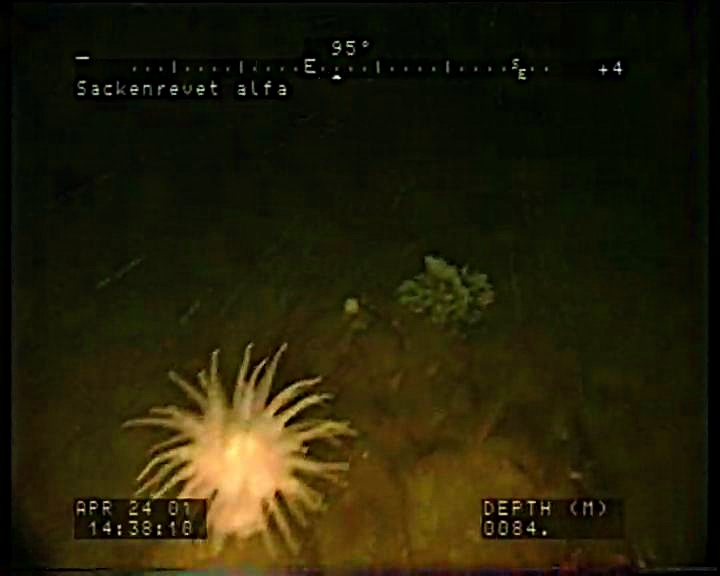

Supplement: Supplementary material 1 — Dataset of underwater images of Desmophyllum pertusum [file bdj-09-e60548-s001.zip › images_new/010424 Sa╠êckenrevet alfa Tape 74_frame_126775.jpg]

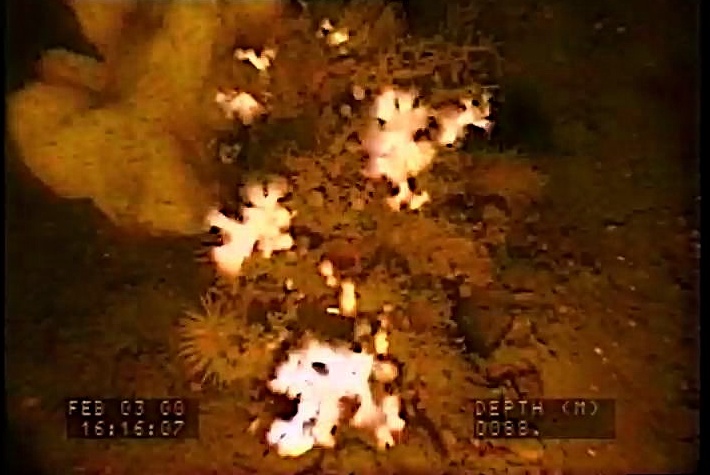

Supplement: Supplementary material 1 — Dataset of underwater images of Desmophyllum pertusum [file bdj-09-e60548-s001.zip › images_new/000203 TMBL-ROV 2000 Sa╠êcken EJ numrerade band_frame_2250.jpg]

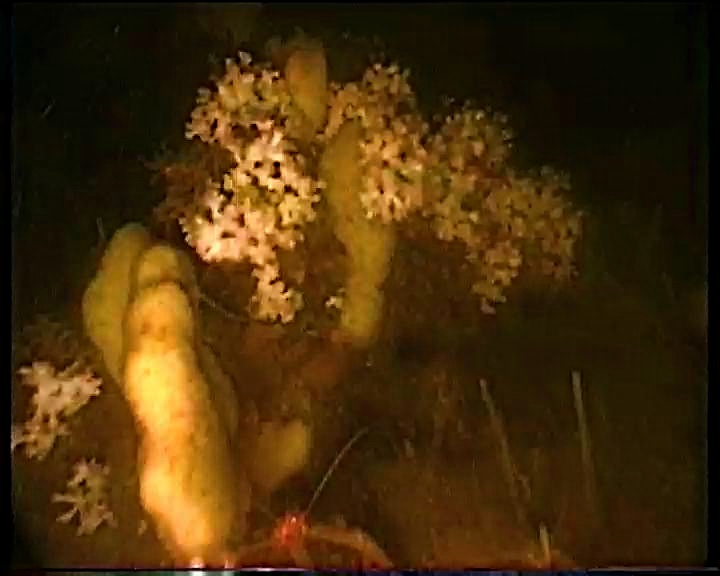

Supplement: Supplementary material 1 — Dataset of underwater images of Desmophyllum pertusum [file bdj-09-e60548-s001.zip › images_new/000114 TMBL-ROV 2000 Sa╠êckenrevet Tape 55_frame_208500.jpg]

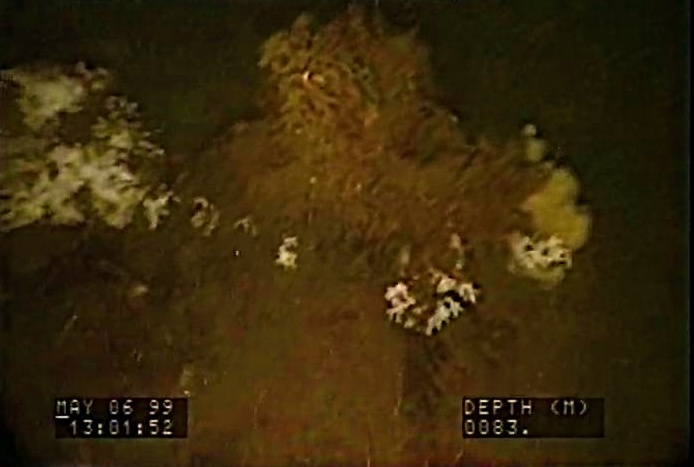

Supplement: Supplementary material 1 — Dataset of underwater images of Desmophyllum pertusum [file bdj-09-e60548-s001.zip › images_new/990506 TMBL-ROV 1999 Revet Sa╠êcken 2 Tape 42_frame_61525.jpg]

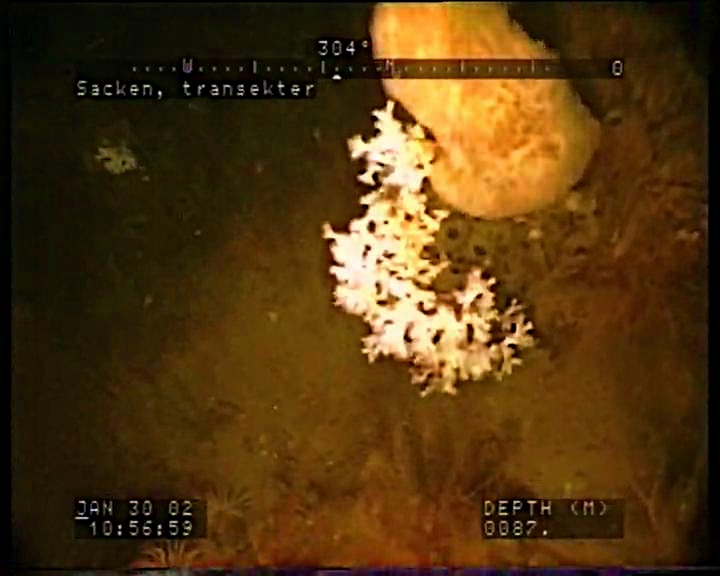

Supplement: Supplementary material 1 — Dataset of underwater images of Desmophyllum pertusum [file bdj-09-e60548-s001.zip › images_new/020130 TMBL-ROV 2002 Sa╠êcken transekt 1_frame_12775.jpg]

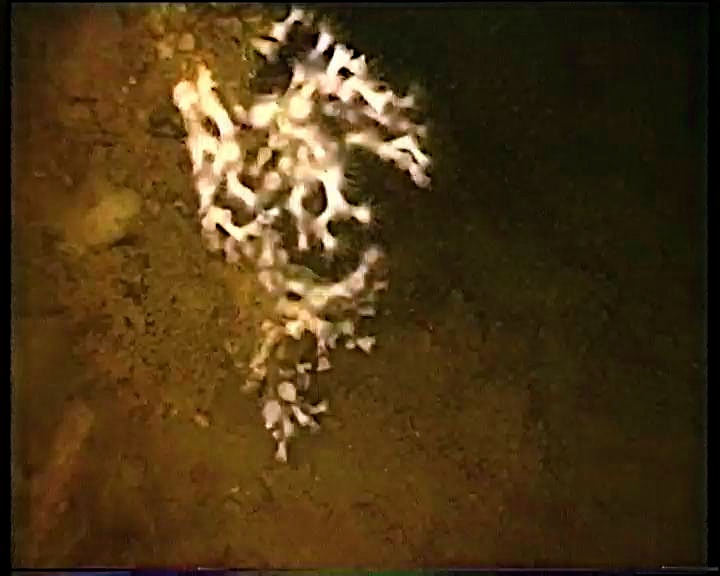

Supplement: Supplementary material 1 — Dataset of underwater images of Desmophyllum pertusum [file bdj-09-e60548-s001.zip › images_new/000203 TMBL-ROV 2000 Sa╠êckenrevet Tape 56_frame_52500.jpg]

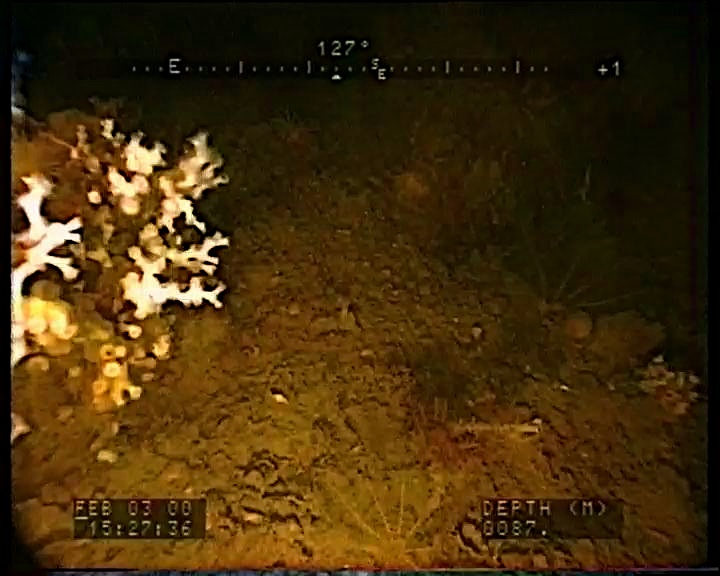

Supplement: Supplementary material 1 — Dataset of underwater images of Desmophyllum pertusum [file bdj-09-e60548-s001.zip › images_new/000203 TMBL-ROV 2000 Sa╠êckenrevet Tape 56_frame_27000.jpg]

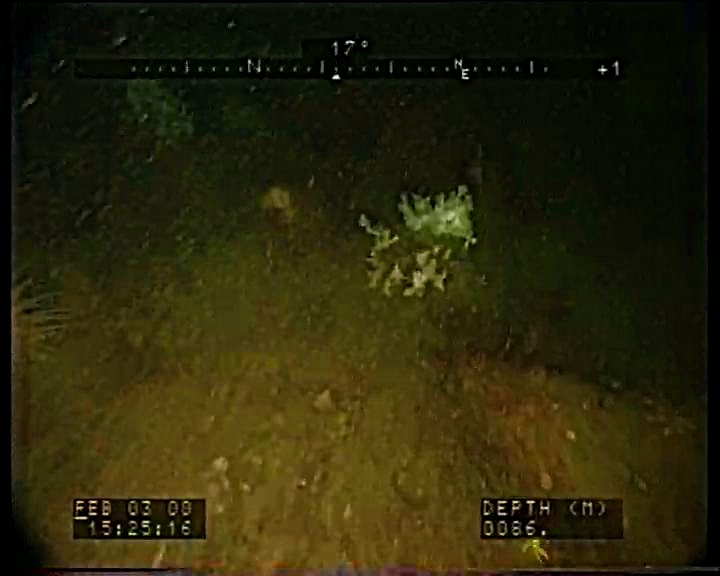

Supplement: Supplementary material 1 — Dataset of underwater images of Desmophyllum pertusum [file bdj-09-e60548-s001.zip › images_new/000203 TMBL-ROV 2000 Sa╠êckenrevet Tape 56_frame_23500.jpg]

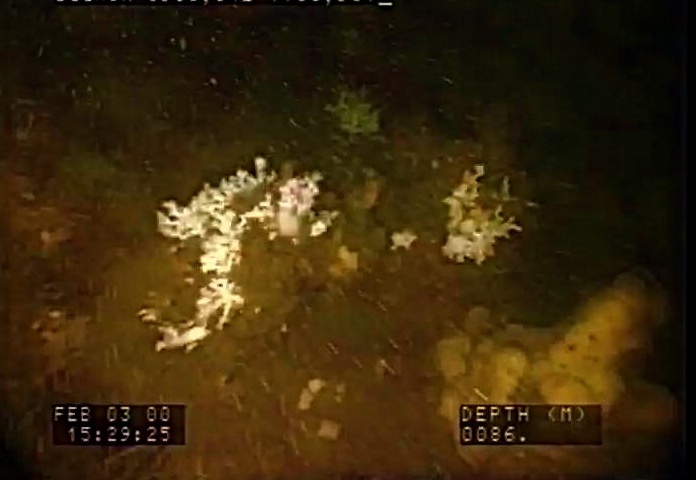

Supplement: Supplementary material 1 — Dataset of underwater images of Desmophyllum pertusum [file bdj-09-e60548-s001.zip › images_new/000203 TMBL-ROV 2000 Sa╠êcken revet EJ numrerade band_frame_3000.jpg]

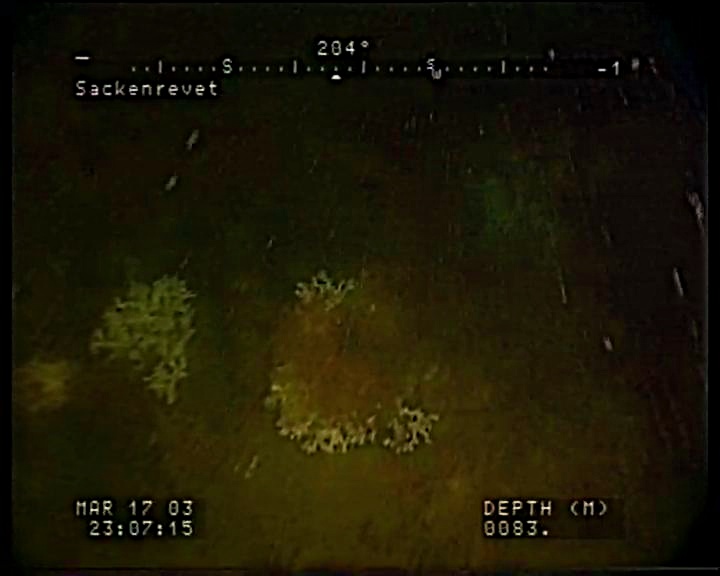

Supplement: Supplementary material 1 — Dataset of underwater images of Desmophyllum pertusum [file bdj-09-e60548-s001.zip › images_new/030317-18 TMBL-ROV 2003 Sa╠êckenrevet_frame_9025.jpg]
