# Supplementary figures and images for: An open-source, citizen science and machine learning approach to analyse subsea movies (part 1 of 2)
Source: Biodivers Data J. 2021 Feb 24;9:e60548. doi: 10.3897/BDJ.9.e60548 (PMC7930014; doi:10.3897/BDJ.9.e60548)

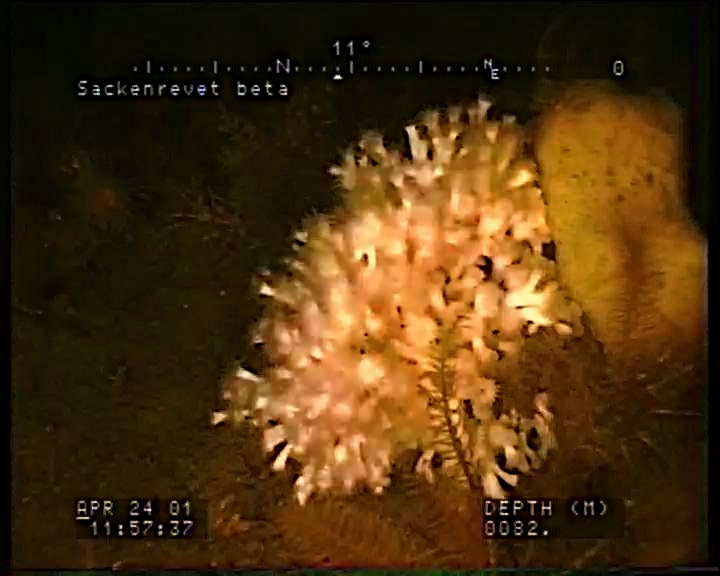

Supplement: Supplementary material 1 — Dataset of underwater images of Desmophyllum pertusum [file bdj-09-e60548-s001.zip › images_new/010424 Sa╠êckenrevet beta Tape 74_frame_84750.jpg]

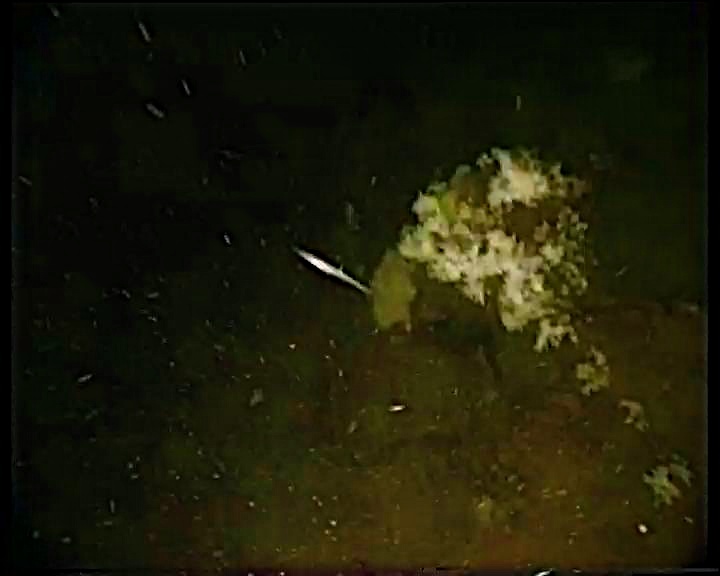

Supplement: Supplementary material 1 — Dataset of underwater images of Desmophyllum pertusum [file bdj-09-e60548-s001.zip › images_new/990506 TMBL-ROV 1999 Revet Sa╠êcken 2 Tape 42_frame_32250.jpg]

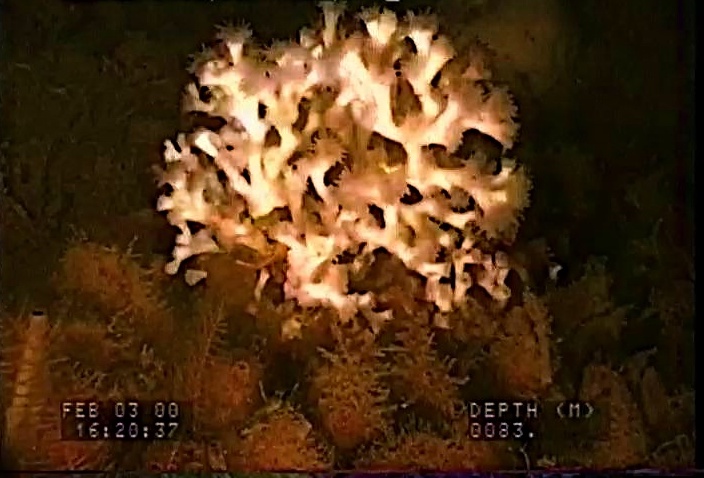

Supplement: Supplementary material 1 — Dataset of underwater images of Desmophyllum pertusum [file bdj-09-e60548-s001.zip › images_new/000203 TMBL-ROV 2000 Sa╠êckenrevet Tape 56_frame_106500.jpg]

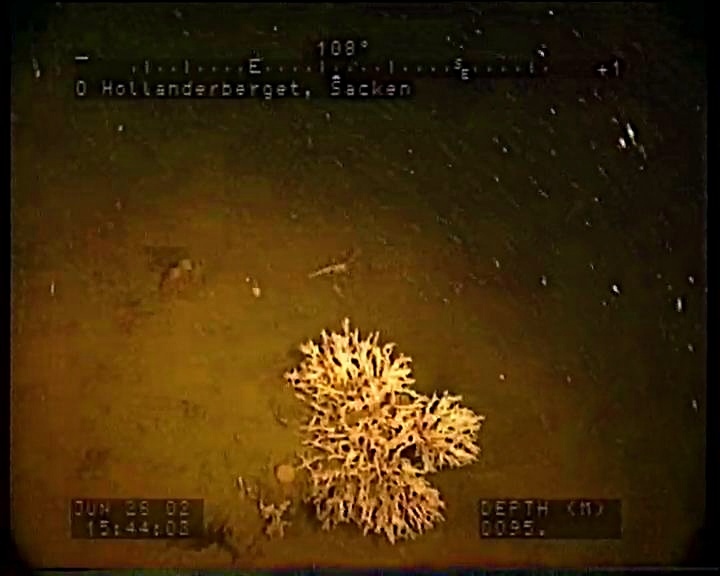

Supplement: Supplementary material 1 — Dataset of underwater images of Desmophyllum pertusum [file bdj-09-e60548-s001.zip › images_new/020628 TMBL-ROV 2002 O Hollanderberget Sa╠êcken_frame_112525.jpg]

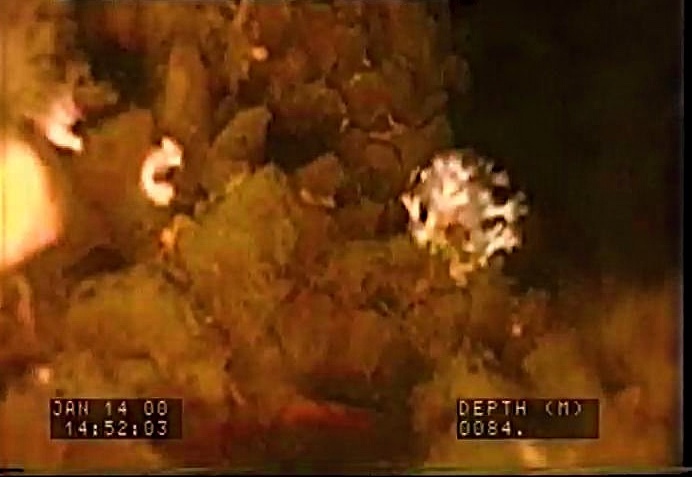

Supplement: Supplementary material 1 — Dataset of underwater images of Desmophyllum pertusum [file bdj-09-e60548-s001.zip › images_new/000114 TMBL-ROV 2000 Sa╠êckenrevet EJ numrerade band_frame_42025.jpg]

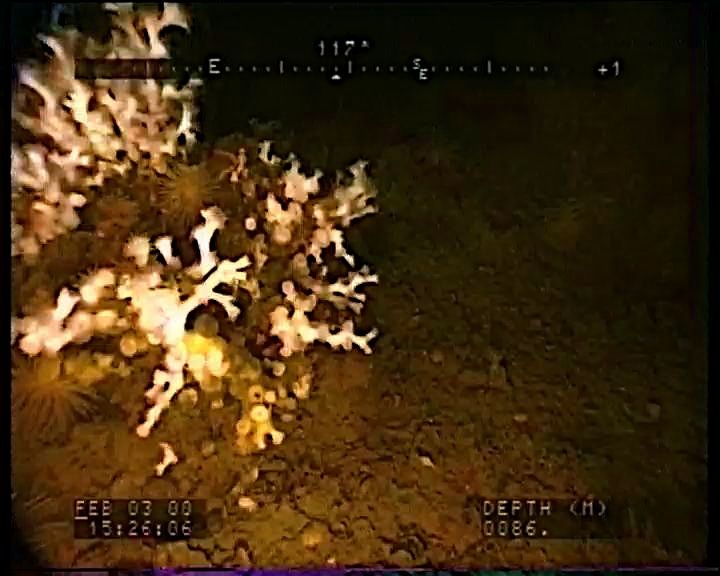

Supplement: Supplementary material 1 — Dataset of underwater images of Desmophyllum pertusum [file bdj-09-e60548-s001.zip › images_new/000203 TMBL-ROV 2000 Sa╠êckenrevet Tape 56_frame_24750.jpg]

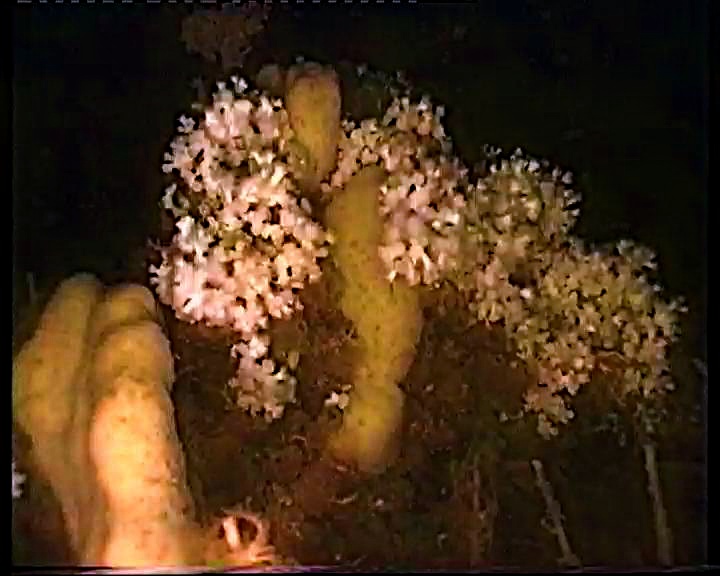

Supplement: Supplementary material 1 — Dataset of underwater images of Desmophyllum pertusum [file bdj-09-e60548-s001.zip › images_new/000114 TMBL-ROV 2000 Sa╠êckenrevet EJ numrerade band_frame_46525.jpg]

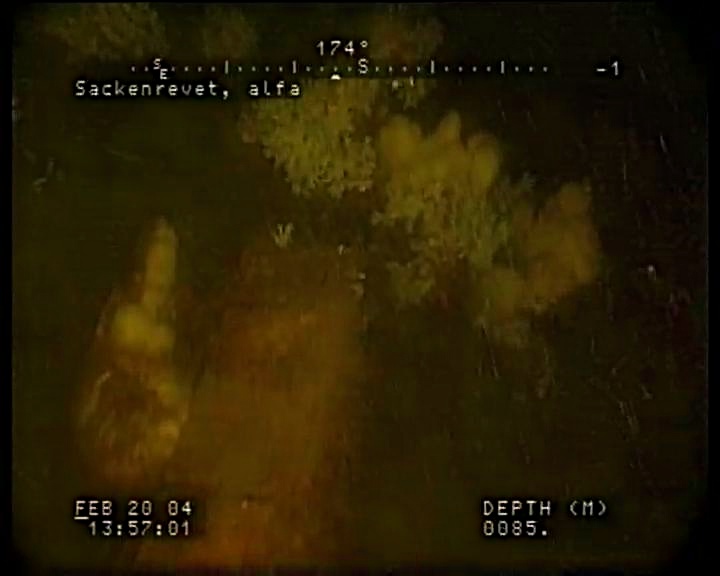

Supplement: Supplementary material 1 — Dataset of underwater images of Desmophyllum pertusum [file bdj-09-e60548-s001.zip › images_new/040220 TMBL-ROV 2004 Sa╠êckenrevet alfa_frame_30875.jpg]

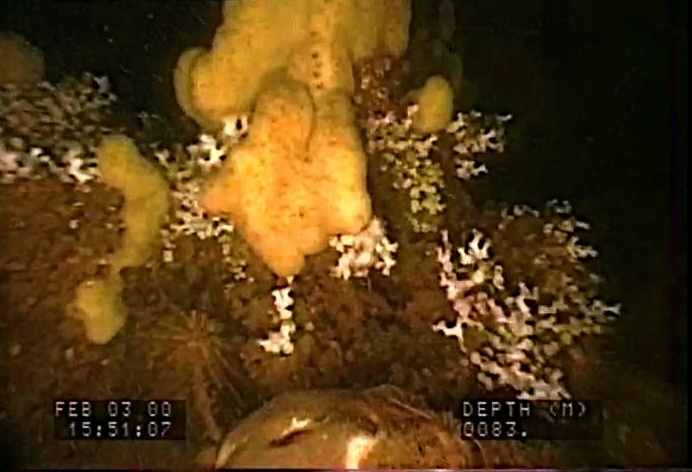

Supplement: Supplementary material 1 — Dataset of underwater images of Desmophyllum pertusum [file bdj-09-e60548-s001.zip › images_new/000203 TMBL-ROV 2000 Sa╠êckenrevet Tape 56_frame_62275.jpg]

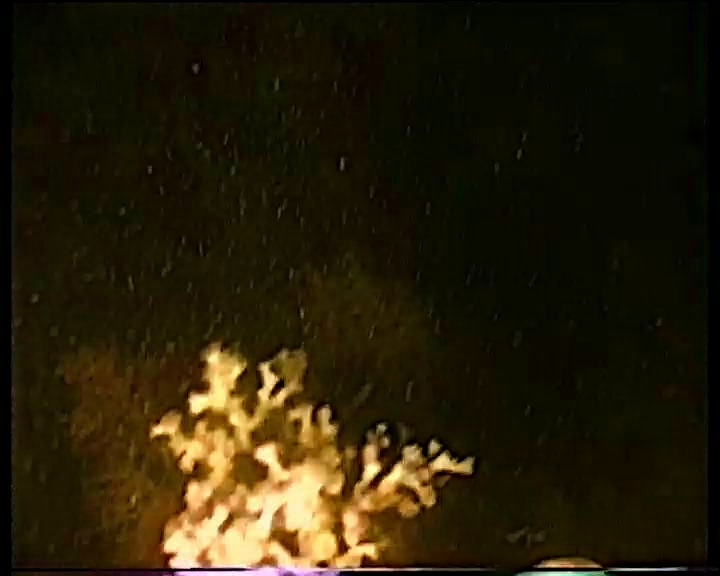

Supplement: Supplementary material 1 — Dataset of underwater images of Desmophyllum pertusum [file bdj-09-e60548-s001.zip › images_new/000203 TMBL-ROV 2000 Sa╠êckenrevet Tape 56_frame_36750.jpg]

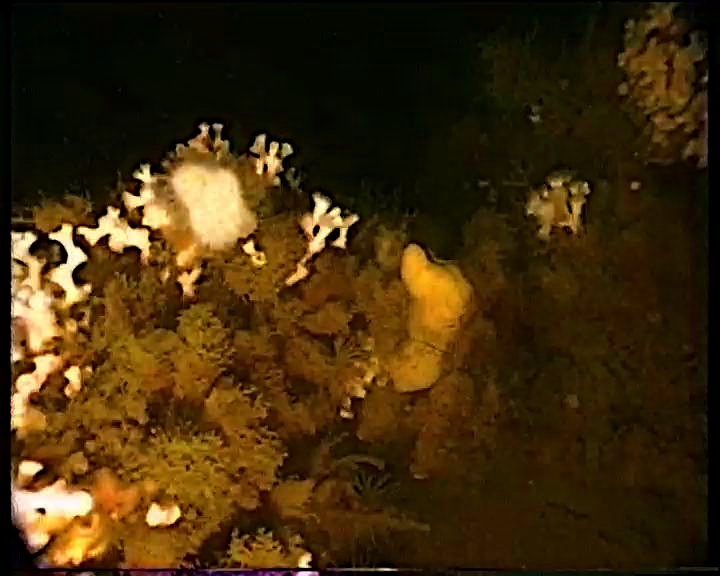

Supplement: Supplementary material 1 — Dataset of underwater images of Desmophyllum pertusum [file bdj-09-e60548-s001.zip › images_new/000203 TMBL-ROV 2000 Sa╠êckenrevet Tape 56_frame_32250.jpg]

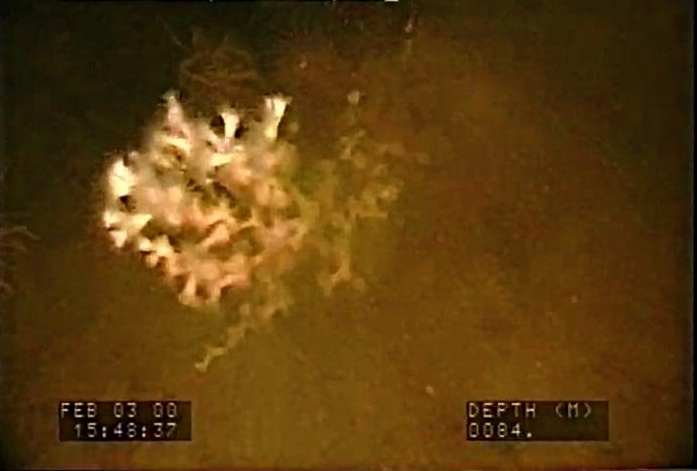

Supplement: Supplementary material 1 — Dataset of underwater images of Desmophyllum pertusum [file bdj-09-e60548-s001.zip › images_new/000203 TMBL-ROV 2000 Sa╠êckenrevet Tape 56_frame_58512.jpg]

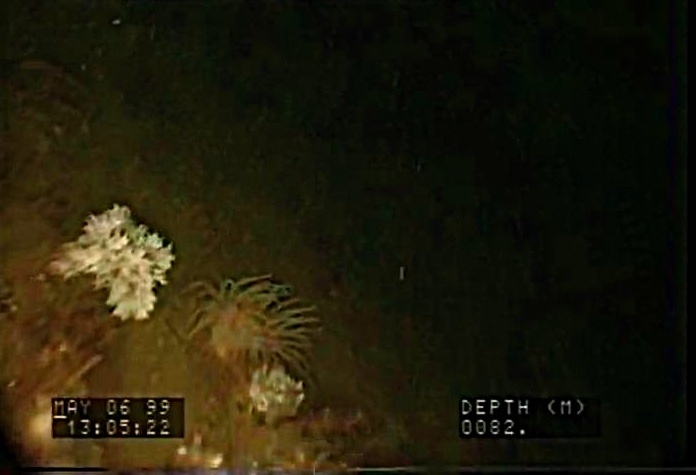

Supplement: Supplementary material 1 — Dataset of underwater images of Desmophyllum pertusum [file bdj-09-e60548-s001.zip › images_new/990506 TMBL-ROV 1999 Revet Sa╠êcken 2 Tape 42_frame_66775.jpg]

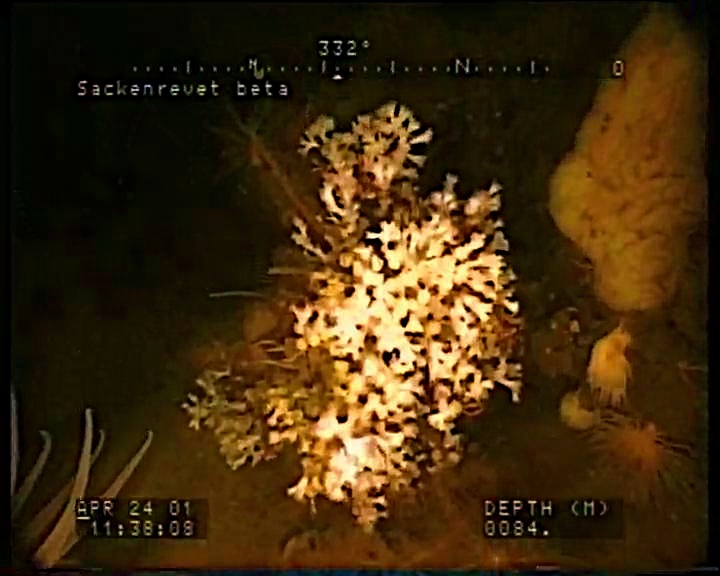

Supplement: Supplementary material 1 — Dataset of underwater images of Desmophyllum pertusum [file bdj-09-e60548-s001.zip › images_new/010424 Sa╠êckenrevet beta Tape 74_frame_55525.jpg]

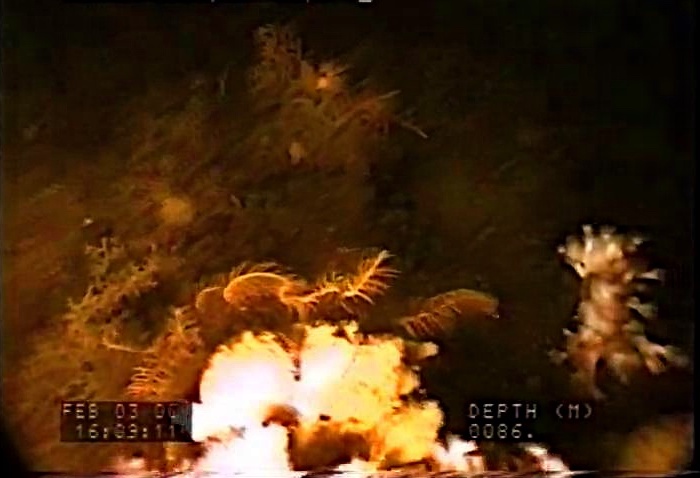

Supplement: Supplementary material 1 — Dataset of underwater images of Desmophyllum pertusum [file bdj-09-e60548-s001.zip › images_new/000203 TMBL-ROV 2000 Sa╠êcken revet EJ numrerade band_frame_53287.jpg]

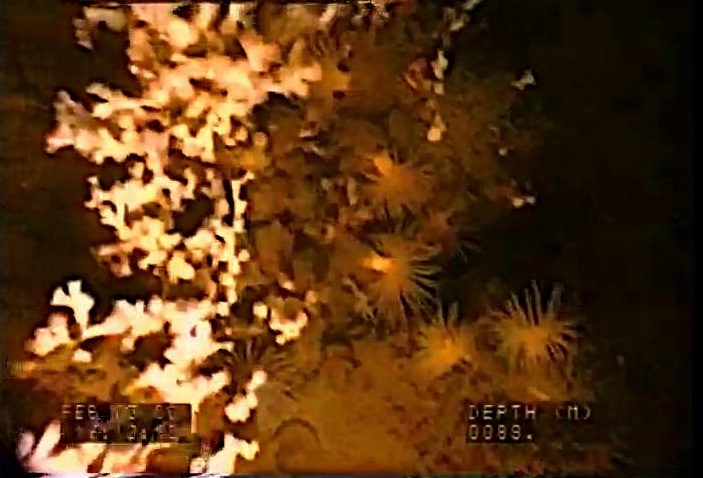

Supplement: Supplementary material 1 — Dataset of underwater images of Desmophyllum pertusum [file bdj-09-e60548-s001.zip › images_new/000203 TMBL-ROV 2000 Sa╠êcken revet EJ numrerade band_frame_57750.jpg]

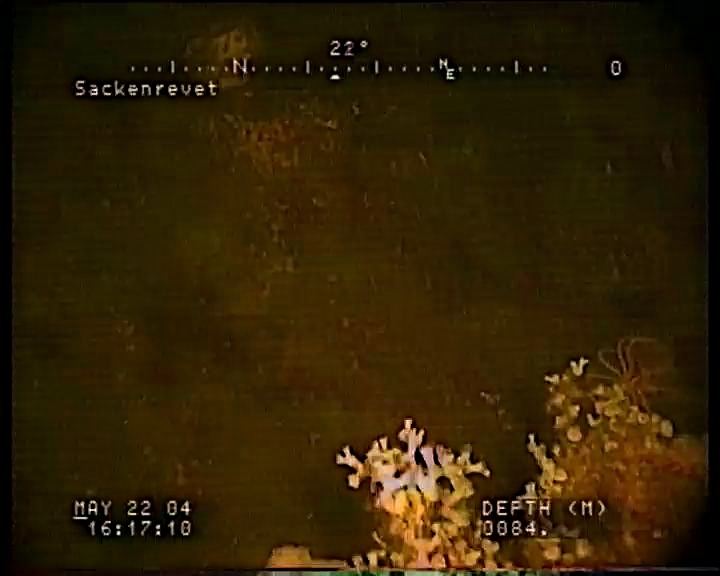

Supplement: Supplementary material 1 — Dataset of underwater images of Desmophyllum pertusum [file bdj-09-e60548-s001.zip › images_new/040522 TMBL-ROV 2004 Sa╠êckenrevet_frame_4500.jpg]

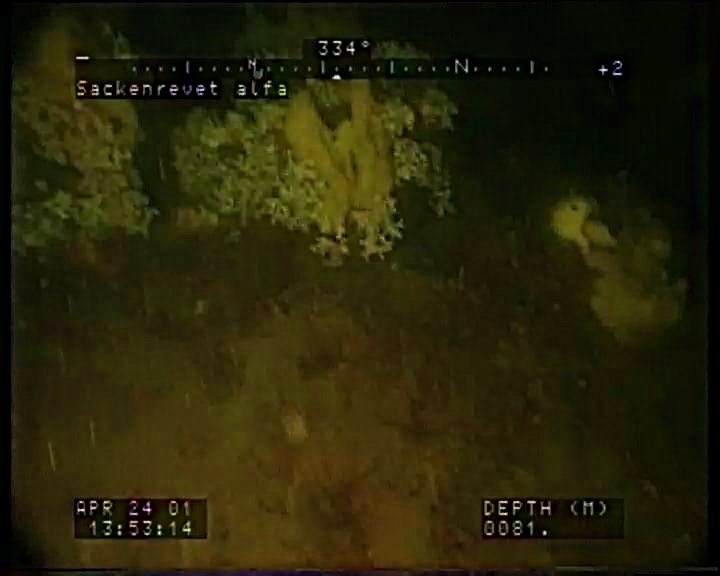

Supplement: Supplementary material 1 — Dataset of underwater images of Desmophyllum pertusum [file bdj-09-e60548-s001.zip › images_new/010424 Sa╠êckenrevet alfa Tape 74_frame_59400.jpg]

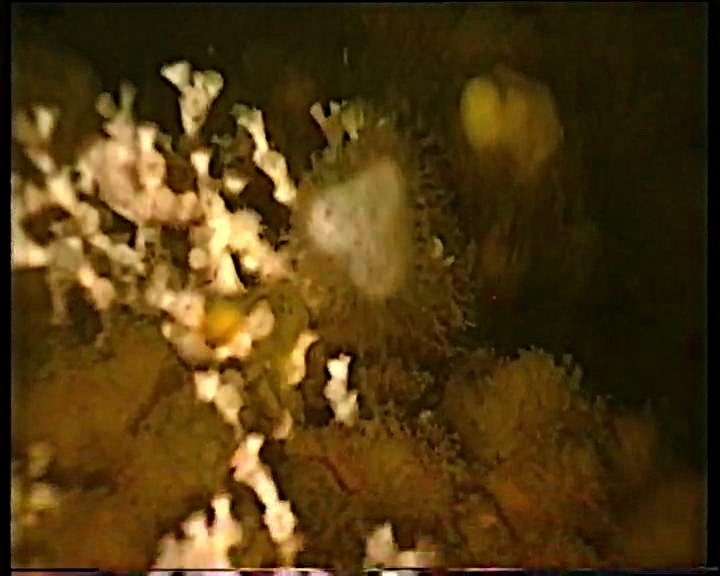

Supplement: Supplementary material 1 — Dataset of underwater images of Desmophyllum pertusum [file bdj-09-e60548-s001.zip › images_new/000203 TMBL-ROV 2000 Sa╠êckenrevet Tape 56_frame_33775.jpg]

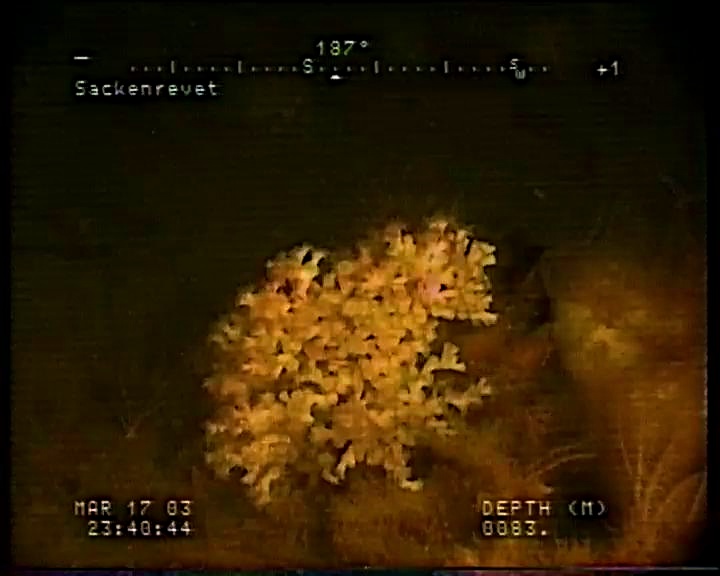

Supplement: Supplementary material 1 — Dataset of underwater images of Desmophyllum pertusum [file bdj-09-e60548-s001.zip › images_new/030317-18 TMBL-ROV 2003 Sa╠êckenrevet_frame_59250.jpg]

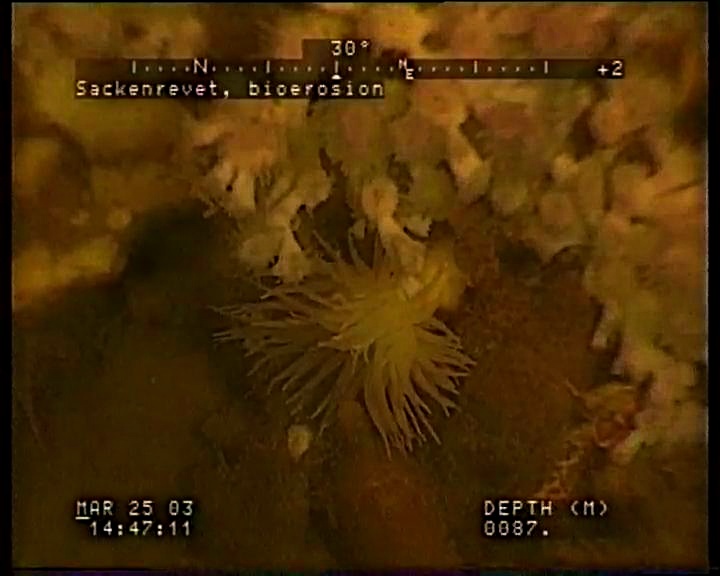

Supplement: Supplementary material 1 — Dataset of underwater images of Desmophyllum pertusum [file bdj-09-e60548-s001.zip › images_new/030325 TMBL-ROV 2003 Sa╠êckenrevet bioerosion_frame_168775.jpg]

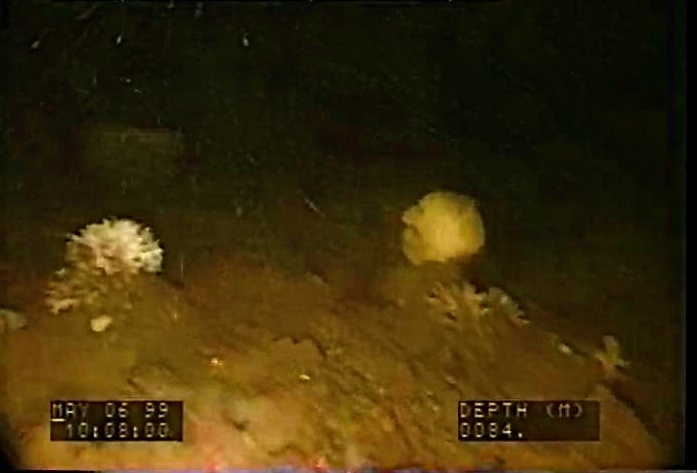

Supplement: Supplementary material 1 — Dataset of underwater images of Desmophyllum pertusum [file bdj-09-e60548-s001.zip › images_new/990506 TMBL-ROV 1999 Revet Sa╠êcken Tape 42_SELECTWS_frame_825.jpg]

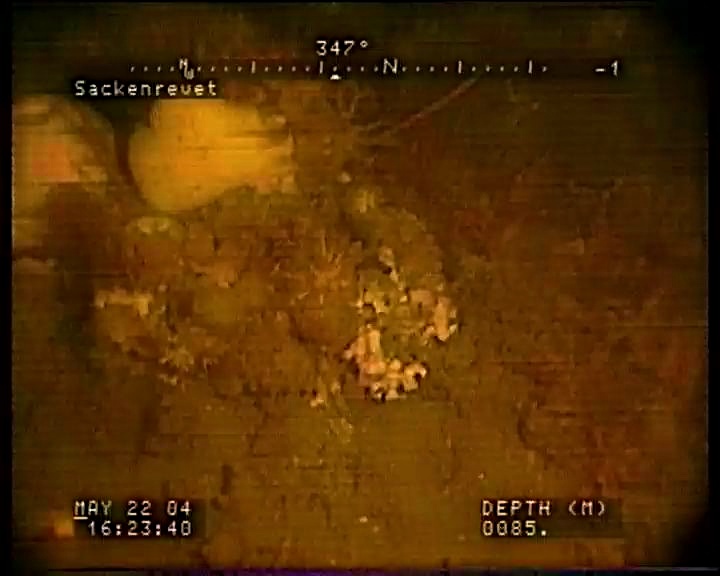

Supplement: Supplementary material 1 — Dataset of underwater images of Desmophyllum pertusum [file bdj-09-e60548-s001.zip › images_new/040522 TMBL-ROV 2004 Sa╠êckenrevet_frame_14250.jpg]

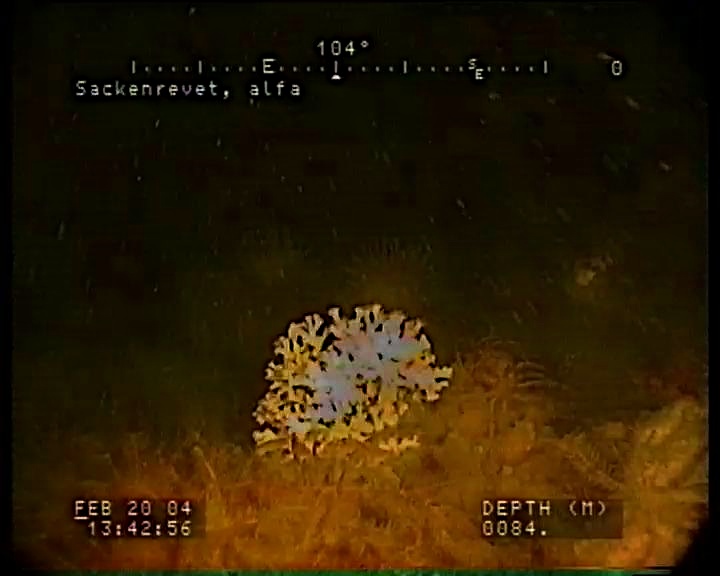

Supplement: Supplementary material 1 — Dataset of underwater images of Desmophyllum pertusum [file bdj-09-e60548-s001.zip › images_new/040220 TMBL-ROV 2004 Sa╠êckenrevet alfa_frame_9750.jpg]

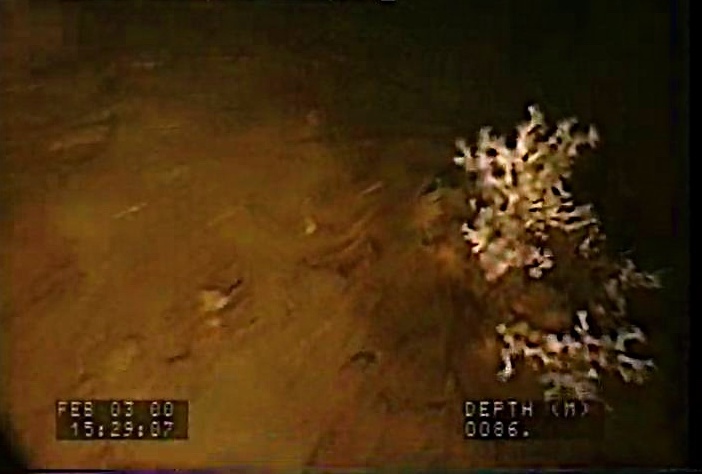

Supplement: Supplementary material 1 — Dataset of underwater images of Desmophyllum pertusum [file bdj-09-e60548-s001.zip › images_new/000203 TMBL-ROV 2000 Sa╠êckenrevet Tape 56_frame_29275.jpg]

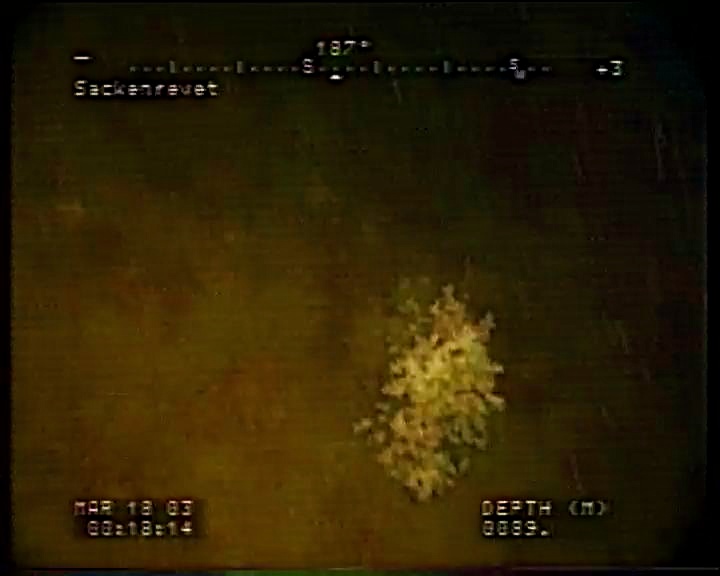

Supplement: Supplementary material 1 — Dataset of underwater images of Desmophyllum pertusum [file bdj-09-e60548-s001.zip › images_new/030317-18 TMBL-ROV 2003 Sa╠êckenrevet_frame_115500.jpg]

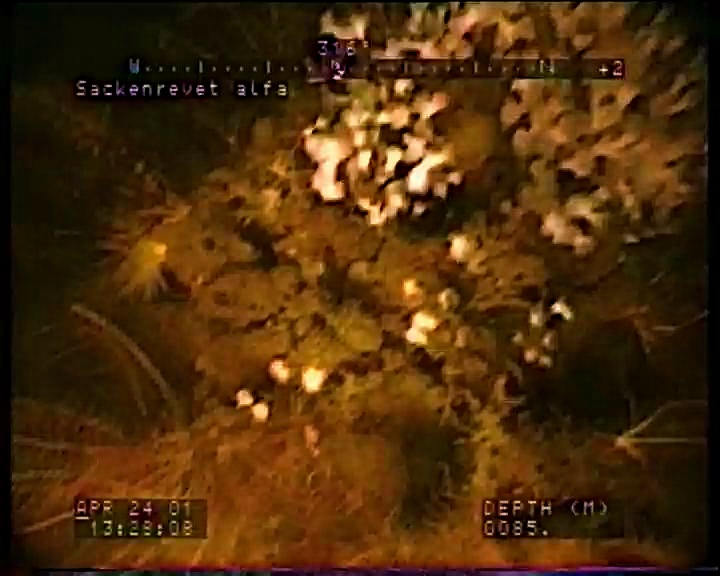

Supplement: Supplementary material 1 — Dataset of underwater images of Desmophyllum pertusum [file bdj-09-e60548-s001.zip › images_new/010424 Sa╠êckenrevet alfa Tape 74_frame_21750.jpg]

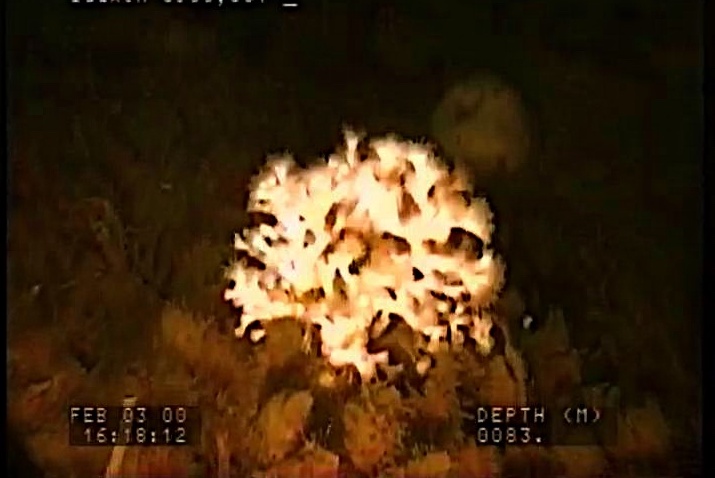

Supplement: Supplementary material 1 — Dataset of underwater images of Desmophyllum pertusum [file bdj-09-e60548-s001.zip › images_new/000203 TMBL-ROV 2000 Sa╠êcken EJ numrerade band_frame_5375.jpg]

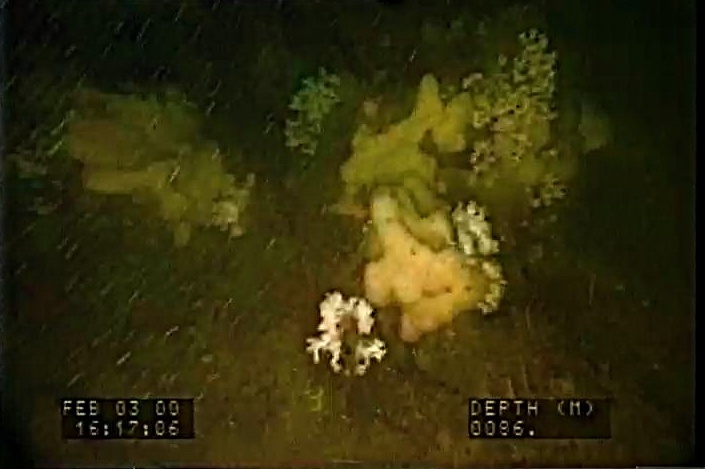

Supplement: Supplementary material 1 — Dataset of underwater images of Desmophyllum pertusum [file bdj-09-e60548-s001.zip › images_new/000203 TMBL-ROV 2000 Sa╠êckenrevet Tape 56_frame_101250.jpg]

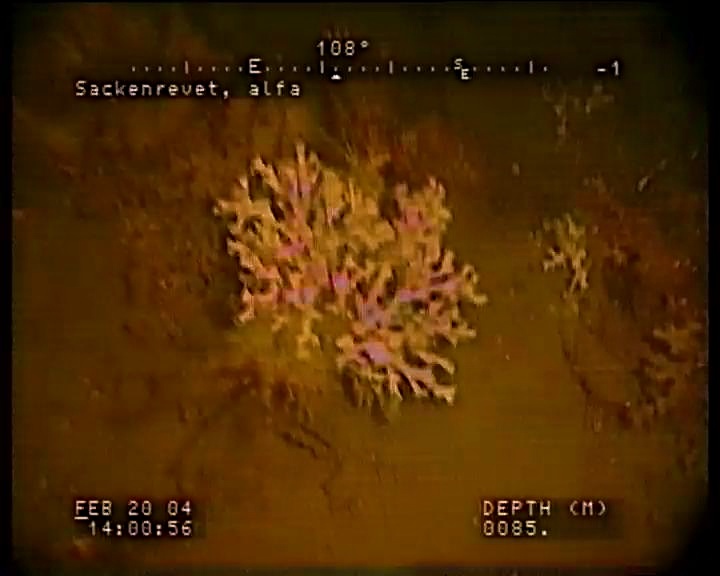

Supplement: Supplementary material 1 — Dataset of underwater images of Desmophyllum pertusum [file bdj-09-e60548-s001.zip › images_new/040220 TMBL-ROV 2004 Sa╠êckenrevet alfa_frame_36750.jpg]

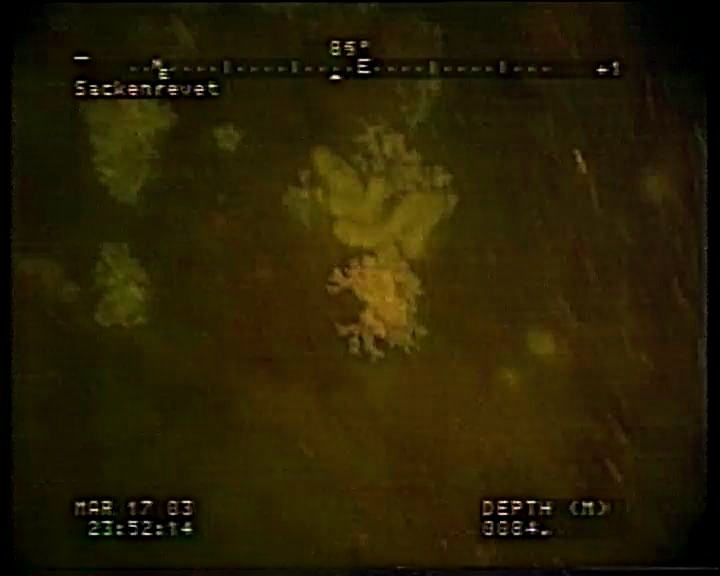

Supplement: Supplementary material 1 — Dataset of underwater images of Desmophyllum pertusum [file bdj-09-e60548-s001.zip › images_new/030317-18 TMBL-ROV 2003 Sa╠êckenrevet_frame_76500.jpg]

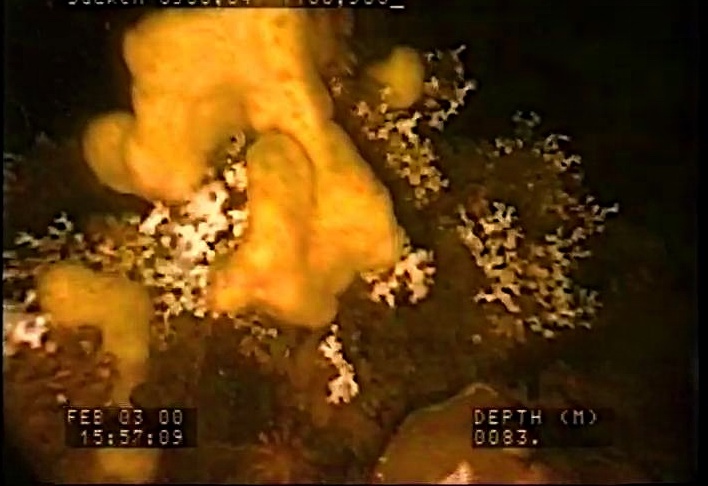

Supplement: Supplementary material 1 — Dataset of underwater images of Desmophyllum pertusum [file bdj-09-e60548-s001.zip › images_new/000203 TMBL-ROV 2000 Sa╠êcken revet EJ numrerade band_frame_36025.jpg]

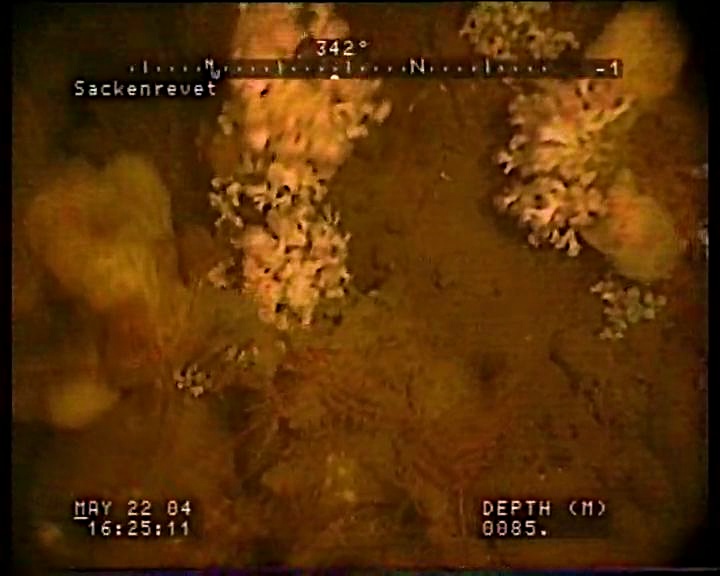

Supplement: Supplementary material 1 — Dataset of underwater images of Desmophyllum pertusum [file bdj-09-e60548-s001.zip › images_new/040522 TMBL-ROV 2004 Sa╠êckenrevet_frame_16525.jpg]

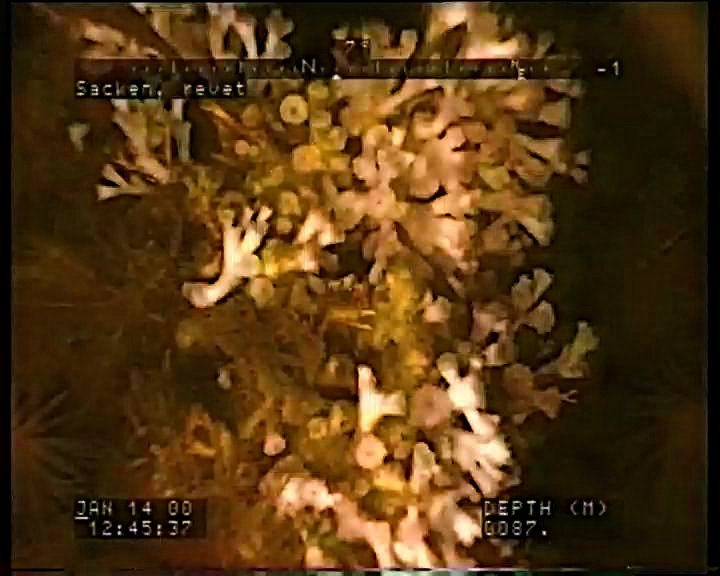

Supplement: Supplementary material 1 — Dataset of underwater images of Desmophyllum pertusum [file bdj-09-e60548-s001.zip › images_new/000114 TMBL-ROV 2000 Sa╠êckenrevet Tape 55_frame_12750.jpg]

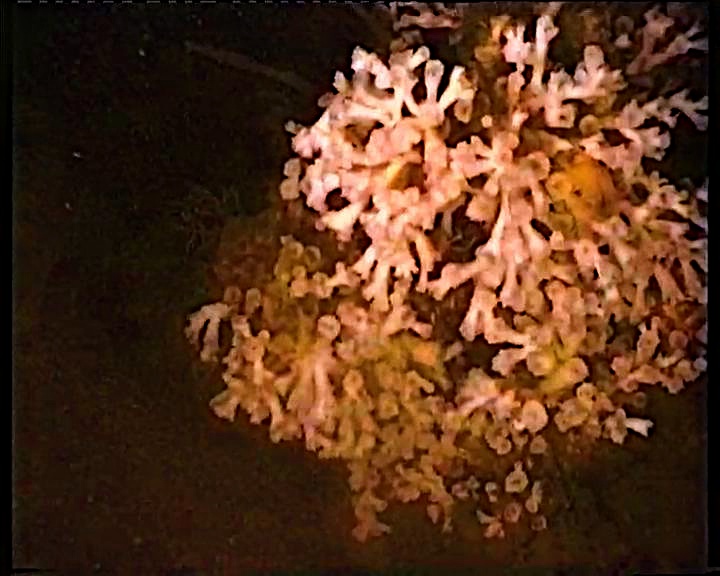

Supplement: Supplementary material 1 — Dataset of underwater images of Desmophyllum pertusum [file bdj-09-e60548-s001.zip › images_new/000114 TMBL-ROV 2000 Sa╠êckenrevet EJ numrerade band_frame_57775.jpg]

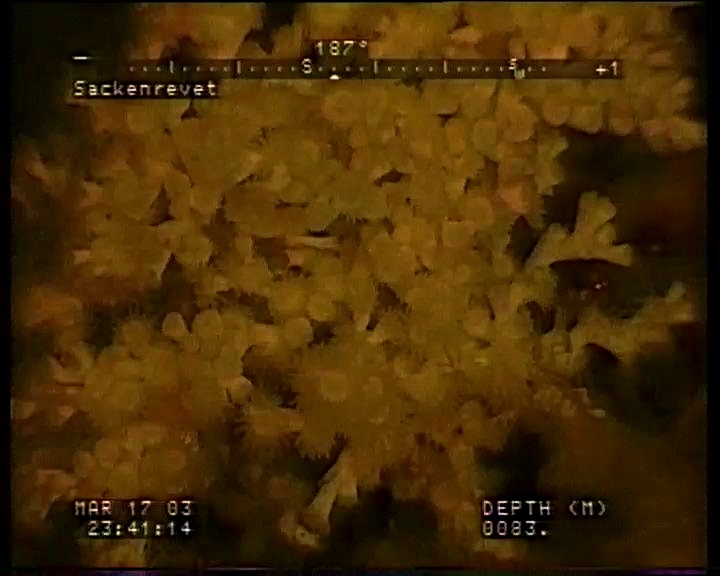

Supplement: Supplementary material 1 — Dataset of underwater images of Desmophyllum pertusum [file bdj-09-e60548-s001.zip › images_new/030317-18 TMBL-ROV 2003 Sa╠êckenrevet_frame_60000.jpg]

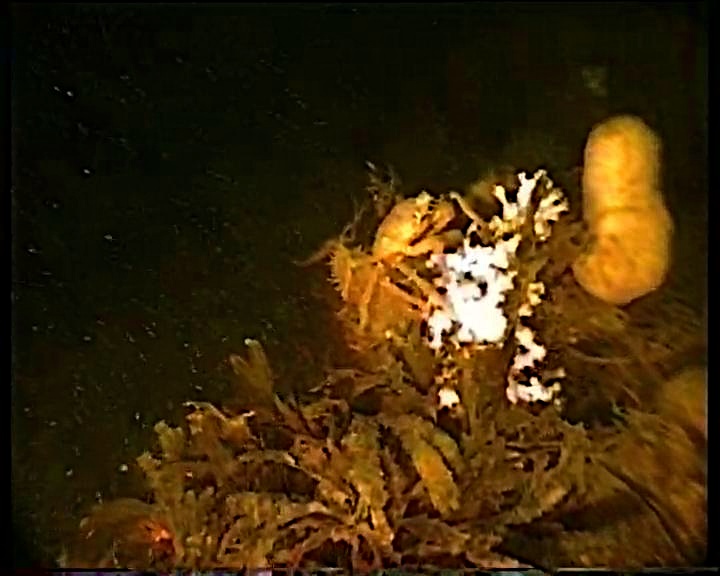

Supplement: Supplementary material 1 — Dataset of underwater images of Desmophyllum pertusum [file bdj-09-e60548-s001.zip › images_new/000114 TMBL-ROV 2000 Sa╠êckenrevet Tape 55_frame_180025.jpg]

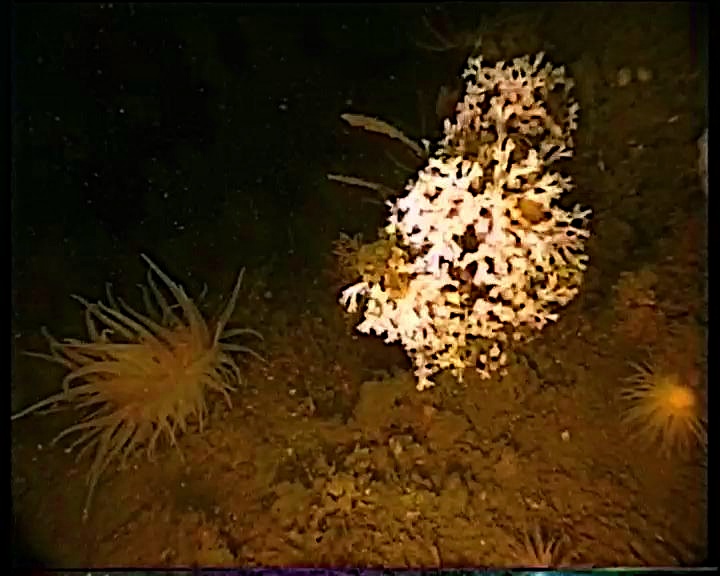

Supplement: Supplementary material 1 — Dataset of underwater images of Desmophyllum pertusum [file bdj-09-e60548-s001.zip › images_new/000114 TMBL-ROV 2000 Sa╠êckenrevet Tape 55_frame_189000.jpg]

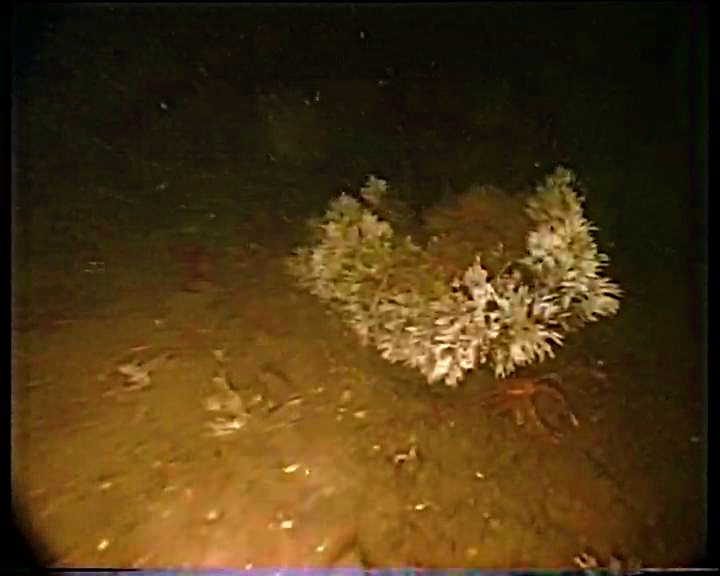

Supplement: Supplementary material 1 — Dataset of underwater images of Desmophyllum pertusum [file bdj-09-e60548-s001.zip › images_new/990506 TMBL-ROV 1999 Revet Sa╠êcken Tape 42_SELECTWS_frame_33187.jpg]

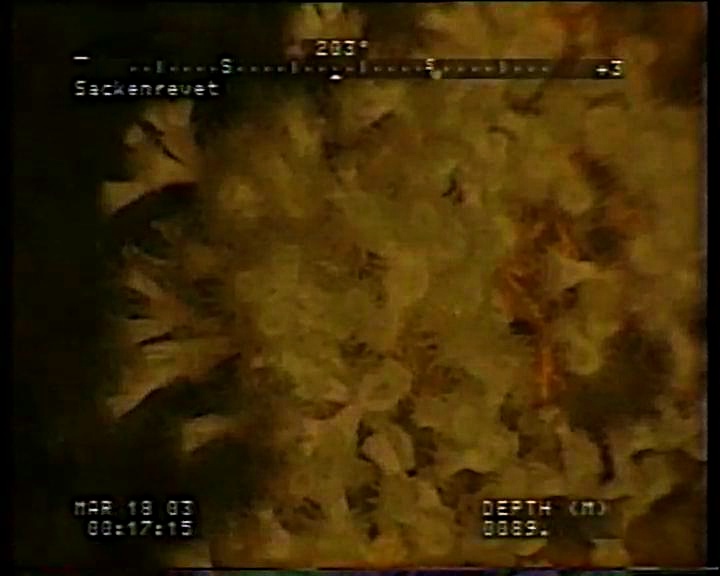

Supplement: Supplementary material 1 — Dataset of underwater images of Desmophyllum pertusum [file bdj-09-e60548-s001.zip › images_new/030317-18 TMBL-ROV 2003 Sa╠êckenrevet_frame_114025.jpg]

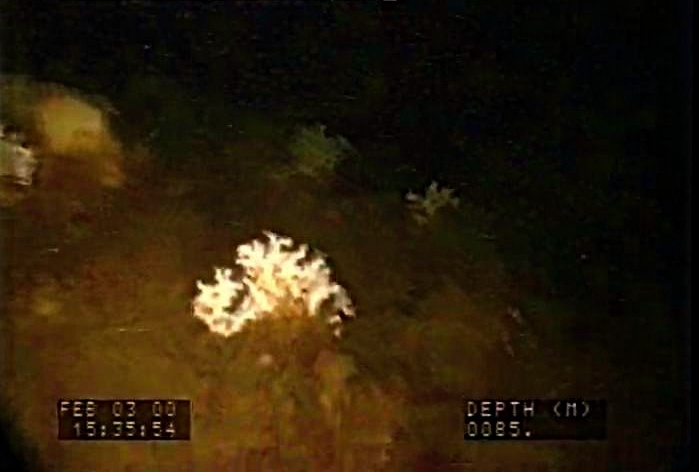

Supplement: Supplementary material 1 — Dataset of underwater images of Desmophyllum pertusum [file bdj-09-e60548-s001.zip › images_new/000203 TMBL-ROV 2000 Sa╠êcken revet EJ numrerade band_frame_12025.jpg]

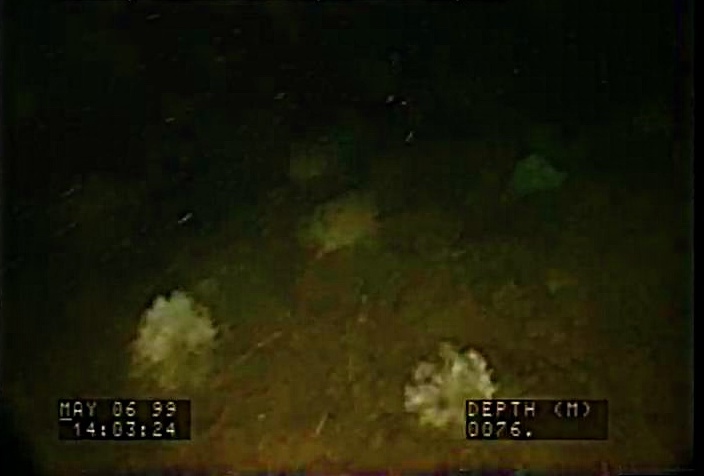

Supplement: Supplementary material 1 — Dataset of underwater images of Desmophyllum pertusum [file bdj-09-e60548-s001.zip › images_new/990506 TMBL-ROV 1999 Revet Sa╠êcken 2 Tape 42_frame_150000.jpg]

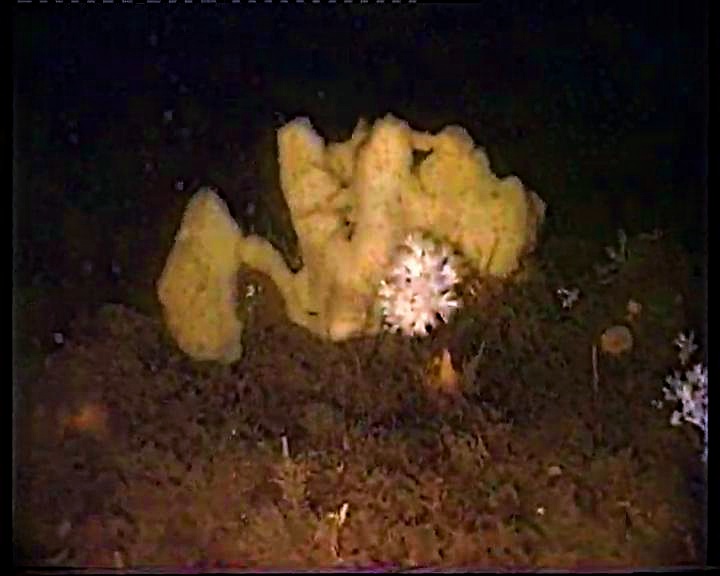

Supplement: Supplementary material 1 — Dataset of underwater images of Desmophyllum pertusum [file bdj-09-e60548-s001.zip › images_new/000114 TMBL-ROV 2000 Sa╠êckenrevet EJ numrerade band_frame_14275.jpg]

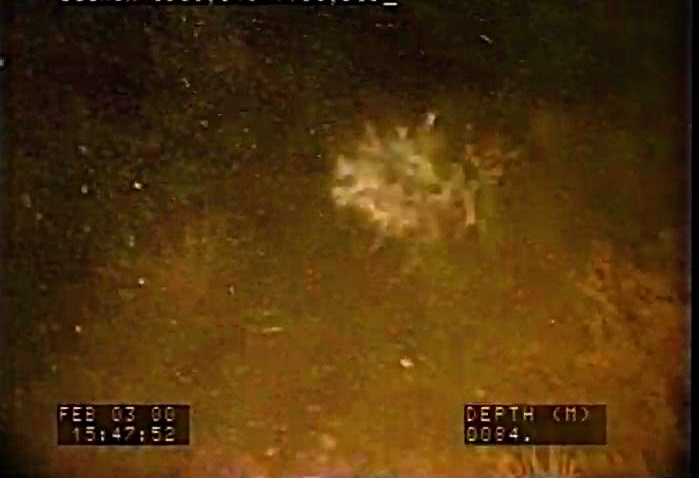

Supplement: Supplementary material 1 — Dataset of underwater images of Desmophyllum pertusum [file bdj-09-e60548-s001.zip › images_new/000203 TMBL-ROV 2000 Sa╠êcken revet EJ numrerade band_frame_25500.jpg]

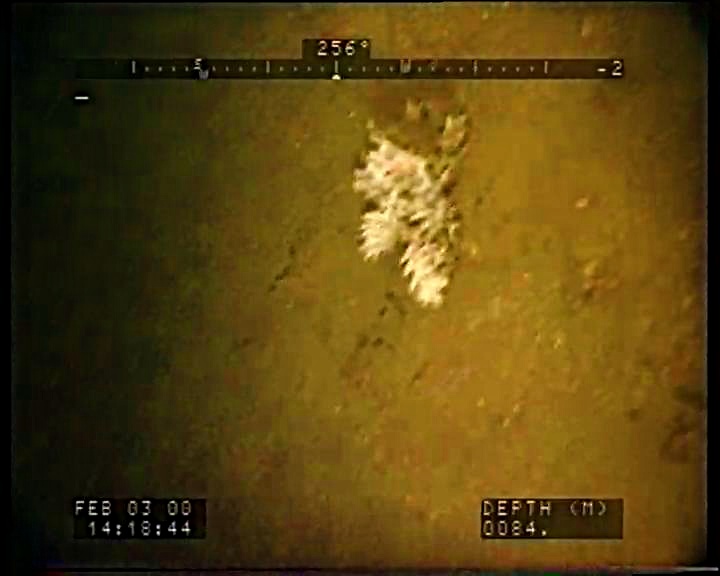

Supplement: Supplementary material 1 — Dataset of underwater images of Desmophyllum pertusum [file bdj-09-e60548-s001.zip › images_new/000203 TMBL-ROV 2000 sa╠êcken Tape 56_frame_12775.jpg]

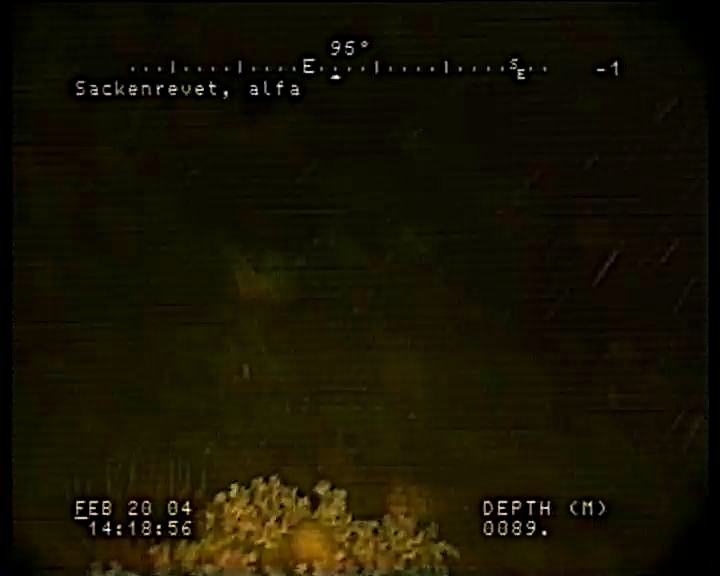

Supplement: Supplementary material 1 — Dataset of underwater images of Desmophyllum pertusum [file bdj-09-e60548-s001.zip › images_new/040220 TMBL-ROV 2004 Sa╠êckenrevet alfa_frame_63750.jpg]

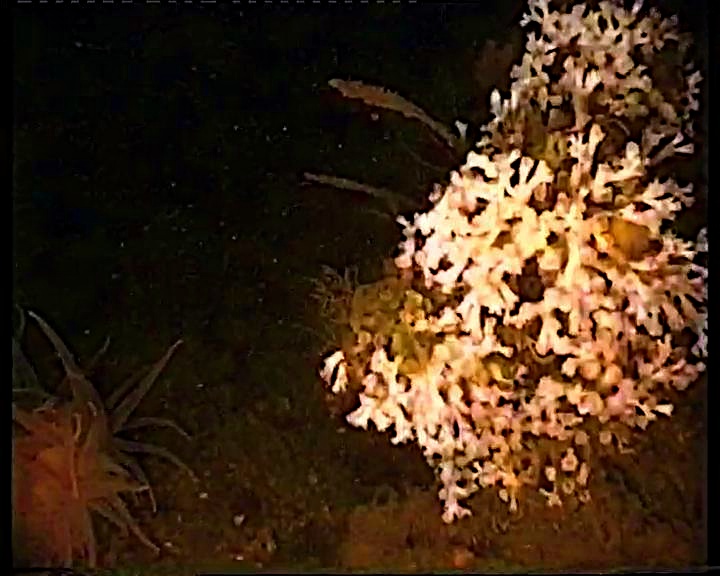

Supplement: Supplementary material 1 — Dataset of underwater images of Desmophyllum pertusum [file bdj-09-e60548-s001.zip › images_new/000114 TMBL-ROV 2000 Sa╠êckenrevet EJ numrerade band_frame_35250.jpg]

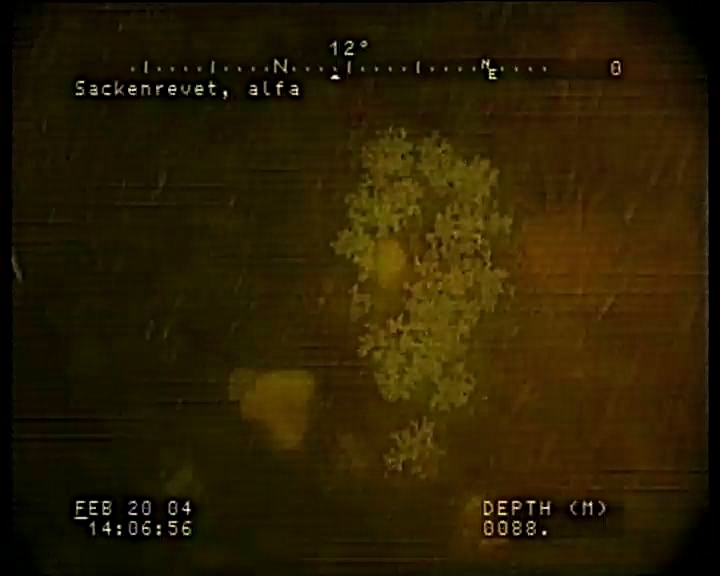

Supplement: Supplementary material 1 — Dataset of underwater images of Desmophyllum pertusum [file bdj-09-e60548-s001.zip › images_new/040220 TMBL-ROV 2004 Sa╠êckenrevet alfa_frame_45750.jpg]

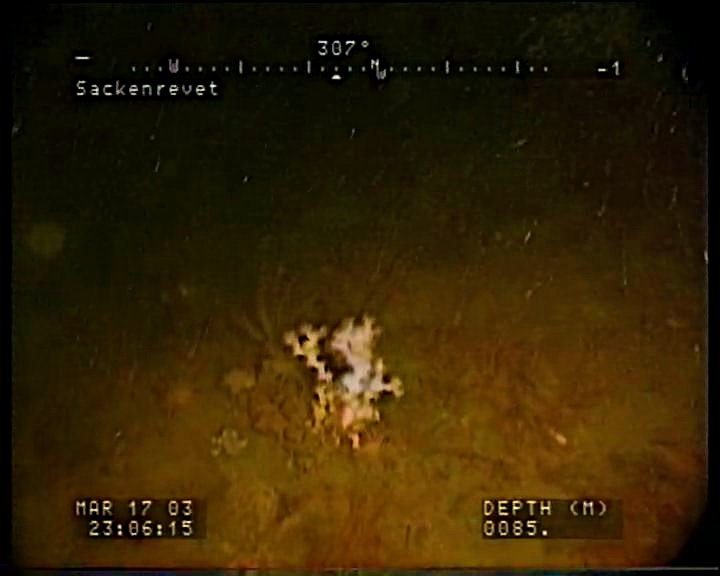

Supplement: Supplementary material 1 — Dataset of underwater images of Desmophyllum pertusum [file bdj-09-e60548-s001.zip › images_new/030317-18 TMBL-ROV 2003 Sa╠êckenrevet_frame_7525.jpg]

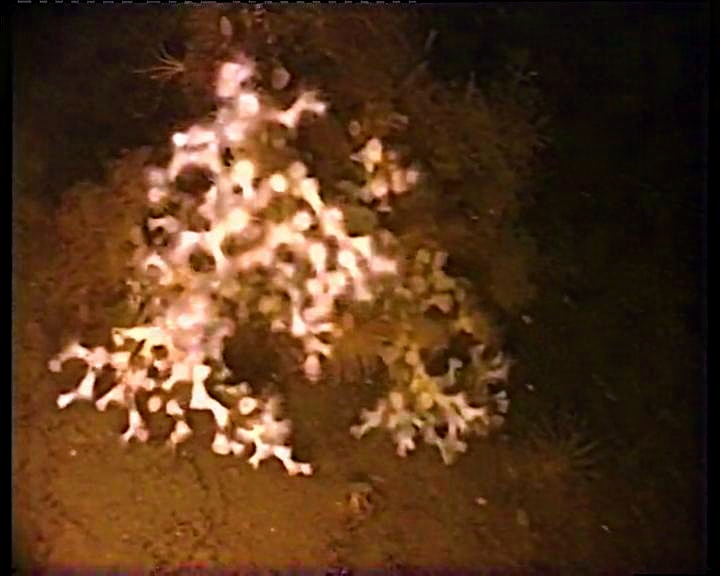

Supplement: Supplementary material 1 — Dataset of underwater images of Desmophyllum pertusum [file bdj-09-e60548-s001.zip › images_new/000203 TMBL-ROV 2000 Sa╠êcken revet EJ numrerade band_frame_22525.jpg]

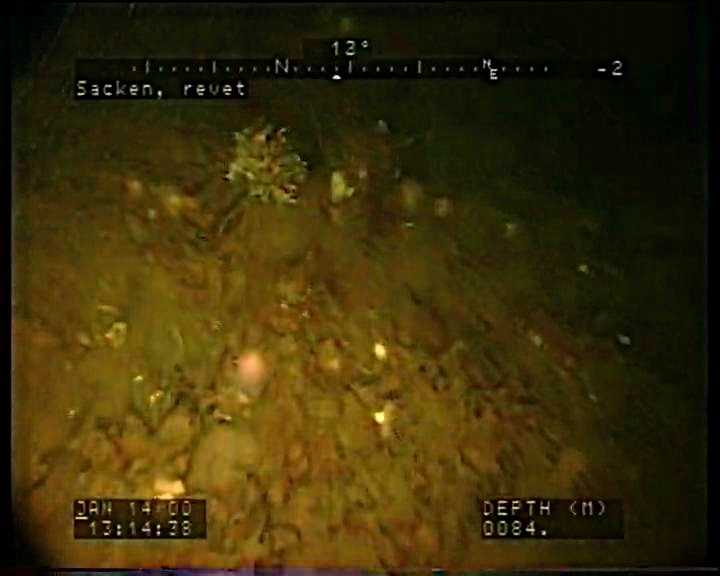

Supplement: Supplementary material 1 — Dataset of underwater images of Desmophyllum pertusum [file bdj-09-e60548-s001.zip › images_new/000114 TMBL-ROV 2000 Sa╠êckenrevet Tape 55_frame_56275.jpg]

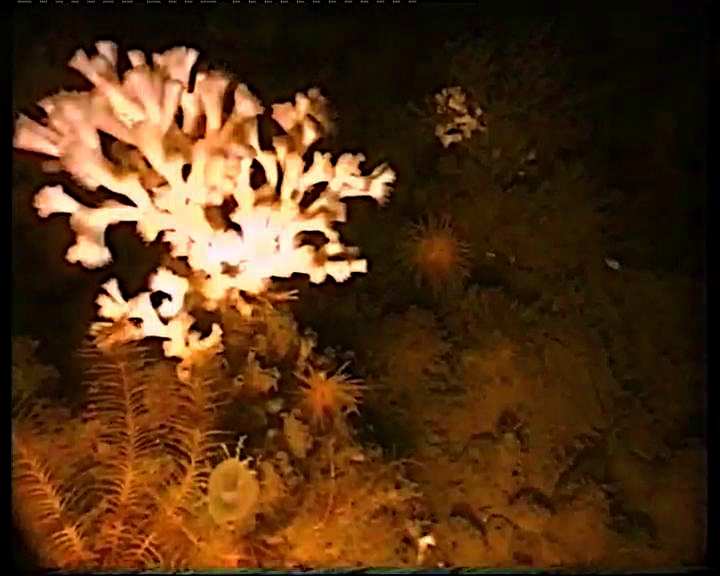

Supplement: Supplementary material 1 — Dataset of underwater images of Desmophyllum pertusum [file bdj-09-e60548-s001.zip › images_new/000203 TMBL-ROV 2000 Sa╠êcken revet EJ numrerade band_frame_42762.jpg]

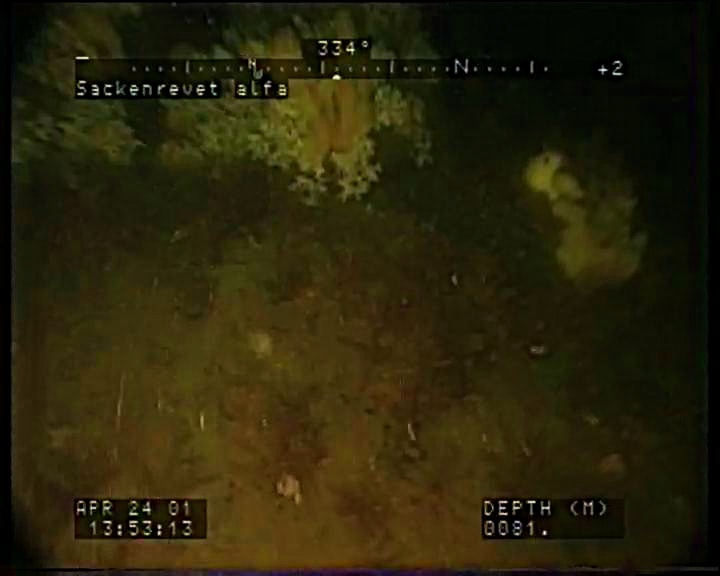

Supplement: Supplementary material 1 — Dataset of underwater images of Desmophyllum pertusum [file bdj-09-e60548-s001.zip › images_new/010424 Sa╠êckenrevet alfa Tape 74_frame_59375.jpg]

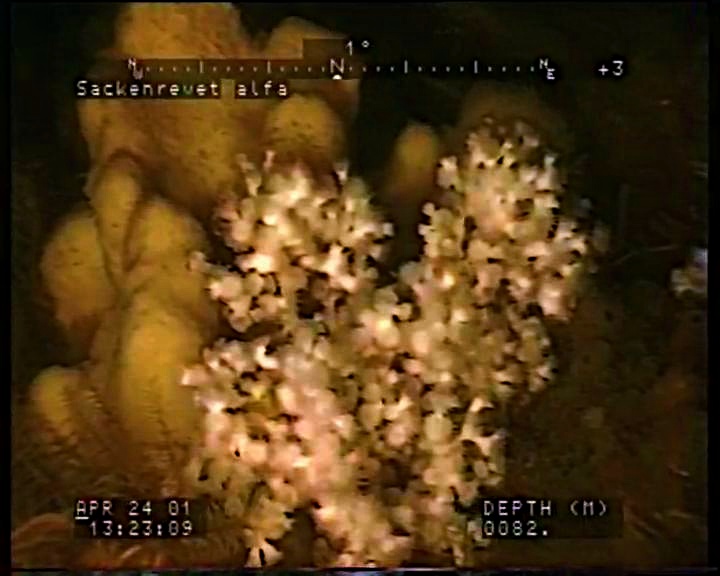

Supplement: Supplementary material 1 — Dataset of underwater images of Desmophyllum pertusum [file bdj-09-e60548-s001.zip › images_new/010424 Sa╠êckenrevet alfa Tape 74_frame_14275.jpg]

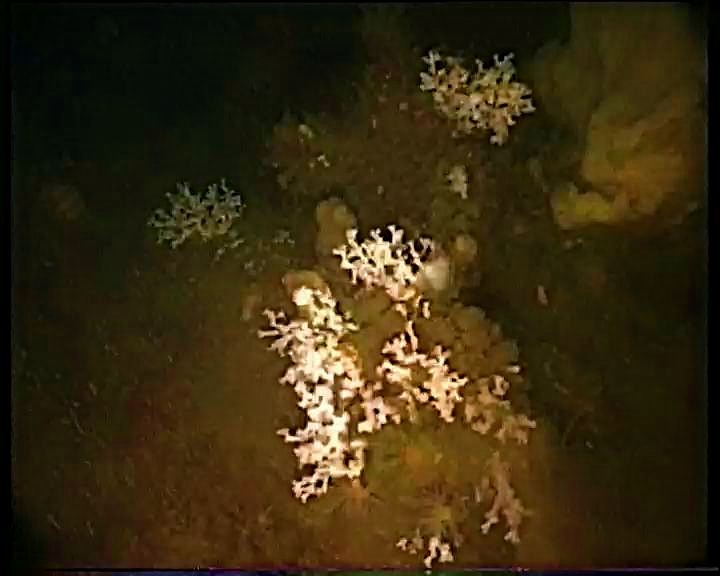

Supplement: Supplementary material 1 — Dataset of underwater images of Desmophyllum pertusum [file bdj-09-e60548-s001.zip › images_new/000203 TMBL-ROV 2000 Sa╠êckenrevet Tape 56_frame_33000.jpg]

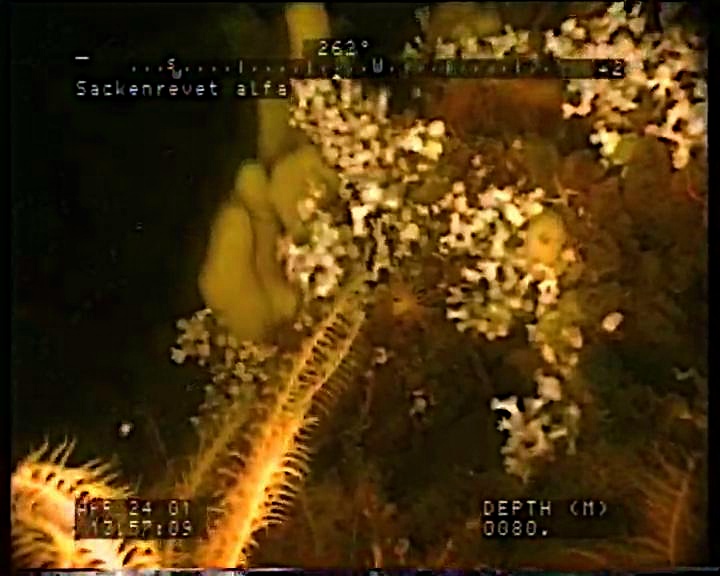

Supplement: Supplementary material 1 — Dataset of underwater images of Desmophyllum pertusum [file bdj-09-e60548-s001.zip › images_new/010424 Sa╠êckenrevet alfa Tape 74_frame_65275.jpg]

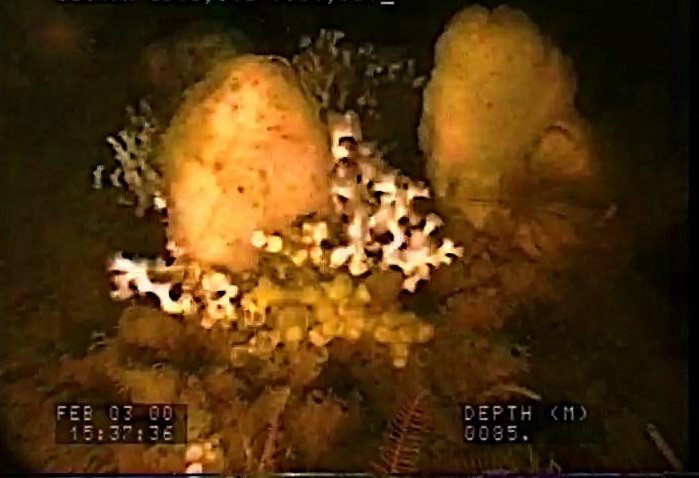

Supplement: Supplementary material 1 — Dataset of underwater images of Desmophyllum pertusum [file bdj-09-e60548-s001.zip › images_new/000203 TMBL-ROV 2000 Sa╠êckenrevet Tape 56_frame_42000.jpg]

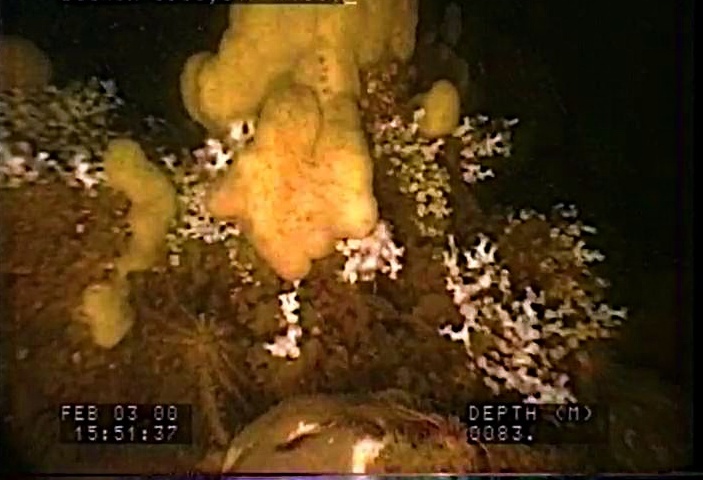

Supplement: Supplementary material 1 — Dataset of underwater images of Desmophyllum pertusum [file bdj-09-e60548-s001.zip › images_new/000203 TMBL-ROV 2000 Sa╠êckenrevet Tape 56_frame_63025.jpg]

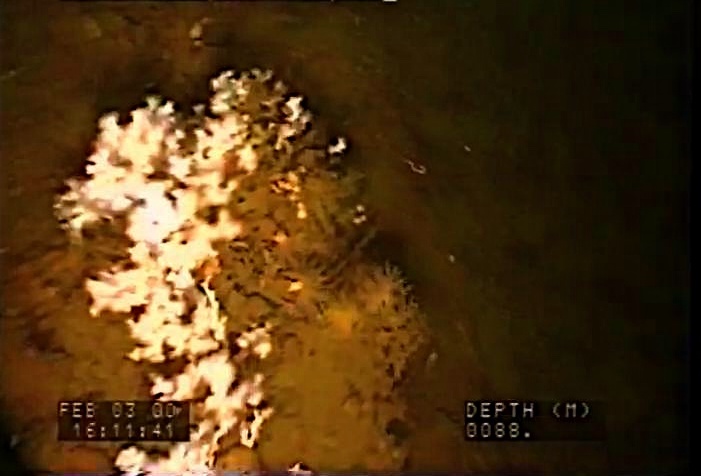

Supplement: Supplementary material 1 — Dataset of underwater images of Desmophyllum pertusum [file bdj-09-e60548-s001.zip › images_new/000203 TMBL-ROV 2000 Sa╠êcken revet EJ numrerade band_frame_57025.jpg]

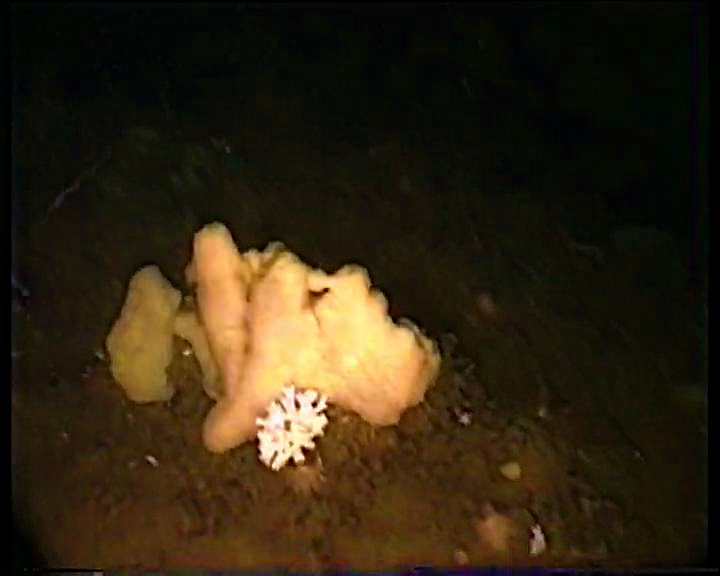

Supplement: Supplementary material 1 — Dataset of underwater images of Desmophyllum pertusum [file bdj-09-e60548-s001.zip › images_new/990506 TMBL-ROV 1999 Revet Sa╠êcken 2 Tape 42_frame_27075.jpg]

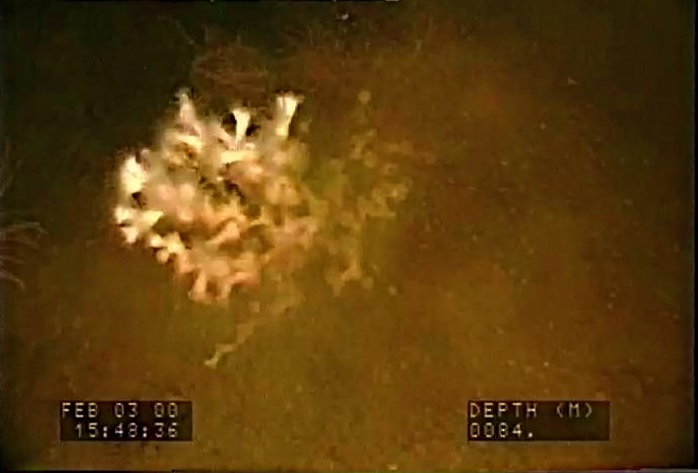

Supplement: Supplementary material 1 — Dataset of underwater images of Desmophyllum pertusum [file bdj-09-e60548-s001.zip › images_new/000203 TMBL-ROV 2000 Sa╠êckenrevet Tape 56_frame_58500.jpg]

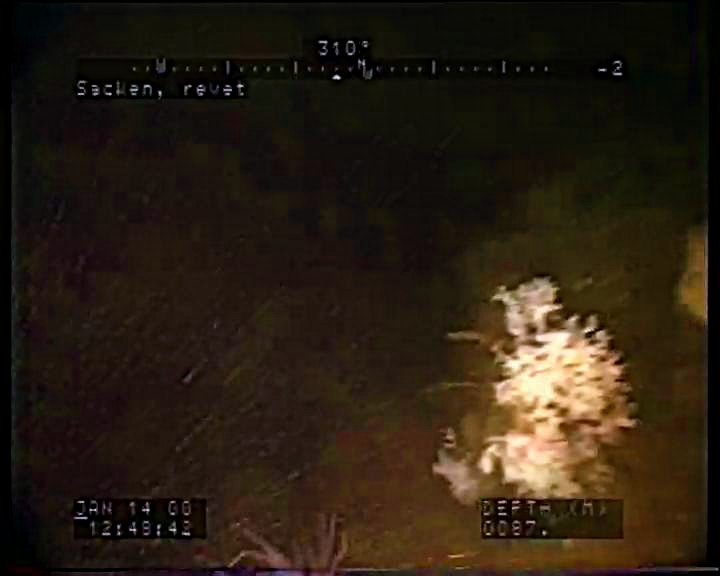

Supplement: Supplementary material 1 — Dataset of underwater images of Desmophyllum pertusum [file bdj-09-e60548-s001.zip › images_new/000114 TMBL-ROV 2000 Sa╠êckenrevet Tape 55_frame_17375.jpg]

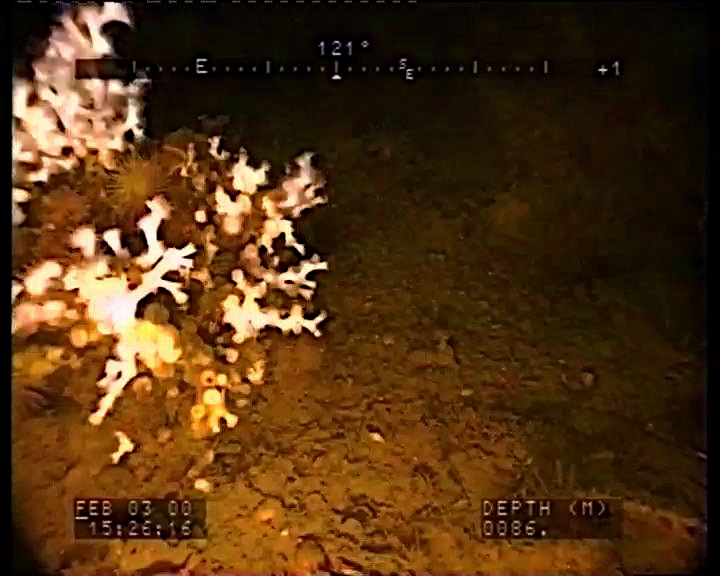

Supplement: Supplementary material 1 — Dataset of underwater images of Desmophyllum pertusum [file bdj-09-e60548-s001.zip › images_new/000203 TMBL-ROV 2000 Sa╠êcken revet EJ numrerade band_frame_1500.jpg]

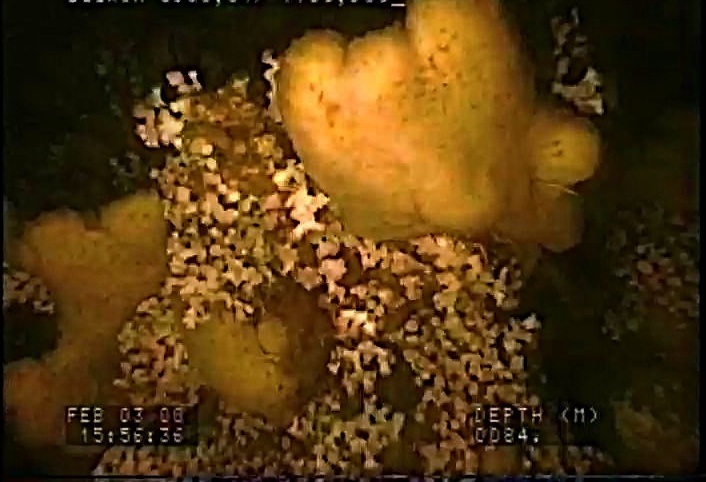

Supplement: Supplementary material 1 — Dataset of underwater images of Desmophyllum pertusum [file bdj-09-e60548-s001.zip › images_new/000203 TMBL-ROV 2000 Sa╠êckenrevet Tape 56_frame_70500.jpg]

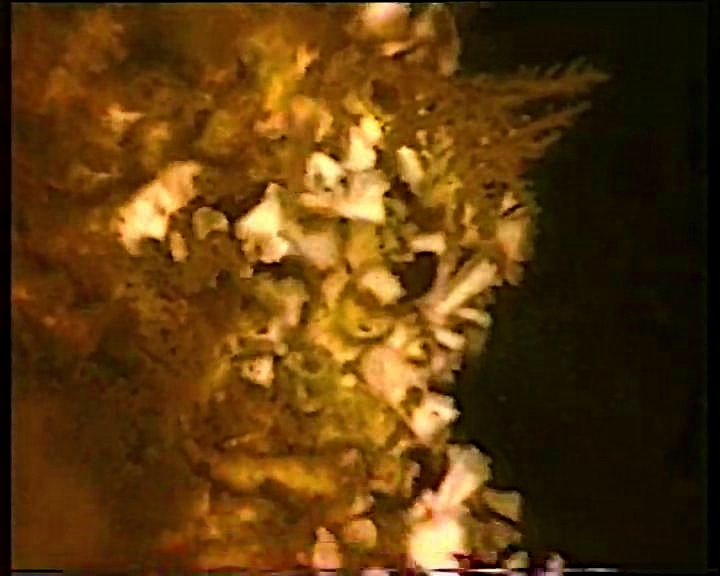

Supplement: Supplementary material 1 — Dataset of underwater images of Desmophyllum pertusum [file bdj-09-e60548-s001.zip › images_new/990506 TMBL-ROV 1999 Revet Sa╠êcken 2 Tape 42_frame_7550.jpg]

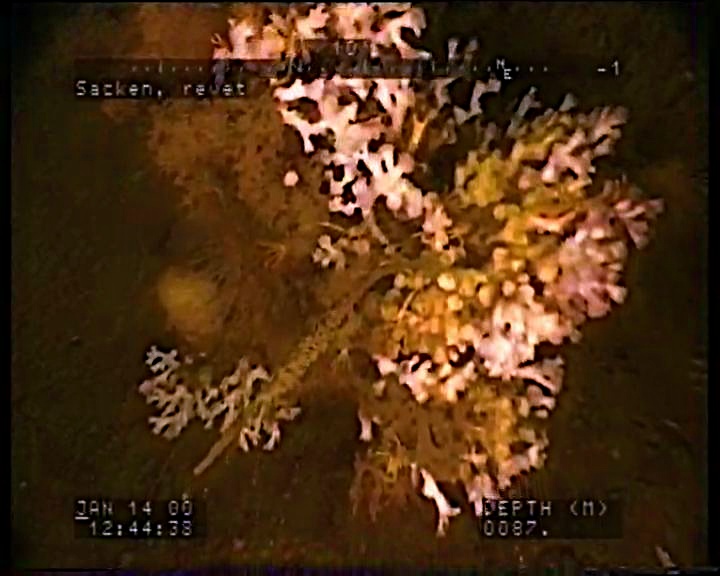

Supplement: Supplementary material 1 — Dataset of underwater images of Desmophyllum pertusum [file bdj-09-e60548-s001.zip › images_new/000114 TMBL-ROV 2000 Sa╠êckenrevet Tape 55_frame_11275.jpg]

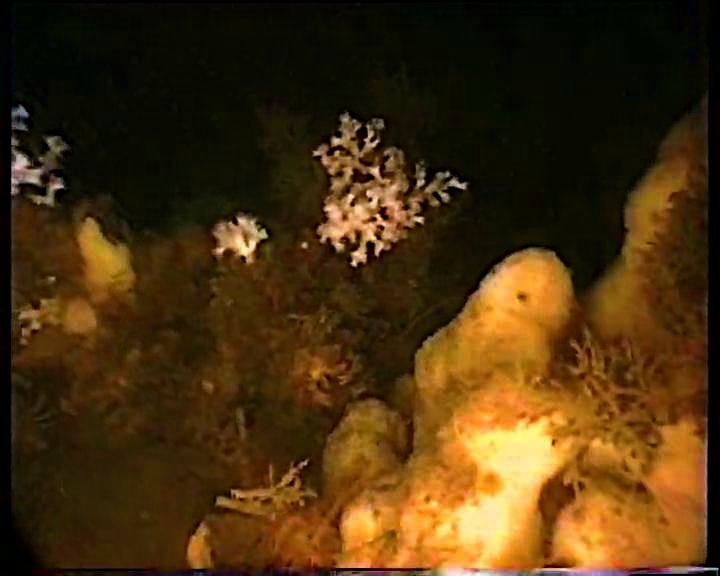

Supplement: Supplementary material 1 — Dataset of underwater images of Desmophyllum pertusum [file bdj-09-e60548-s001.zip › images_new/000203 TMBL-ROV 2000 Sa╠êckenrevet Tape 56_frame_36025.jpg]

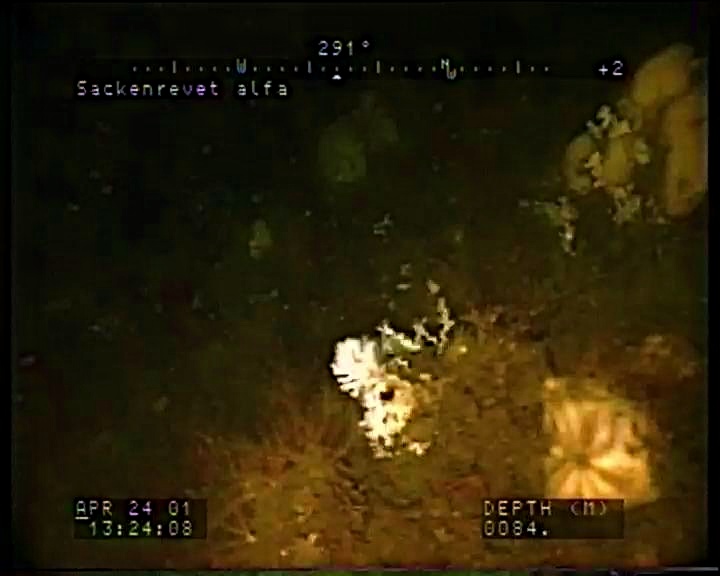

Supplement: Supplementary material 1 — Dataset of underwater images of Desmophyllum pertusum [file bdj-09-e60548-s001.zip › images_new/010424 Sa╠êckenrevet alfa Tape 74_frame_15750.jpg]

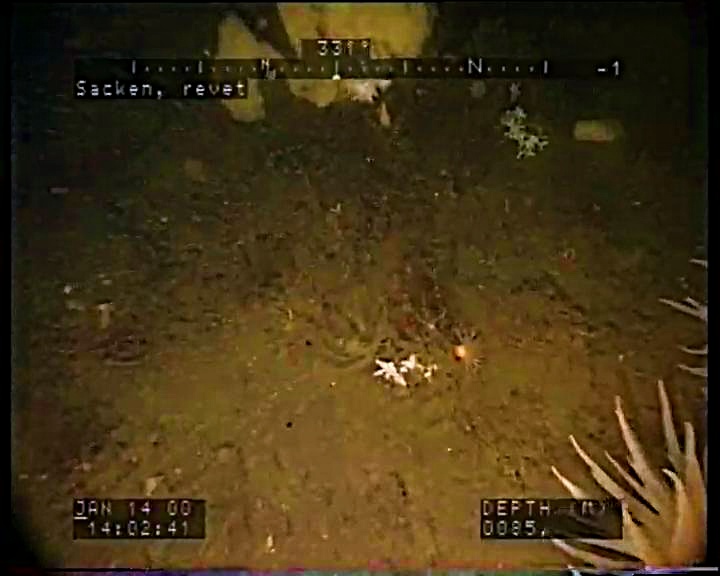

Supplement: Supplementary material 1 — Dataset of underwater images of Desmophyllum pertusum [file bdj-09-e60548-s001.zip › images_new/000114 TMBL-ROV 2000 Sa╠êckenrevet Tape 55_frame_128350.jpg]

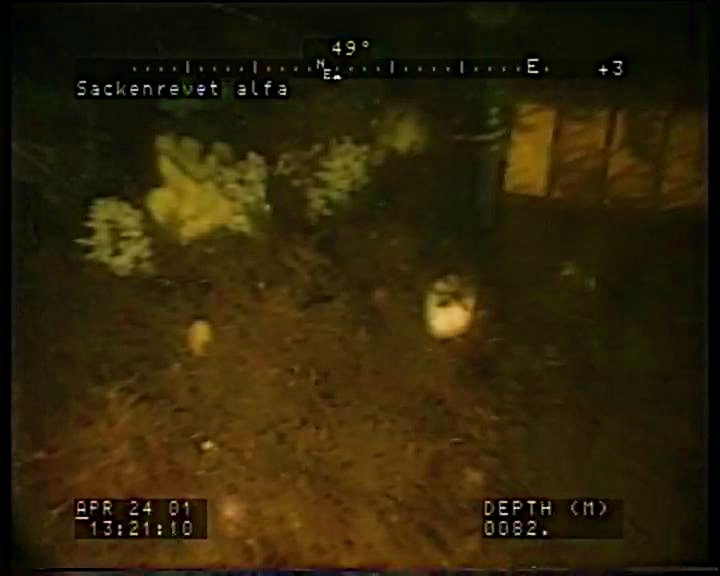

Supplement: Supplementary material 1 — Dataset of underwater images of Desmophyllum pertusum [file bdj-09-e60548-s001.zip › images_new/010424 Sa╠êckenrevet alfa Tape 74_frame_11287.jpg]

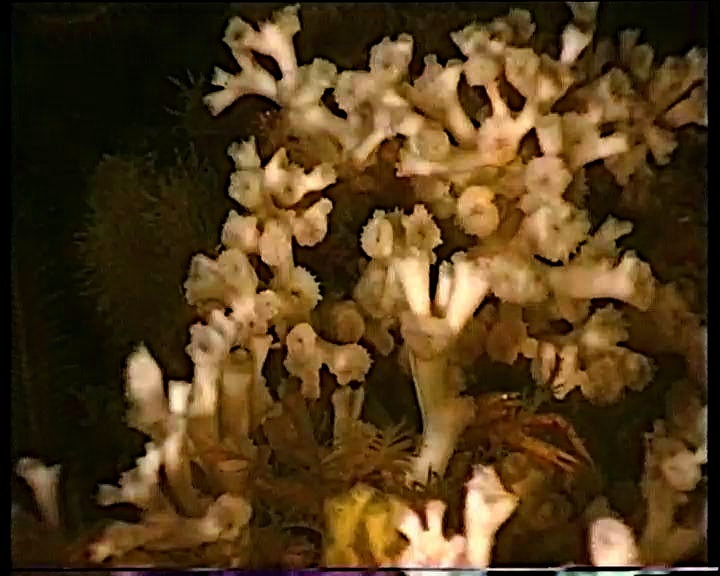

Supplement: Supplementary material 1 — Dataset of underwater images of Desmophyllum pertusum [file bdj-09-e60548-s001.zip › images_new/000114 TMBL-ROV 2000 Sa╠êckenrevet Tape 55_frame_195000.jpg]

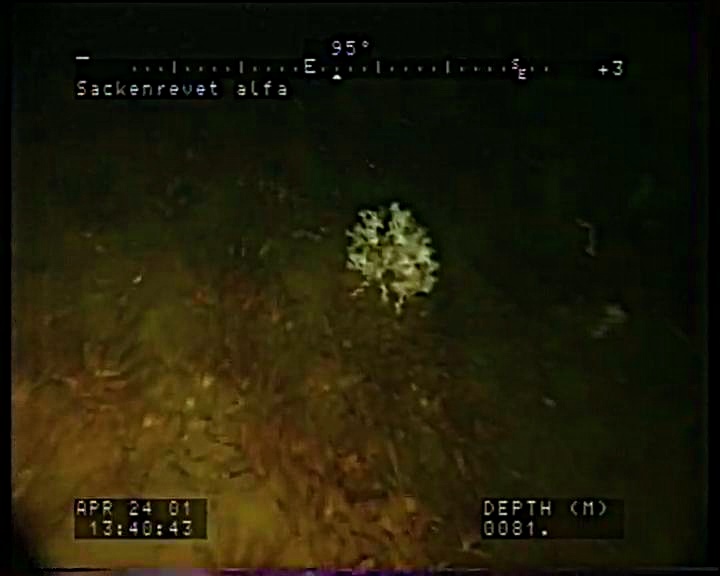

Supplement: Supplementary material 1 — Dataset of underwater images of Desmophyllum pertusum [file bdj-09-e60548-s001.zip › images_new/010424 Sa╠êckenrevet alfa Tape 74_frame_40625.jpg]

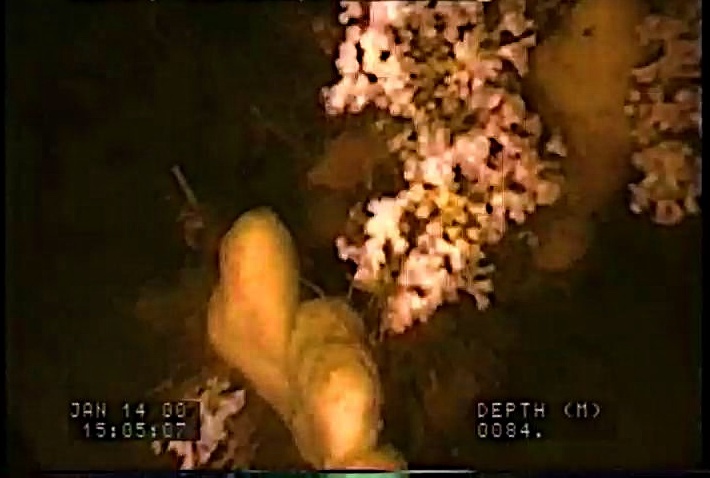

Supplement: Supplementary material 1 — Dataset of underwater images of Desmophyllum pertusum [file bdj-09-e60548-s001.zip › images_new/000114 TMBL-ROV 2000 Sa╠êckenrevet Tape 55_frame_222000.jpg]

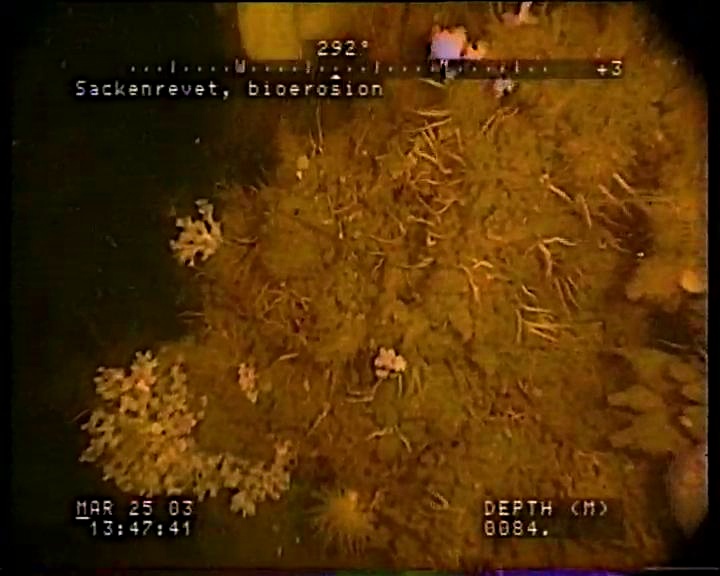

Supplement: Supplementary material 1 — Dataset of underwater images of Desmophyllum pertusum [file bdj-09-e60548-s001.zip › images_new/030325 TMBL-ROV 2003 Sa╠êckenrevet bioerosion_frame_79525.jpg]

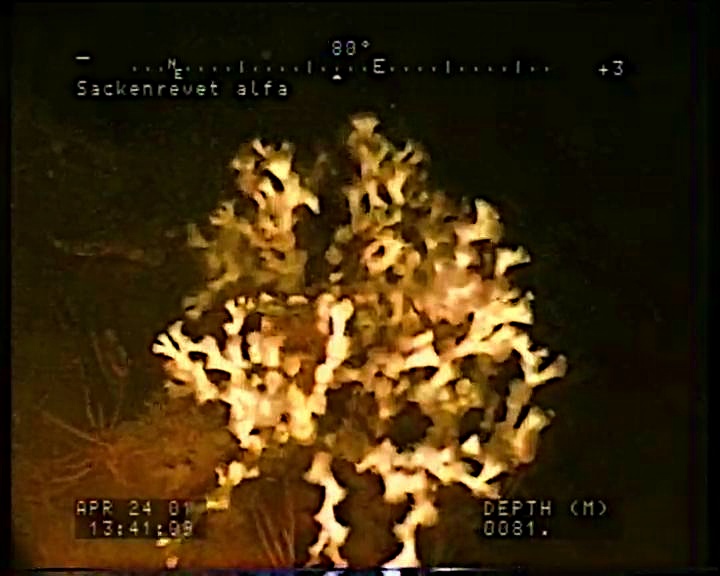

Supplement: Supplementary material 1 — Dataset of underwater images of Desmophyllum pertusum [file bdj-09-e60548-s001.zip › images_new/010424 Sa╠êckenrevet alfa Tape 74_frame_41275.jpg]

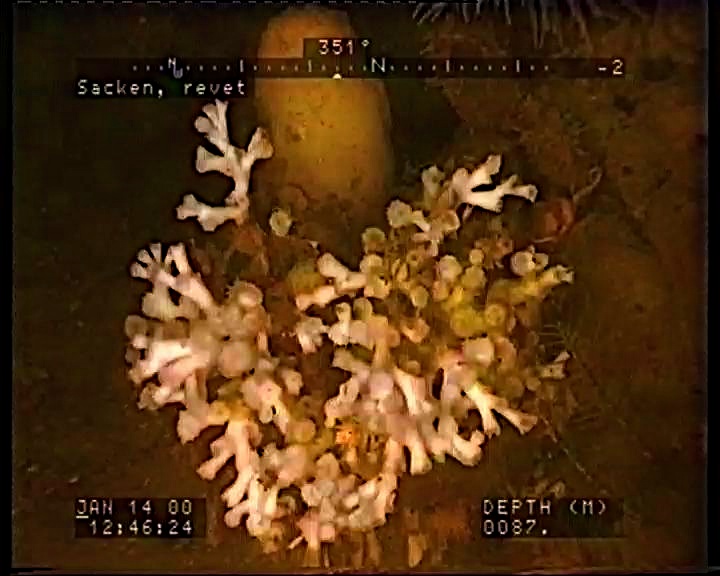

Supplement: Supplementary material 1 — Dataset of underwater images of Desmophyllum pertusum [file bdj-09-e60548-s001.zip › images_new/000114 TMBL-ROV 2000 Sa╠êckenrevet EJ numrerade band_frame_4500.jpg]

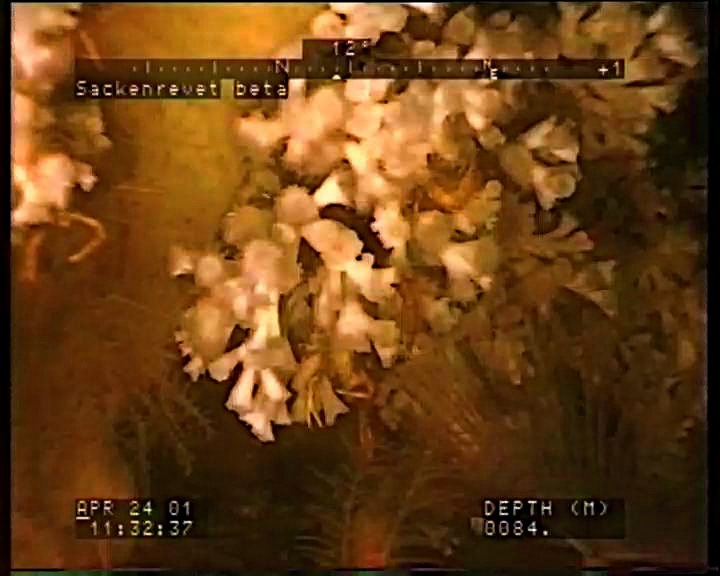

Supplement: Supplementary material 1 — Dataset of underwater images of Desmophyllum pertusum [file bdj-09-e60548-s001.zip › images_new/010424 Sa╠êckenrevet beta Tape 74_frame_47250.jpg]

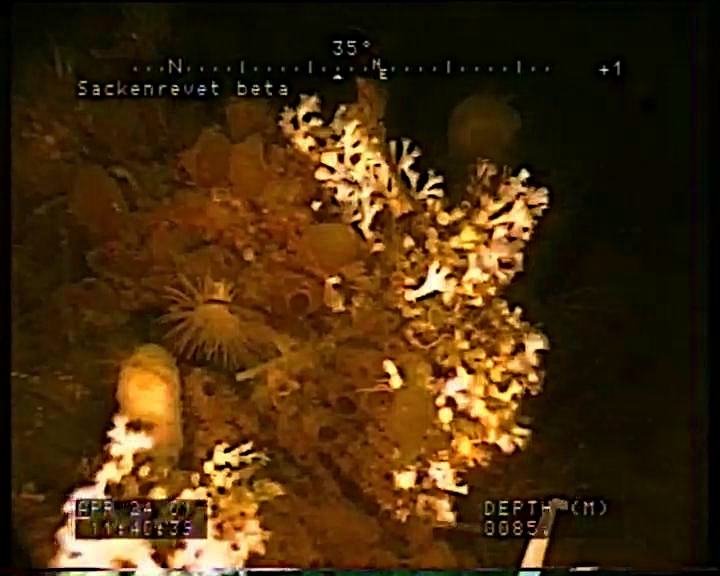

Supplement: Supplementary material 1 — Dataset of underwater images of Desmophyllum pertusum [file bdj-09-e60548-s001.zip › images_new/010424 Sa╠êckenrevet beta Tape 74_frame_59287.jpg]

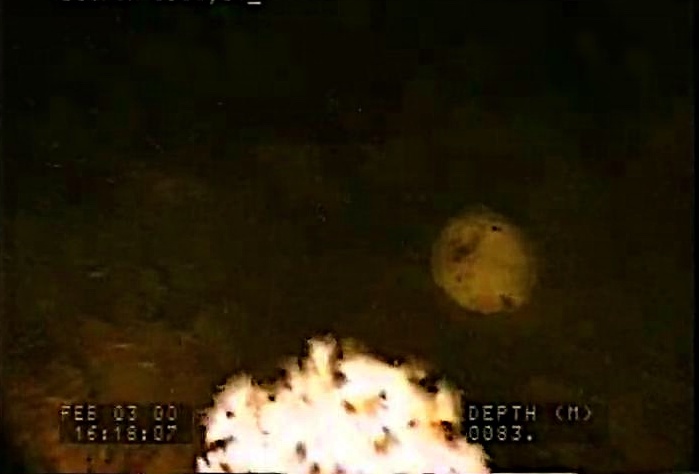

Supplement: Supplementary material 1 — Dataset of underwater images of Desmophyllum pertusum [file bdj-09-e60548-s001.zip › images_new/000203 TMBL-ROV 2000 Sa╠êckenrevet Tape 56_frame_102775.jpg]

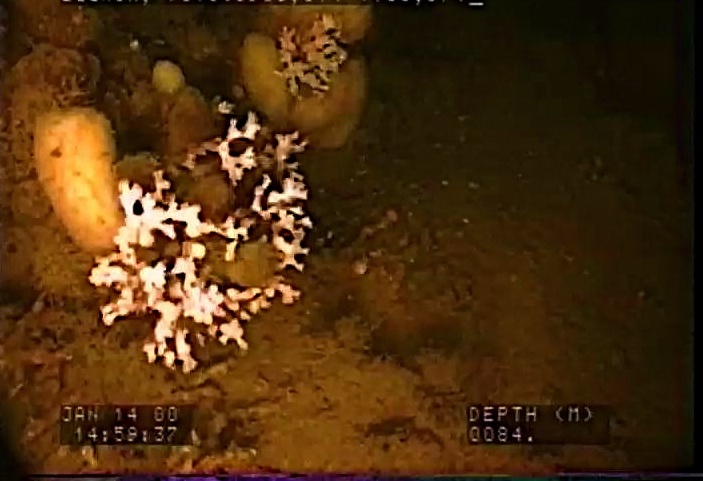

Supplement: Supplementary material 1 — Dataset of underwater images of Desmophyllum pertusum [file bdj-09-e60548-s001.zip › images_new/000114 TMBL-ROV 2000 Sa╠êckenrevet Tape 55_frame_213750.jpg]

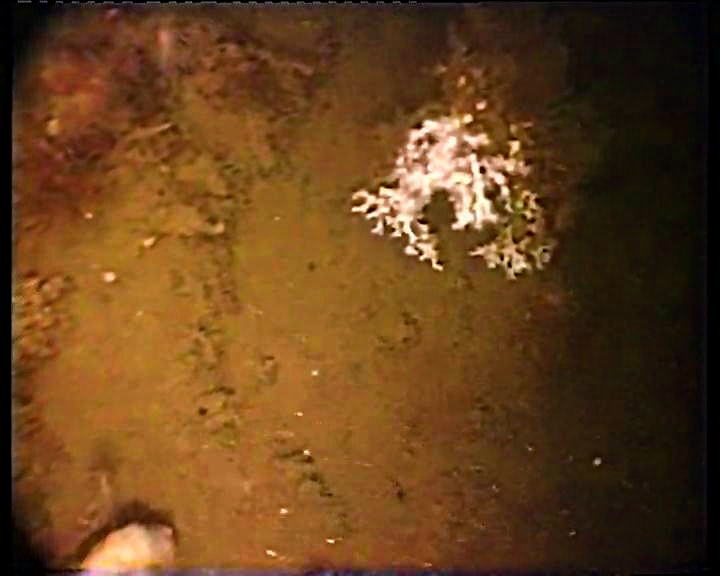

Supplement: Supplementary material 1 — Dataset of underwater images of Desmophyllum pertusum [file bdj-09-e60548-s001.zip › images_new/000203 TMBL-ROV 2000 Sa╠êcken revet EJ numrerade band_frame_21775.jpg]

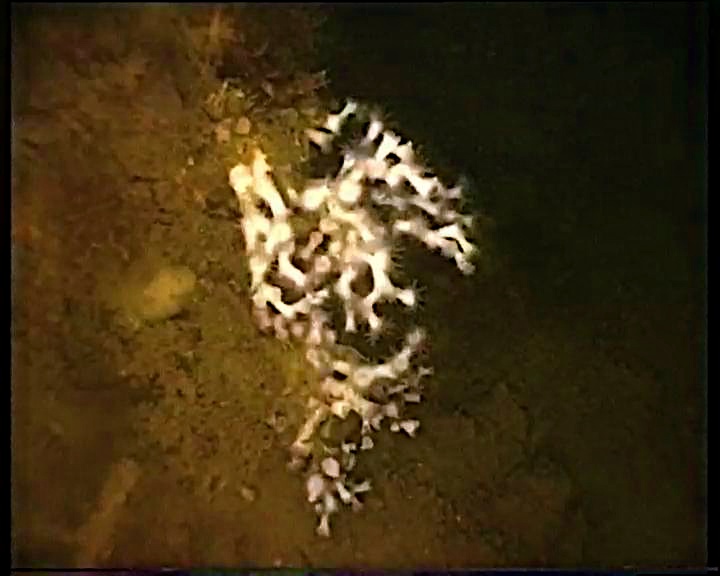

Supplement: Supplementary material 1 — Dataset of underwater images of Desmophyllum pertusum [file bdj-09-e60548-s001.zip › images_new/000203 TMBL-ROV 2000 Sa╠êckenrevet Tape 56_frame_53287.jpg]

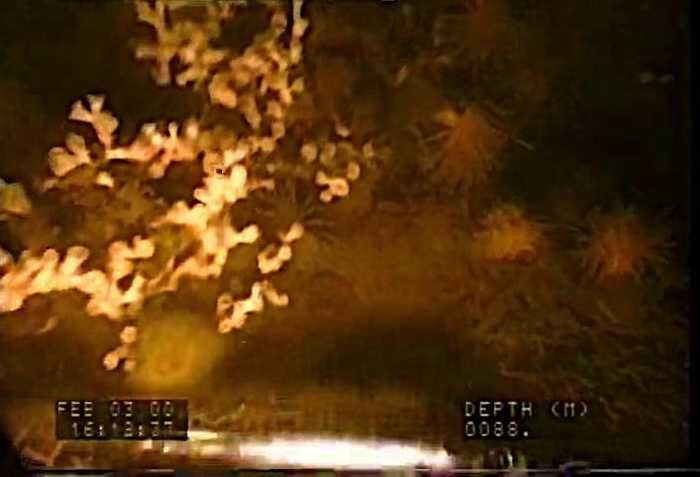

Supplement: Supplementary material 1 — Dataset of underwater images of Desmophyllum pertusum [file bdj-09-e60548-s001.zip › images_new/000203 TMBL-ROV 2000 Sa╠êckenrevet Tape 56_frame_94525.jpg]

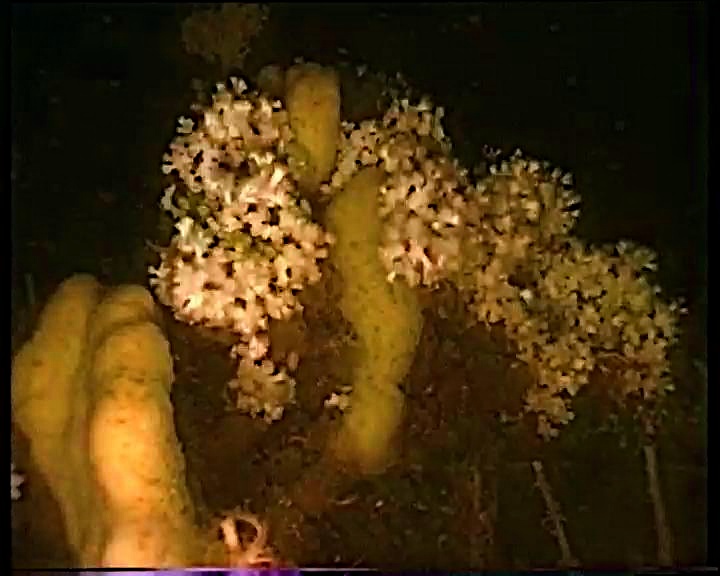

Supplement: Supplementary material 1 — Dataset of underwater images of Desmophyllum pertusum [file bdj-09-e60548-s001.zip › images_new/000114 TMBL-ROV 2000 Sa╠êckenrevet Tape 55_frame_209250.jpg]

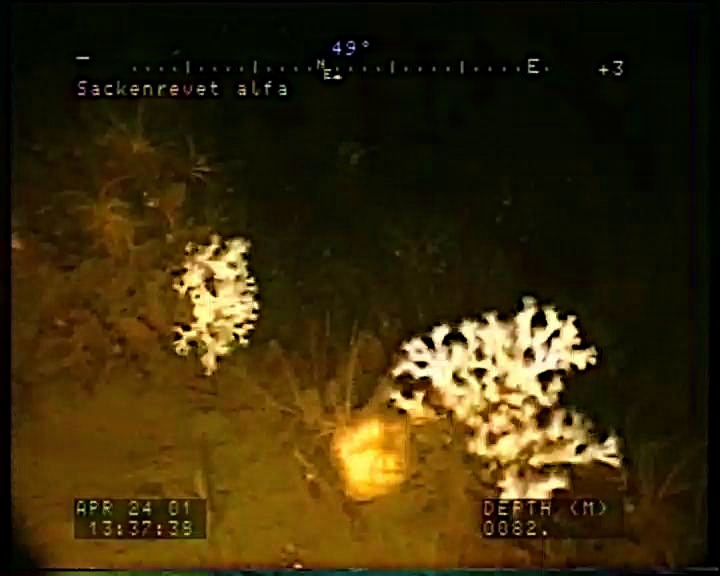

Supplement: Supplementary material 1 — Dataset of underwater images of Desmophyllum pertusum [file bdj-09-e60548-s001.zip › images_new/010424 Sa╠êckenrevet alfa Tape 74_frame_36000.jpg]

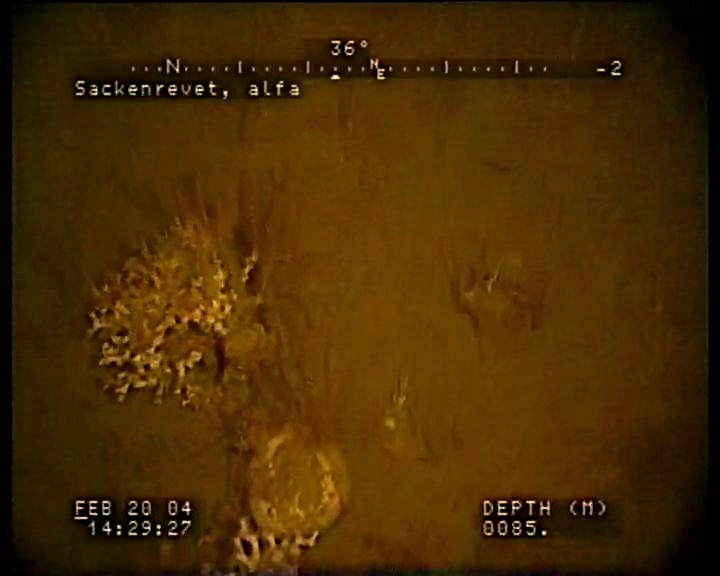

Supplement: Supplementary material 1 — Dataset of underwater images of Desmophyllum pertusum [file bdj-09-e60548-s001.zip › images_new/040220 TMBL-ROV 2004 Sa╠êckenrevet alfa_frame_79525.jpg]

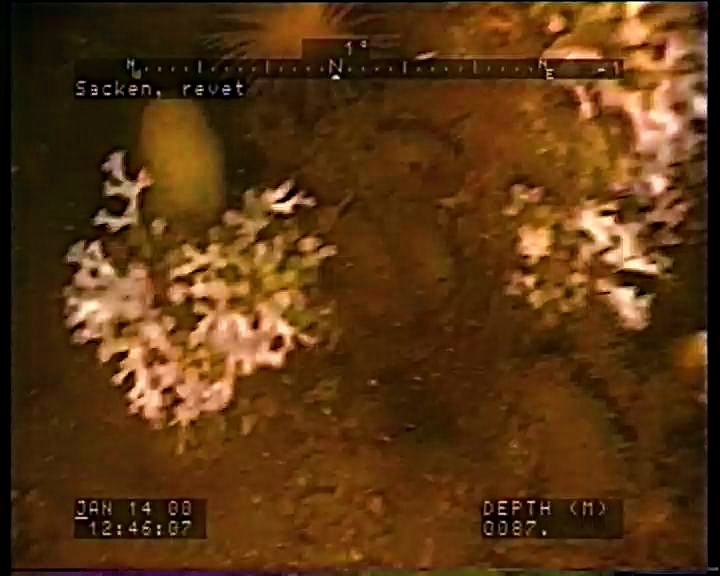

Supplement: Supplementary material 1 — Dataset of underwater images of Desmophyllum pertusum [file bdj-09-e60548-s001.zip › images_new/000114 TMBL-ROV 2000 Sa╠êckenrevet Tape 55_frame_13500.jpg]

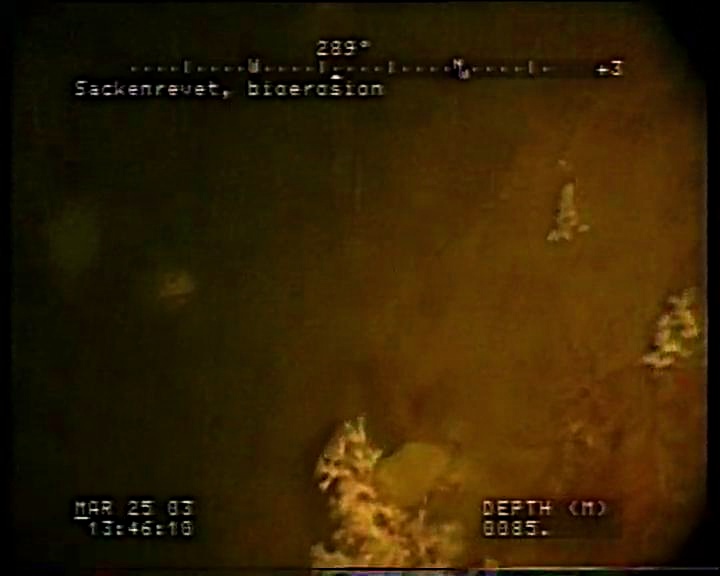

Supplement: Supplementary material 1 — Dataset of underwater images of Desmophyllum pertusum [file bdj-09-e60548-s001.zip › images_new/030325 TMBL-ROV 2003 Sa╠êckenrevet bioerosion_frame_77250.jpg]

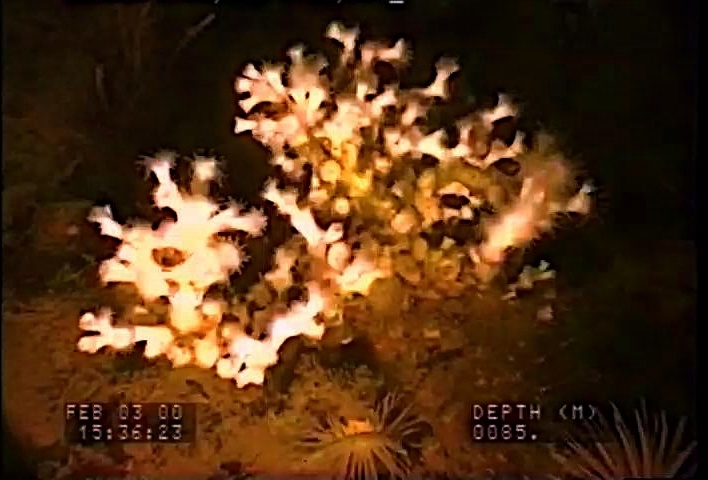

Supplement: Supplementary material 1 — Dataset of underwater images of Desmophyllum pertusum [file bdj-09-e60548-s001.zip › images_new/000203 TMBL-ROV 2000 Sa╠êcken revet EJ numrerade band_frame_12750.jpg]

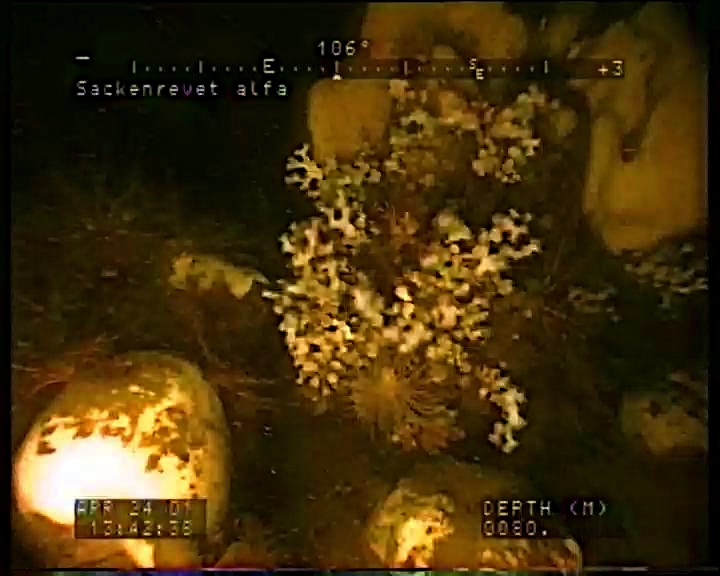

Supplement: Supplementary material 1 — Dataset of underwater images of Desmophyllum pertusum [file bdj-09-e60548-s001.zip › images_new/010424 Sa╠êckenrevet alfa Tape 74_frame_43500.jpg]

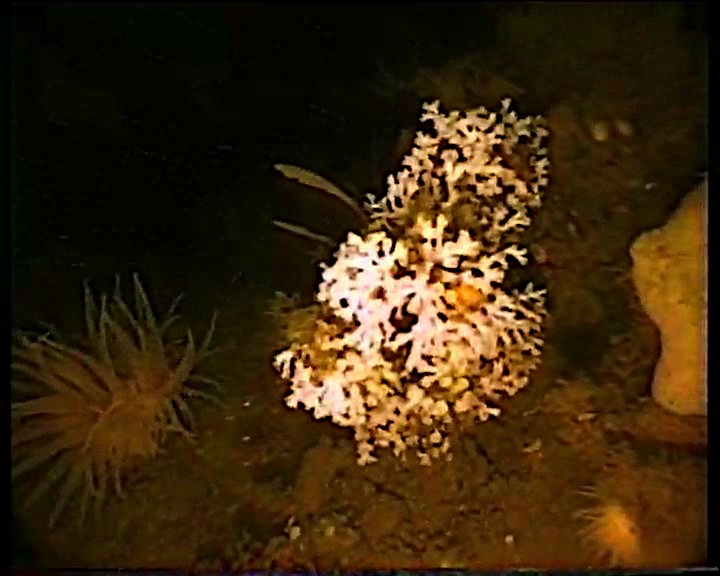

Supplement: Supplementary material 1 — Dataset of underwater images of Desmophyllum pertusum [file bdj-09-e60548-s001.zip › images_new/000114 TMBL-ROV 2000 Sa╠êckenrevet Tape 55_frame_224275.jpg]

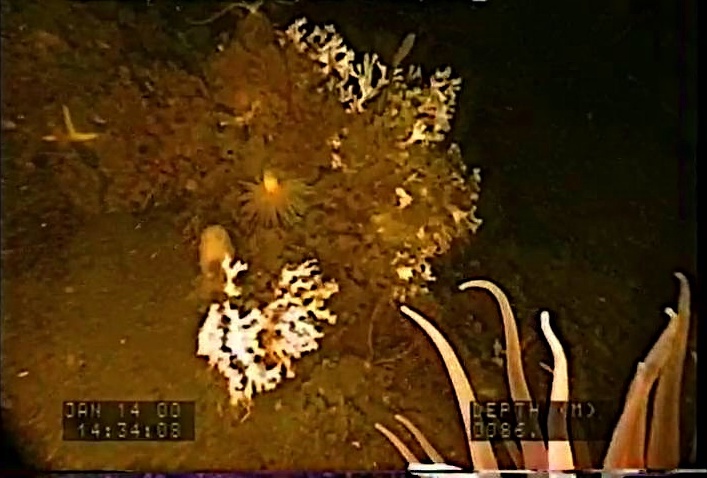

Supplement: Supplementary material 1 — Dataset of underwater images of Desmophyllum pertusum [file bdj-09-e60548-s001.zip › images_new/000114 TMBL-ROV 2000 Sa╠êckenrevet Tape 55_frame_175525.jpg]

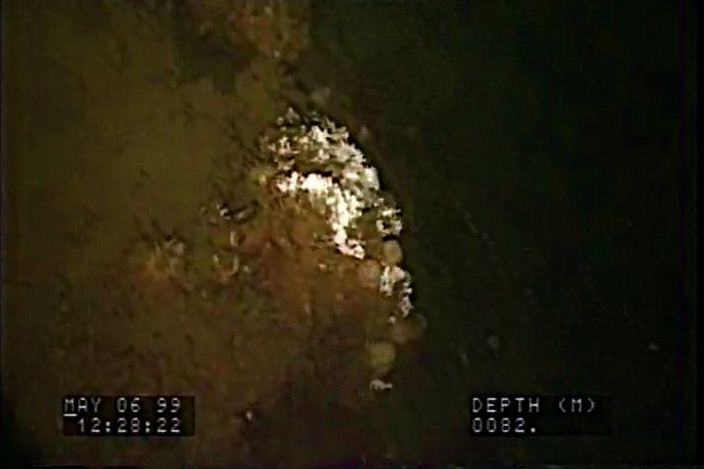

Supplement: Supplementary material 1 — Dataset of underwater images of Desmophyllum pertusum [file bdj-09-e60548-s001.zip › images_new/990506 TMBL-ROV 1999 Revet Sa╠êcken 2 Tape 42_frame_11275.jpg]

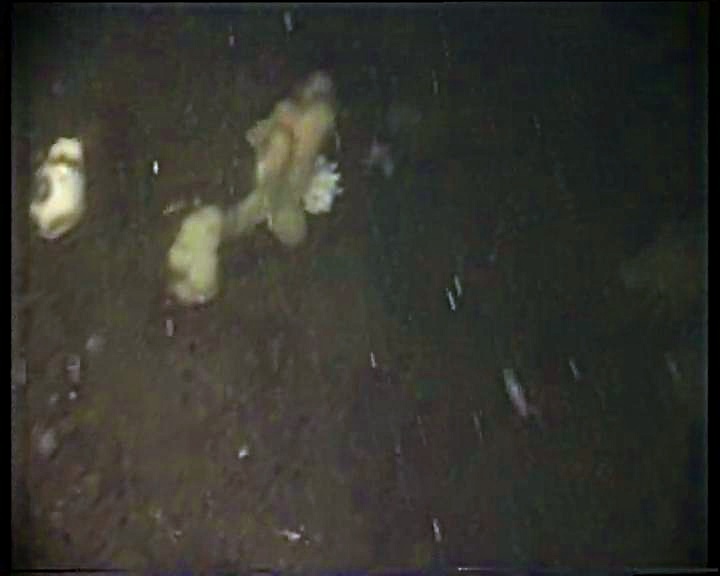

Supplement: Supplementary material 1 — Dataset of underwater images of Desmophyllum pertusum [file bdj-09-e60548-s001.zip › images_new/990506 TMBL-ROV 1999 Revet Sa╠êcken 2 Tape 42_frame_15775.jpg]

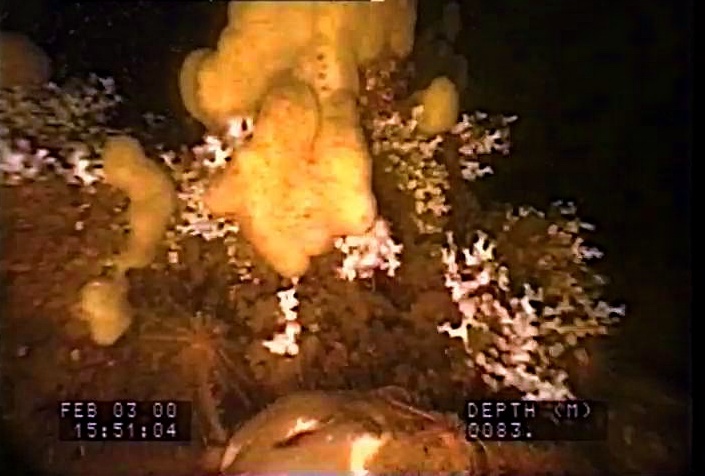

Supplement: Supplementary material 1 — Dataset of underwater images of Desmophyllum pertusum [file bdj-09-e60548-s001.zip › images_new/000203 TMBL-ROV 2000 Sa╠êcken revet EJ numrerade band_frame_29275.jpg]

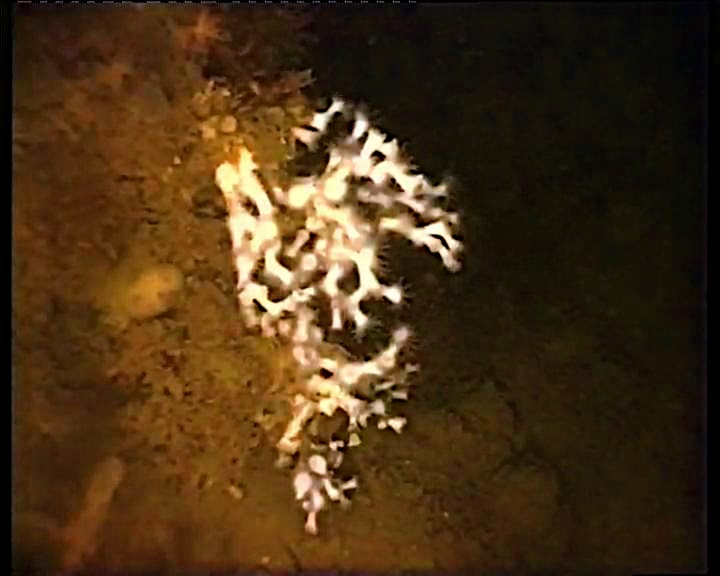

Supplement: Supplementary material 1 — Dataset of underwater images of Desmophyllum pertusum [file bdj-09-e60548-s001.zip › images_new/000203 TMBL-ROV 2000 Sa╠êcken revet EJ numrerade band_frame_24750.jpg]

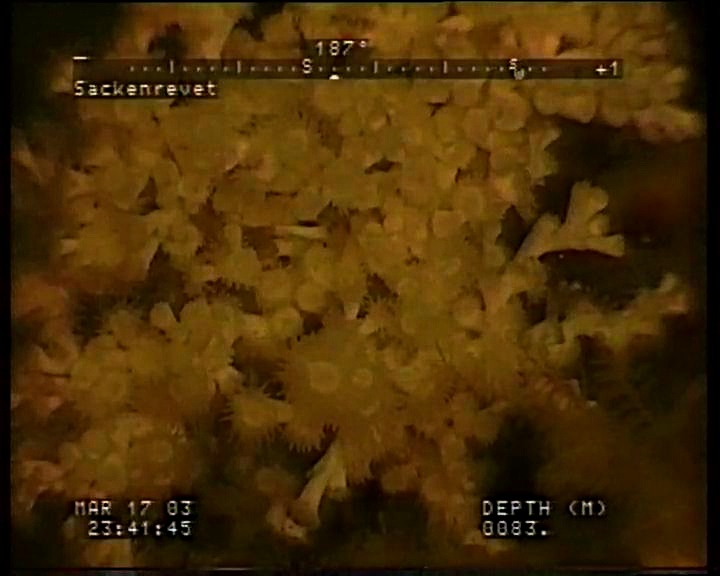

Supplement: Supplementary material 1 — Dataset of underwater images of Desmophyllum pertusum [file bdj-09-e60548-s001.zip › images_new/030317-18 TMBL-ROV 2003 Sa╠êckenrevet_frame_60775.jpg]

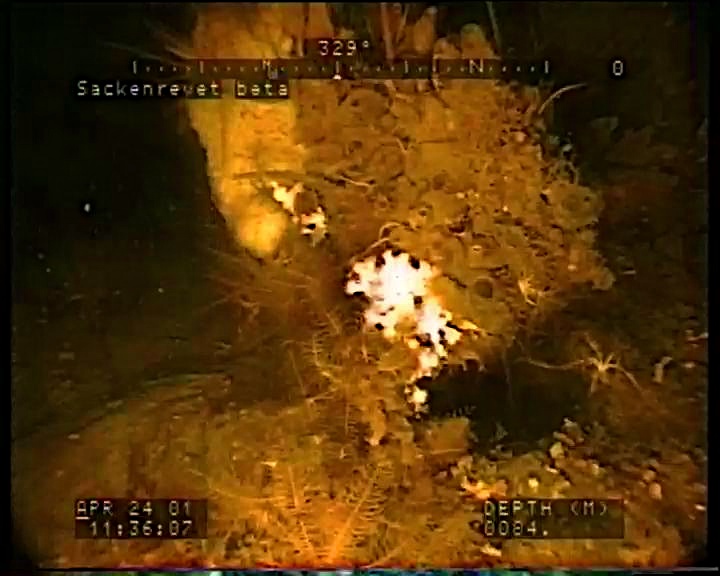

Supplement: Supplementary material 1 — Dataset of underwater images of Desmophyllum pertusum [file bdj-09-e60548-s001.zip › images_new/010424 Sa╠êckenrevet beta Tape 74_frame_52500.jpg]

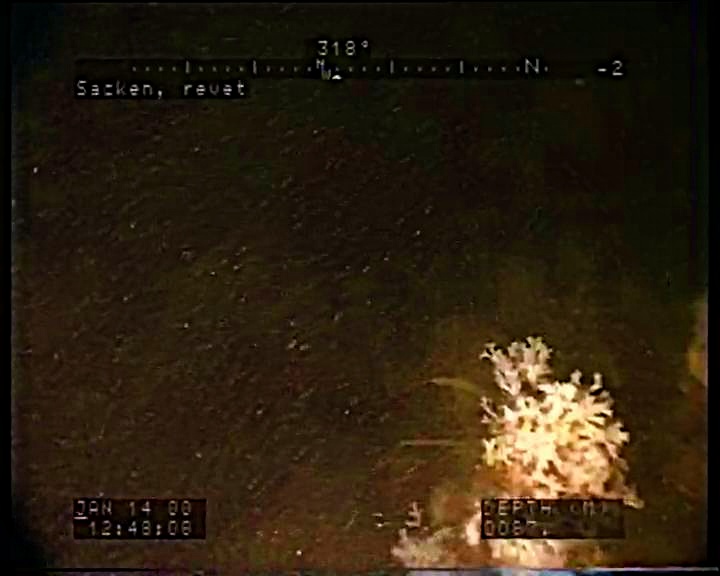

Supplement: Supplementary material 1 — Dataset of underwater images of Desmophyllum pertusum [file bdj-09-e60548-s001.zip › images_new/000114 TMBL-ROV 2000 Sa╠êckenrevet Tape 55_frame_16525.jpg]

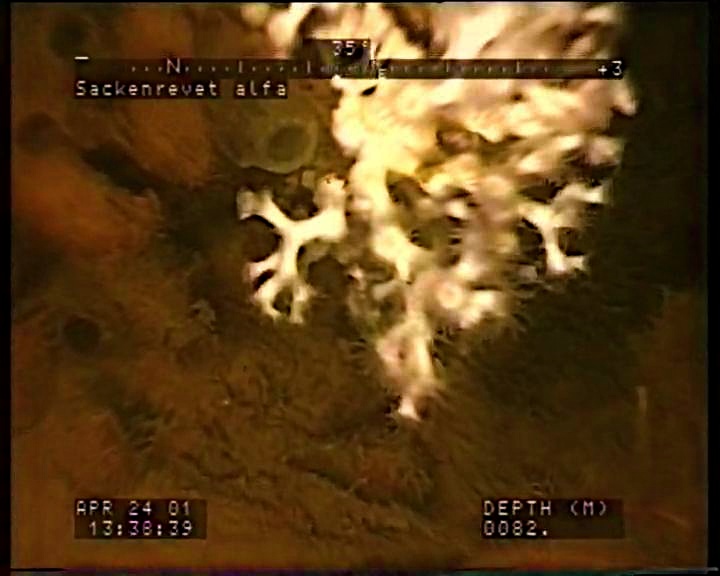

Supplement: Supplementary material 1 — Dataset of underwater images of Desmophyllum pertusum [file bdj-09-e60548-s001.zip › images_new/010424 Sa╠êckenrevet alfa Tape 74_frame_37525.jpg]
